# Patient-reported outcomes before treatment for localized prostate cancer: are there differences among countries? Data from the True North Global Registry

**DOI:** 10.1186/s12894-023-01344-0

**Published:** 2023-11-03

**Authors:** O. Garin, C. Kowalski, V. Zamora, R. Roth, M. Ferrer, C. Breidenbach, A. Pont, T. R. Belin, D. Elashoff, H. Wilhalme, A. V. Nguyen, L. Kwan, E. K. Pearman, A. Bolagani, F. Sampurno, N. Papa, C. Moore, J. Millar, S. E. Connor, P. Villanti, M. S. Litwin, True North Global Registry, Ian Graham, Christoph Kowalski, Mark S. Litwin, Jeremy Millar, Caroline Moore, Kellie Paich, Nikolajs Zeps, Sarah E. Connor, Anissa V. Nguyen, Krupa Krishnaprasad, Sibilah Breen, Silvi Brglevska, Michelle King, Barbara Avuzzi, Daniel Barocas, Alberto Briganti, Peter Chang, Montse Ferrer, Anthony Finelli, Claire Foster, Mark Frydenberg, Khurshid R. Ghani, Jeremy Grummet, Stephen Mark, Vincenzo Mirone, Dong-ho Mun, Colleen Nelson, Anthony Ng, David Pryor, Steven Siu, Phil Stricker, Jean-Paul van Basten, Andrew Vickers, Roman Zachoval

**Affiliations:** 1 Health Services Research Group, IMIM (Hospital del Mar Medical Research Institute) Barcelona Biomedical Research Park, Office 144, Doctor Aiguader 88, Barcelona, 08003 Spain; 2 CIBER en Epidemiología y Salud Pública, CIBERESP, Madrid, Spain; 3 Universitat Pompeu Fabra (UPF), Barcelona, Spain; 4 German Cancer Society, Berlin, Germany; 5 Universitat Autònoma de Barcelona (UAB), Bellaterra, Spain; 6 Institute of Medical Statistics and Computational Biology (IMSB), Medical Faculty, University of Cologne, Cologne, Germany; 7 University of California, Los Angeles, USA; 8 School of Public Health and Preventive Medicine, Monash University, Melbourne, Australia; 9 University College London, London, UK; 10 Movember Foundation, Melbourne, Australia

**Keywords:** Prostate cancer, Health-related quality of life, Patient-reported outcomes, Registry

## Abstract

**Introduction:**

Similar Patient-Reported Outcomes (PROs) at diagnosis for localized prostate cancer among countries may indicate that different treatments are recommended to the same profile of patients, regardless the context characteristics (health systems, medical schools, culture, preferences…). The aim of this study was to assess such comparison.

**Methods:**

We analyzed the EPIC-26 results before the primary treatment of men diagnosed of localized prostate cancer from January 2017 onwards (revised data available up to September 2019), from a multicenter prospective international cohort including seven regions: Australia/New Zealand, Canada, Central Europe (Austria / Czech Republic / Germany), United Kingdom, Italy, Spain, and the United States. The EPIC-26 domain scores and pattern of three selected items were compared across regions (with Central Europe as reference). All comparisons were made stratifying by treatment: radical prostatectomy, external radiotherapy, brachytherapy, and active surveillance.

**Results:**

The sample included a total of 13,483 men with clinically localized or locally advanced prostate cancer. PROs showed different domain patterns before treatment across countries. The sexual domain was the most impaired, and the one with the highest dispersion within countries and with the greatest medians’ differences across countries. The urinary incontinence domain, together with the bowel and hormonal domains, presented the highest scores (better outcomes) for all treatment groups, and homogeneity across regions.

**Conclusions:**

Patients with localized or locally advanced prostate cancer undergoing radical prostatectomy, EBRT, brachytherapy, or active surveillance presented mainly negligible or small differences in the EPIC-26 domains before treatment across countries.

The results on urinary incontinence or bowel domains, in which almost all patients presented the best possible score, may downplay the baseline data role for evaluating treatments’ effects. However, the heterogeneity within countries and the magnitude of the differences found across countries in other domains, especially sexual, support the need of implementing the PRO measurement from diagnosis.

**Supplementary Information:**

The online version contains supplementary material available at 10.1186/s12894-023-01344-0.

## Background

Globally, prostate cancer is the first most commonly diagnosed cancer and the fifth leading cause of cancer death in men, with an estimated 1,414,259 new cases and 375,304 deaths in 2020 [1]. However, these figures substantially vary across countries. As the most incident cancer in men both in Europe and USA, it is estimated that in 2020 prostate cancer accounted for 23.2% of all new cancer cases diagnosed in men in Europe (excluding non-melanoma skin cancers) [2]; and it would represent 14.7% of all new cancer cases in the USA in 2023 [3]. Survival also varies across countries: 92% of men are alive at 5 years after diagnosis in high-income countries [4], compared to less than 60% in low- and middle-income countries [5]. The reason for variations in incidence may be related to the degree of economic development, social and lifestyle factors [1], genetic susceptibility, the availability and access to medical care [6], diagnostic and screening practices, and/or disease awareness [7]. Potential explanations for geographic variation in survival include access to care, health insurance status, income, and quality of care as well as possible lead time bias attributable to early diagnosis of asymptomatic disease [8–11].

At country level, factors such as social or health systems’ characteristics can also be determinants of prostate cancer patient-reported outcomes (PROs). PROs have been regarded as primary endpoints since the early 2000s due to the long-term survival in the majority of prostate cancer patients, especially those with localized diagnosis, and the focus on treatment side effects. In 2018, a systematic review [12] characterized treatment impact patterns as consistent with the ProtecT randomized clinical trial [13] and also with the long-term results of observational studies [14, 15]. All of these studies provide consistent scientific evidence on the deterioration after treatment, but they don’t mention whether patients have similar reported pretreatment statuses, regardless of country of diagnosis.

The available information on inter-country differences in patients’ health status at diagnosis of localized prostate cancer is limited. Pre-treatment differences in sexual function and bother between Japanese and American men have been reported [16]. Also, differences among patients diagnosed in the USA, Norway and Spain were observed [17] in pre-treatment medical and socio-demographic variables, as well as in urinary, sexual, and bowel health status.

These geographic differences in symptoms reported at diagnosis may disappear when comparing PROs at baseline stratifying by primary treatment, considering that the selection of one treatment or another is probably based on their reported symptoms together with clinical characteristics.

To test this hypothesis, our aim was to compare PROs before treatment for localized prostate cancer among countries, which may indicate that different treatments are recommended to the same profile of patients regardless the context characteristics (health systems, medical schools, culture, preferences…). Otherwise, the identification of differences in PROs before treatment among countries should be taken into account when assessing the treatments’ effectiveness.

The True North Registry (TNGR) [18] is a large-scale, multicenter-multicountry registry of the Movember Foundation that intends to record patterns of prostate cancer presentation, management, and outcomes, allowing to measure variation and facilitating any indicated healthcare quality improvement efforts. The participating sites contributing patient information are from Australia, Canada, Austria, Czech Republic, Germany, Italy, New Zealand, Spain, United Kingdom (with sites from England, Northern Ireland, Scotland, and Wales) and the United States of America. To avoid the identification of the single provider from Austria and Czech Republic, they were pooled with data from Germany, and Central Europe was the label chosen to define the region.

## Methods

Broadly, the research strategy involves systematically measuring clinical data and PROs of patients treated at one of the participating sites and who fulfill TNGRs inclusion (any T, any N and M0) and exclusion criteria (age < 18 years at diagnosis, acute or chronic conditions that could limit the ability of the patient to participate, inability to receive information about the study in a language they understand, and refusal of participation). Patients had to give written informed consent to participate in all countries except for the centers in Australia, New Zealand, and Canada that use an opt-out-approach for either clinical data and/or PROs at some of their sites (see further information in the Declarations section). Ethical approval was first given by the Monash University’s ethical committee and consecutively at each participating institution within the True North Global Registry. The study was conducted in accordance with the ethical standards of the institutional research committees and the 2000 revision of the Declaration of Helsinki. Organizations contributing data to this registry varied from large tertiary institutions to small public and private ones.

Data is safely uploaded twice a year in order to update the global Registry, following a standardized protocol specifically developed for this international initiative. This protocol includes a data dictionary of all variables recommended at the International Consortium for Health Outcomes Measurement (ICHOM)’s Localized Prostate Cancer Standard Set of outcomes [19].

For the present objective, a sample of patients from TNGR was selected using the following criteria: individuals diagnosed from January 2017 onward (prospective inclusion in the registry), who underwent one of the most prevalent primary management options (i.e., radical prostatectomy, external beam radiation therapy (EBRT), brachytherapy, or active surveillance), and who had available data on PROs prior to primary treatment. This selection was made using the TNGR database available in September 2019, which had been cleaned and reviewed to ensure that an unambiguous primary treatment was ascertainable.

### Clinical variables

Tumor risk class was grouped as “low risk”, “intermediate risk”, “high risk”, “very high risk”, and “regional” according to the National Comprehensive Cancer Network risk classification [20], using diagnosis biopsy Prostate-Specific Antigen (PSA), tumor size, and Gleason from grade 1 (Gleason score ≤ 6) to grade 5 (Gleason score 9–10) [21]. Comorbidities were recorded according to the Self-Administered Comorbidity Questionnaire [22] and categorized into “0”, “1”, and “2 or more”.

### Patient-reported outcomes

Before starting their primary treatment, and no later than 90 days from the diagnosis biopsy, patients responded (in different formats according to each participating site’s logistics) to their country’s version of the abbreviated form of the Expanded Prostate cancer Index Composite (the EPIC-26) [23]. This prostate cancer-specific PRO questionnaire has 26 items measuring 5 symptom domains (urinary incontinence, urinary irritative/obstructive, sexual, bowel, and vitality/hormonal). Response options for each EPIC item are 4- or 5-level Likert scales. Scores are transformed linearly to a scale from 0 to 100, where higher values indicate better outcomes.

The EPIC has been validated for its use in the majority of the countries included in this study (details and references included in the supplementary material).

### Analytical strategy

Summary statistics of patients’ characteristics and primary treatment were reported per country, and the differences were tested using Chi squared test for categorical variables or one-way analysis of variance (ANOVA) for continuous variables.

Boxplots for the EPIC-26 domain scores were constructed per country/region and evaluated with the Kruskal–Wallis test to explore differences in patients’ pre-interventional status, with Central Europe as reference.

The distribution of responses for selected items was also plotted, and frequencies were compared with chi-squared tests across countries. The selection of items was made by clinician consensus, considering those that would better describe a patient’s profile before the impact of treatments: *Which of the following best describes your urinary control during the last 4 weeks?; Overall, how would you rate your ability to function sexually during the last 4 weeks?; and How big a problem, if any, has urgency to have a bowel movement been for you?*

Statistical Analyses were conducted in SAS version 9.4 (SAS Institute, Cary, NC). *P* Values < 0.05 were considered statistically significant.

All comparisons were made stratifying by treatment.

## Results

The sample included a total of 13,483 men with clinically localized or locally advanced prostate cancer from Australia/New Zealand (*n* = 688), Canada (*n* = 1,640), Central Europe (*n* = 8,398), Italy (*n* = 477), Spain (*n* = 165), United Kingdom (*n* = 202), and the United States of America (USA, *n* = 1,913). This final sample is the result after filtering for those men diagnosed between 2017 and 2019, for whom pre-interventional data on PROs was available, and who after underwent one of the treatment options of interest (flowchart in supplementary material).

Table 1 shows clinical patient characteristics per country before treatment. Statistically significant differences were observed in the distribution of all characteristics per country. Mean (SD) patient age at recruitment ranged from 62.9 (7.2) in the USA to 67.0 (7.2) in the UK. Percentage of patients presenting PSA = 10 ranged from 84.4% in Spain to 68.6% in Central Europe, and patients with Gleason score in Grade 1 ranged from 52.4% in Spain to 21.8% in Central Europe, while patients at high risk ranged from 12.4% in Australia/New Zealand to 28.2% in the UK. Patients with no comorbidities represented 72.9% of the Canada sample, but only 31.5% of the Spain sample. The proportions of patients in each primary treatment group were heterogeneous across countries, but radical prostatectomy was the most frequent in all countries (from 92.3% in Australia/New Zealand, to 35.6% in the UK); and brachytherapy the least applied in general (from 24.8% in Spain, to 0% in Italy).

**Table 1:** Characteristics of patients and primary treatment by Country

	Australia/ New Zealand * (n* =688)	Canada (*n* =1640)	Central Europe^a^ (*n* =8398)	UK (*n* =202)	Italy (*n*=477)	Spain (*n* =165)	USA (*n* =1913)	*P Value*
**Age**								.
Mean (SD)	65.0 (6.50)	64.3 (7.59)	65.3 (7.27)	67.0 (7.18)	65.4 (6.39)	64.5 (7.88)	62.9 (7.19)	. <.0001
**PSA**								.
PSA ≤ 10	546 (81.5%)	1080 (72.1%)	5751 (68.6%)	90 (72.6%)	358 (75.2%)	124 (84.4%)	1431 (82.1%)	<.0001
PSA > 10	124 (18.5%)	417 (27.9%)	2637 (31.4%)	34 (27.4%)	118 (24.8%)	23 (15.6%)	315 (17.9%)	.
* Missing*	*18*	*143*	*10*	*78*	*1*	*18*	*167*	.
**Gleason**								.
Grade 1	301 (43.8%)	458 (28.1%)	1829 (21.8%)	42 (21.0%)	185 (38.9%)	86 (52.4%)	502 (27.8%)	<.0001
Grade 2	229 (33.3%)	701 (43.0%)	3079 (36.7%)	93 (46.5%)	120 (25.3%)	46 (28.0%)	680 (37.7%)	.
Grade 3	83 (12.1%)	250 (15.3%)	1609 (19.2%)	44 (22.0%)	72 (15.2%)	15 (9.1%)	326 (18.1%)	.
Grade 4	33 (4.8%)	87 (5.3%)	1199 (14.3%)	15 (7.5%)	72 (15.2%)	8 (4.9%)	166 (9.2%)	.
Grade 5	42 (6.1%)	133 (8.2%)	681 (8.1%)	6 (3.0%)	26 (5.5%)	9 (5.5%)	129 (7.2%)	.
* Missing*		*11*	*1*	*2*	*2*	*1*	*110*	*.*
**Tumor Risk**								.
Low Risk	234 (34.0%)	320 (19.5%)	1289 (15.3%)	9 (4.5%)	139 (29.1%)	57 (34.5%)	412 (21.5%)	<.0001
Intermediate Risk	340 (49.4%)	936 (57.1%)	4628 (55.1%)	97 (48.0%)	201 (42.1%)	71 (43.0%)	1035 (54.1%)	.
High Risk	85 (12.4%)	294 (17.9%)	2309 (27.5%)	57 (28.2%)	106 (22.2%)	24 (14.5%)	347 (18.1%)	.
Very High Risk	3 (0.4%)	26 (1.6%)	85 (1.0%)	13 (6.4%)	0 (0.0%)	5 (3.0%)	0 (0.0%)	.
Regional	8 (1.2%)	10 (0.6%)	84 (1.0%)	1 (0.5%)	29 (6.1%)	0 (0.0%)	6 (0.3%)	.
Unknown	18 (2.6%)	54 (3.3%)	3 (0.0%)	25 (12.4%)	2 (0.4%)	8 (4.8%)	113 (5.9%)	.
**Comorbidities**								.
0 comorbidities	225 (37.9%)	686 (72.9%)	5382 (72.3%)	67 (33.2%)	184 (41.3%)	52 (31.5%)	1286 (69.3%)	<.0001
1 comorbidity	208 (35.0%)	223 (23.7%)	1396 (18.8%)	71 (35.1%)	205 (46.0%)	50 (30.3%)	263 (14.2%)	.
2+ comorbidities	161 (27.1%)	32 (3.4%)	666 (8.9%)	64 (31.7%)	57 (12.8%)	63 (38.2%)	307 (16.5%)	.
* Missing*	*94*	*699*	*954*		*31*		*57*	.
**Primary Treatment**								
* Radical Prostatectomy *	*340 (49.4%)*	*794 (48.4%)*	*7754 (92.3%)*	*72 (35.6%)*	*414 (86.8%)*	*77 (46.7%)*	*1643 (85.9%)*	
* External Beam Radiotherapy *	*104 (15.1%)*	*354 (21.6%)*	*439 (5.2%)*	*53 (26.3%)*	*24 (5.0%)*	*47 (28.5%)*	*16 (0.8%)*	
* Brachytherapy *	*22 (3.2%)*	*83 (5.1%)*	*89 (1,1%)*	*30 (14.8%)*	*0 (0.0%)*	*41 (24.8%)*	*6 (0.3%)*	
* Active Surveillance *	*222 (32.2%)*	*409 (24.9%)*	*116 (1.4%)*	*47 (23.3%)*	*39 (8.2%)*	*0 (0.0%)*	*248 (13.0%)*	

^a^Central Europe includes sites from Austria, Czech Republic and Germany

**Fig. 1:**
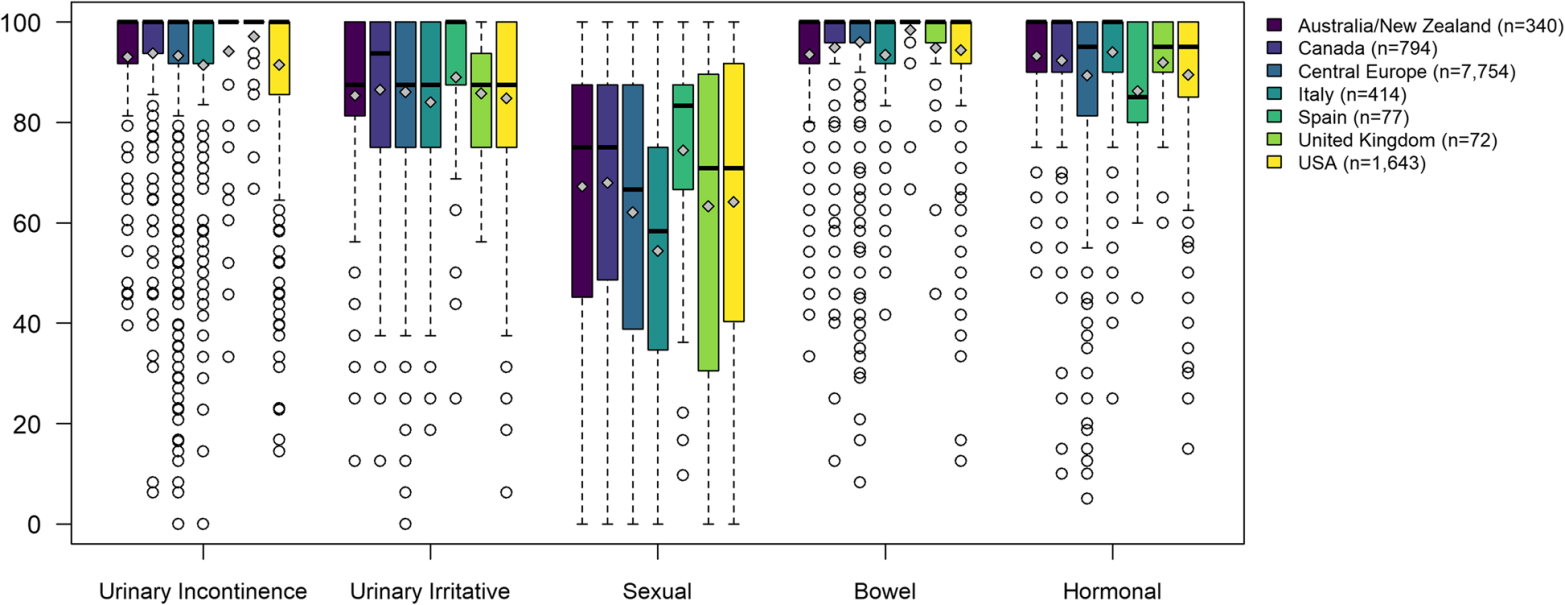
EPIC-26 domain scores o f patients who underwent Radical Prostatectomy. Differences across countries were statistically significant in all EPIC-26 scores (*p* < 0.001). Legend: The upper and lower whiskers represent scores outside the middle 50%, but within 1.5 times the interquartile range above the upper quartile and below the lower quartile (Q1—1.5 * IQR or Q3 + 1.5 * IQR)

EPIC-26 domain scores prior to *radical prostatectomy* are shown in Fig. 1, represented by boxplots where the colored boxes show where the middle half of patients are (from percentile 25 to percentile 75). Differences across countries were statistically significant in all domains, with Central Europe as reference. For Urinary Incontinence this half of the patients presented scores between 90 and 100, mostly, and the median (shown by the bold line in the box) was 100 in all countries (maximum possible score in the EPIC domains). For the smallest samples, UK and Spain (*n* = 72 and 77, respectively), almost all patients presented this maximum score. Very similar patterns were shown in the Bowel domain, with medians equal to 100 in all countries; and Hormonal domain, where three regions presented a median of 100 (Australia/New Zealand, Canada, and Italy), and other three a median of 95.0. Urinary Irritative/Obstructive scores were very similar in Australia/New Zealand (median [interquartile range, IQR] of 87.5 [81.3–100]), and in Central Europe, Italy, United Kingdom, and the USA (87.5 [75.0–100] in all three countries). Only Canada (93.8 [75.0–100]) and Spain (100 [87.5–100]) presented some distribution differences in this domain. Sexual scores presented a wider distribution in all regions, with medians ranging from 58.3 [34.7–75.0] in Italy, to 83.3 [66.7–87.5] in Spain.

**Fig. 2:**
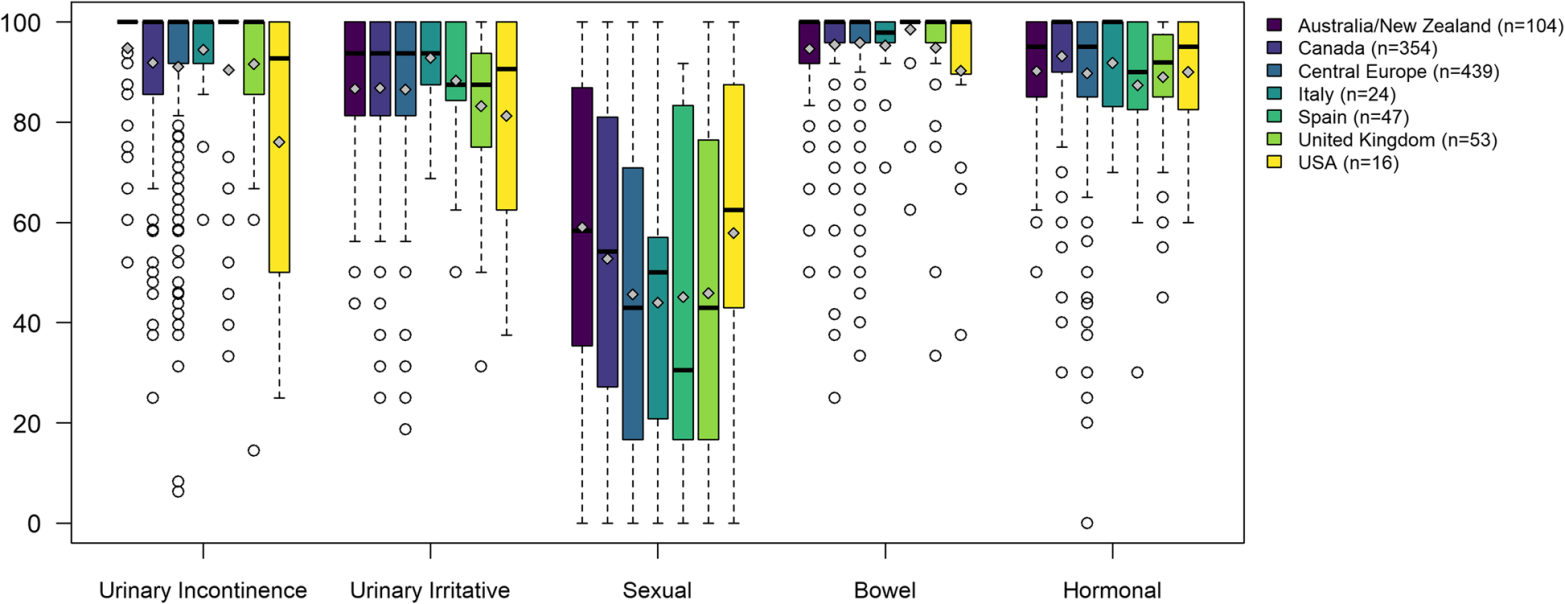
EPIC-26 domain scores of patients who underwent ERBT. Differences across countries were statistically significant in the majority of EPIC-26 scores (*p* < 0.05), with the exception of urinary irritative/obstructive (*p* = 0.153). Legend: The upper and lower whiskers represent scores outside the middle 50%, but within 1.5 times the interquartile range above the upper quartile and below the lower quartile (Q1—1.5 * IQR or Q3 + 1.5 * IQR)

Figure 2 shows the EPIC-26 scores’ distribution of patients before undergoing EBRT, with statistically significant differences among regions in all domains, except for the Urinary Irritative/Obstructive. The median of the Urinary Incontinence score was 100 in all regions except in the USA (92.8 [50.0–100]). The IQR of Bowel score in most regions was 95.8–100, with only some outliers located outside the whiskers of the boxplots. Similarly, more than half of the patients presented Hormonal domain scores above 80.0. The Urinary Irritative/Obstructive medians ranged from 87.5 in Spain and UK to 93.8 in Australia/New Zealand, Canada, Central Europe, and Italy. The Sexual domain presented greater heterogeneity, with medians and IQR ranging from 30.5 [16.7–83.3] in Spain to 62.5 [43.0–87.5] in the USA).

**Fig. 3:**
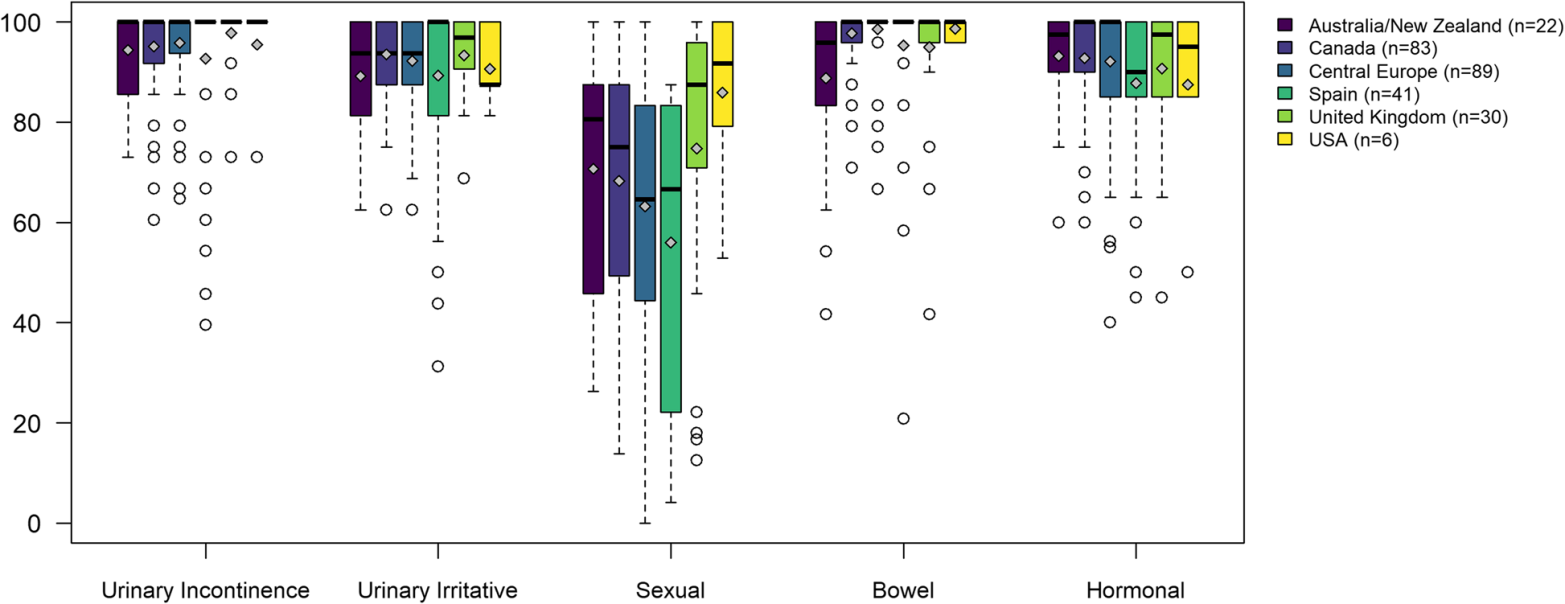
EPIC-26 domain scores of patients who underwent Brachytherapy (Italy did not have patients in this treatment group). Differences across countries were statistically significant only in the sexual (*p* = 0.006) and bowel (*p* = 0.0009) scores of EPIC-26. Legend: The upper and lower whiskers represent scores outside the middle 50%, but within 1.5 times the interquartile range above the upper quartile and below the lower quartile (Q1—1.5 * IQR or Q3 + 1.5 * IQR)

The EPIC-26 scores of patients before undergoing Brachytherapy as monotherapy only presented statistically significant differences among regions on the Sexual and Bowel domains (Fig. 3). Sexual medians and IQR ranged from 66.7 [22.2–83.3] in Spain to 91.7 [79.2–100] in the USA.

**Fig. 4:**
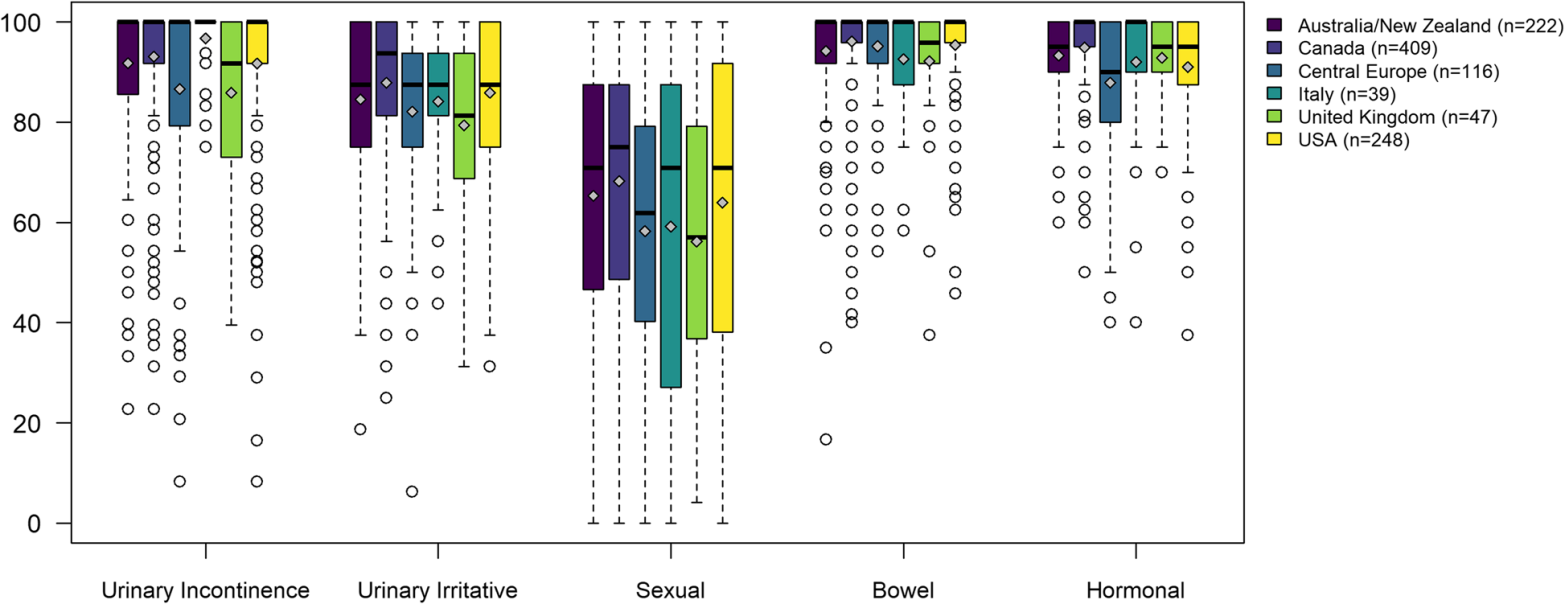
EPIC-26 domain scores of patients who underwent Active Surveillance (Spain did not have patients in this treatment group). Differences across countries were statistically significant in all EPIC-26 scores (*p* < 0.001). Legend: The upper and lower whiskers represent scores outside the middle 50%, but within 1.5 times the interquartile range above the upper quartile and below the lower quartile (Q1—1.5 * IQR or Q3 + 1.5 * IQR)

The EPIC-26 domain scores of patients who were on Active Surveillance (Fig. 4) show that differences across countries were statistically significant in all domains. The medians for Urinary Incontinence and Bowel were equal to 100 in all countries, except for UK (91.8 and 95.8, respectively). Hormonal scores presented medians of 100 in two countries (Canada and Italy), of 95 in three regions (Australia/New Zealand, UK and the USA), and of 90.0 in Central Europe. Medians for the Urinary Irritative/Obstructive domain were between 81.5 in UK, and 93.8 in Canada. The same countries presented the lowest and the highest medians for the Sexual domain, 57.0 and 75.0 respectively.

See the Supplementary material for the distribution of all EPIC-26 domain scores and items’ responses before treatment, with and without stratification per country and per treatment group.

**Fig. 5:**
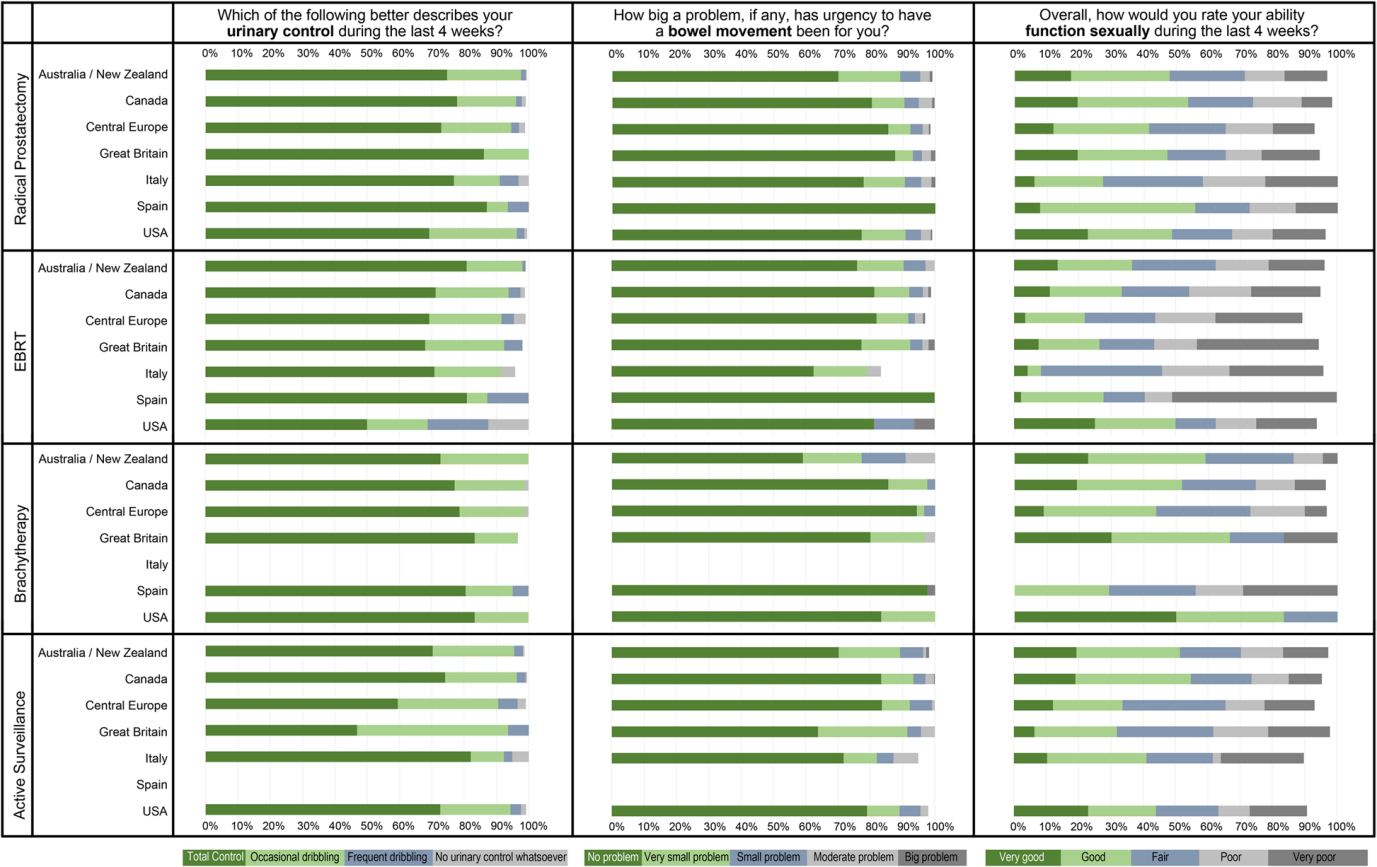
Distribution of responses to selected items across countries, stratified by treatment. All comparisons across countries were statistically significant (*p* < 0.05; chi-square test), except for the item “*Which of the following best describes your urinary control during the last 4 weeks?*” in the brachytherapy group (*p* = 0.48)

Figure 5 shows the distribution of responses to three selected items. Comparisons across countries were all statistically significant, except for the item *“Which of the following best describes your urinary control during the last 4 weeks?”* in the brachytherapy group. Among patients who later underwent radical prostatectomy, the UK sample presented the most favorable responses before treatment regarding *urinary control* (100% total control or occasional dribbling), while Italy showed the worst (8.9% reporting frequent dribbling or no urinary control whatsoever). *Urgency to have a bowel movement* was reported not to be a problem for all the Spanish patients who underwent radical prostatectomy, but was a moderate or big problem for up to 5% in UK or Canada. The majority of Spanish patients (55.9%) rated their *ability to function sexually* as good or very good, while only 27.3% of Italians did so.

Among patients who later underwent EBRT, Australia/New Zealand presented the best pattern of responses regarding *urinary control* (99% total control or occasional dribbling), while the USA showed the worst (31.3% reporting frequent dribbling or no urinary control whatsoever). *Urgency to have a bowel movement* was reported not to be a problem for the Spanish patients; but to be a moderate or big problem for almost 20% in the USA. The majority of patients in the USA (53.4%) rated their *ability to function sexually* as good or very good, while only 8.6% of Italians did so.

All patients that were later treated with brachytherapy in Australia/New Zealand, UK, and the USA reported total *urinary control* or occasional dribbling, while Spain showed worse results (4.9% reporting frequent dribbling or no urinary control whatsoever). *Urgency to have a bowel movement* was reported not to be a problem for more than 80% of patients who were going to be treated with brachytherapy; except for Australia/New Zealand (only 59% reported no problem). The large majority of patients in this group from the USA (83.3%) rated their *ability to function sexually* as good or very good, while in Spain only 26.3% of reported good sexual function (none reported very good).

The pattern of responses in those patients who enter on active surveillance, showed that more than 90% reported total *urinary control* or occasional dribbling, regardless of the country. Less than 5% reported *urgency to have a bowel movement* as a moderate or big problem in all countries, with a slightly higher percentage in Italy (8.1%). Between 50 and 60% of patients from Australia/New Zealand and Canada rated their *ability to function sexually* as good or very good, and between 32.6% and 48.4% in UK, Central Europe, Italy, and the USA.

## Discussion

After exploring pre-interventional status with PROs of men diagnosed with localized or locally advanced prostate cancer, findings showed different domain patterns across countries, even after stratifying by the primary selected treatment. Globally, the sexual domain was the most impaired in patients diagnosed with this pathology, and the one with the highest dispersion within countries and with the greatest medians’ differences across countries (with statistically significant differences in all treatment groups). Part of these differences may be explained by differences on age mean across countries in some treatment groups. For example, in radical prostatectomy, Spain presented the best score (74.4) in the sexual domain and a mean age of 59.3; while Australia showed a worse score (67.1) and a mean age of 64.5 years old. The urinary incontinence domain, together with the bowel and hormonal domains, presented the highest scores (better outcomes) for all treatment groups, and homogeneity across regions.

Beyond statistical differences, which are affected by the sample size of the compared groups, it is necessary to consider the magnitude of the differences between countries translating them into effect size coefficients (mean difference/pooled standard deviation, SD), and which values have been defined as small (0.2 SD), moderate (0.5 SD), and large (0.8 SD) [24].

Before radical prostatectomy, the magnitude of the differences across countries was small for almost all EPIC-26 domains. The greatest differences (with Central Europe as references) were found in incontinence with UK (0.27 SD); in irritative/obstructive and sexual domains with Spain (0.19 and 0.43 SD); and in bowel and hormonal domains with Italy (0.27 and 0.33 SD).

In the same line, differences between the EPIC-26 scores of patients that later started EBRT in Central Europe compared with those in other countries were negligible, except for the USA: with largely worse results in incontinence (0.86 SD), and moderately worse in bowel (0.56 SD); but better in the sexual domain (0.41 SD). Italy and Australia/New Zealand presented better results than Central Europe in irritative/obstructive and sexual scores respectively with effect size around 0.4 SD.

In contrast, among the patients undergoing brachytherapy as monotherapy, patients from the USA reported better sexual results (0.88 SD) than Central Europe. The comparison of EPIC-26 in this treatment group between Central Europe and the rest of the regions only demonstrated differences of small-moderate magnitude. The greatest were the better and worse results presented by UK for sexual (0.43 SD), and bowel (0.44 SD) domains, respectively.

Finally, although all EPIC-26 domains’ scores of patients under active surveillance presented statistically significant differences across countries, the magnitude was moderate-large only in the hormonal domain for Canada (0.71) and Australia (0.50 SD), and in the incontinence domain for Italy (0.55 SD).

In summary, most of the differences found across countries were negligible or of small magnitude, with very few moderate and only two large ones. However, the latter, both in the USA (incontinence in the EBRT group and sexual in brachytherapy as monotherapy), are not valuable due to the very small sample size in these treatment groups. This is consistent with the previously found moderate differences pre-treatment between the USA, Spain and Norway in all EPIC domains [17]. Although there is extensive evidence that results of generic PROs differ according to type of region (urban/rural), socioeconomic status of individuals, care experiences related to institutions [25] or clinicians, our results indicate that these differences may be less relevant for prostate cancer-specific PROs. In the case of EPIC-26, small differences before treatment could be explained because it was specifically designed to measure treatment side effects, which are not present at diagnosis.

The pattern of responses to the three selected items differed among countries with the exception of the item regarding ‘urinary control’ in the brachytherapy group, with almost all patients in all countries reporting good control. On one hand, more than 50% of patients in all countries reported good urinary control and no problem with urgency to have bowel movement, regardless of the treatment indicated. On the other hand, at least 50% of men reported poor overall sexual function ability in all groups (irrespective of treatment group or country). Similar differences in the EPIC items, but with greater magnitude, were found between the USA and Japan (52% vs 96%) [16].

Geographic differences in the EPIC-26 findings could reflect clinical heterogeneity among patients (for instance, patients at high risk ranged from 12.4% in Australia/New Zealand to 28.2% in the UK), and macro distinctions across participating sites, such as differences in health system characteristics. In any case, they confirm the importance of evaluating pre-interventional status before primary treatment, and to evaluate change after it. In research, the pre-treatment assessment is essential for measuring the real impact of the intervention, without the noise of potential confounders, and in clinical practice it is crucial for an adequate treatment-decision making, and a better management of adverse effects.

### Limitations

The main limitation of this study is that the True North Global Registry design does not assure the data to be representative of the participating countries: despite the large number of patients included as an international initiative, it may reflect the case mix of patients at the participating institutions, which can vary due to the nature of their clinical specialization. In fact, data from the National Cancer Database from USA, show that around 40% of men undergo prostatectomy and a similar percentage radiation [26]; far away from the representation of some countries in this study (radical prostatectomy 92%, 87% and 86% in Central Europe, Italy and USA). Furthermore, the observed differences in the EPIC-26 scores may reflect selection bias. Even though patients were invited to participate in the study regardless of the EPIC-26 results, differences on the scores may be explained by baseline characteristics (such as age, with a mean of 62.9 (7.19) in USA and of 67 (7.18) in UK). Our results should be interpreted with caution, as patients diagnosed with tumors that are clinically localized and locally advanced have been analyzed together, but they do not present the same profile prior to treatment (see stratified information in supplementary material). Differences have been shown in irritative/obstructive (*p* < 0.001) and sexual (*p* < 0.001) domains. For example, a mean score of 85.7 for clinically localized tumor, and of 81.4 for locally advanced. Another limitation is the variability in the administration of EPIC-26 among countries, which is mitigated by the fact that all participating sites adhere to a common study protocol, which follows the ICHOM standard set of outcomes for localized prostate cancer [26]. Finally, although the EPIC-26 has been widely used (and previously validated) in all the countries included in the study, some of the identified differences among them could be due to a ‘differential item functioning’ across country versions that has not been evaluated yet.

## Conclusions

Patients with localized or locally advanced prostate cancer undergoing radical prostatectomy, EBRT, brachytherapy, or active surveillance presented mainly negligible or small differences in the EPIC-26 domains across countries (only the USA presented large worse results in incontinence and sexual domains at the EBRT and brachytherapy groups, respectively).

The results on urinary incontinence or bowel domains, in which almost all patients presented the best possible score, may downplay the baseline data role for evaluating treatments’ effects. However, the heterogeneity within countries and the magnitude of the differences found across countries in other domains, especially sexual, support the need of implementing the PRO measurement from diagnosis.

For this reason, our findings support that further analyses on the impact of treatments among countries in the True North Global Registry will account for pre-interventional status reported by patients.

### Supplementary Information

**Additional file 1.**

## Data Availability

The datasets generated during and/or analyzed during the current study are not publicly available yet. Data requests can be addressed to the Project Coordinating Centre at PCOCRVInfo@mednet.ucla.edu.

## Abbreviations

PROs: Patient-Reported Outcomes
TNGR: True North Global Registry
ICHOM: International Consortium for Health Outcomes Measurement
EBRT: External Beam Radiation Therapy
PSA: Prostate-Specific Antigen
EPIC: Expanded Prostate cancer Index Composite
ANOVA: ANalysis Of VAriance
IQR: InterQuartile Range
SD: Standard Deviation
