# Supplementary material for: Patient-reported outcomes before treatment for localized prostate cancer: are there differences among countries? Data from the True North Global Registry
Source: BMC Urol. 2023 Nov 3;23:178. doi: 10.1186/s12894-023-01344-0 (PMC10623840; doi:10.1186/s12894-023-01344-0)
Supplement: Supplementary file 1 — Additional file 1. [file 12894_2023_1344_MOESM1_ESM.pdf]

Validation studies of each EPIC-26 country version.

**United States of America**	**English version (EPIC-26)**	Szymanski KM, Wei JT, Dunn RL, Sanda MG. Development and validation of an abbreviated version of the expanded prostate cancer index composite instrument for measuring health-related quality of life among prostate cancer survivors. Urology. 2010 Nov;76(5):1245-50.
**Canada**		
**United Kingdom**		
**Australia**		
**New Zealand**		
**Canada**	**French version (EPIC-26)**	Vigneault E, Savard J, Savard MH, et al. Validation of the French-Canadian version of the Expanded Prostate Cancer Index Composite (EPIC) in a French-Canadian population. Journal de l'Association des urologues du Canada. 2017;11(12):404-410.
**Germany**	**German version (EPIC-26)**	Sibert NT, Dieng S, Oesterle A, et al. Psychometric validation of the German version of the EPIC-26 questionnaire for patients with localized and locally advanced prostate cancer. World J Urol. 2021 Jan;39(1):11-25.
**Austria**		
**Italy**	**Italian version (EPIC-26)**	Marzorati C, Monzani D, Mazzocco K, et al. Validation of the Italian version of the abbreviated expanded prostate Cancer index composite (EPIC-26) in men with prostate Cancer. Health Qual Life Outcomes. 2019 Aug 29;17(1):147.
**Spain**	**Spanish version (EPIC-50)**	Ferrer M, Garin O, Pera J, et al. [Evaluation of the quality of life of patients with localizad prostate cancer: validation of the Spanish version of the EPIC]. Med Clin (Barc). 2009 Feb 7;132(4):128-35.
**Czech Republic**	[not available]	

Flowchart of participants included in the study:

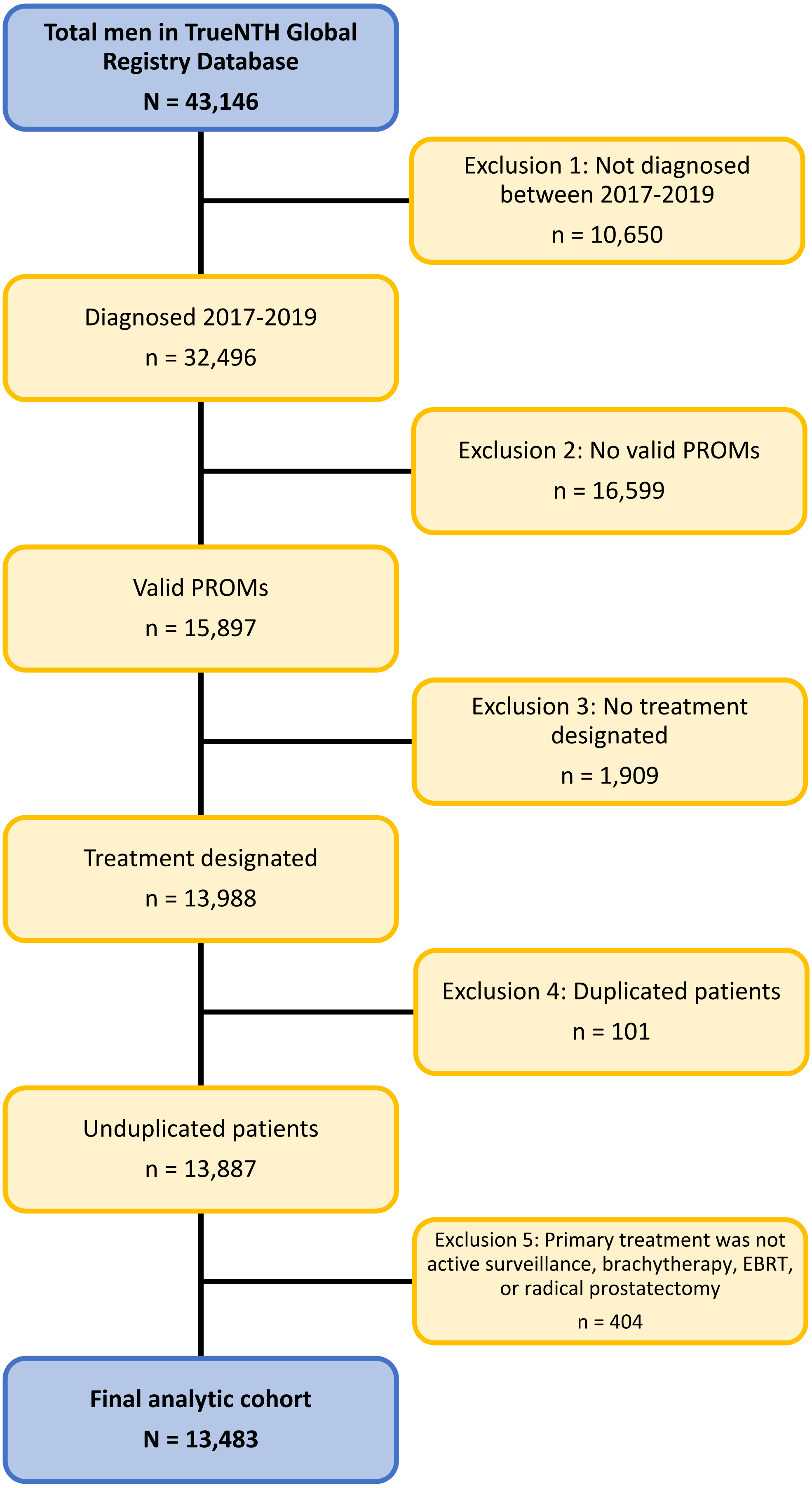

EPIC-26 Baseline (pre-treatment) Results

Statistic Variable or Category	TNGR Global (n=13483)	Australia/New Zealand (n=688)	Canada (n=1640)	Central Europe (n=8398)	United Kingdom (n=202)	Italy (n=477)	Spain (n=165)	USA (n=1913)	P Value
Urinary Incontinence									.
n	12855	663	1584	7919	196	472	165	1856	<.0001
Mean (SD)	92.8 (13.99)	92.9 (13.32)	93.3 (13.05)	93.1 (13.75)	93.1 (13.64)	92.0 (16.32)	92.7 (17.06)	91.3 (15.01)	.
Median (Q1-Q3)	100.0 (91.8 - 100.0)	100.0 (91.8 - 100.0)	100.0 (91.8 - 100.0)	100.0 (91.8 - 100.0)	100.0 (91.8 - 100.0)	100.0 (91.8 - 100.0)	100.0 (100.0 - 100.0)	100.0 (85.5 - 100.0)	.
Min, Max	0.0, 100.0	22.8, 100.0	6.3, 100.0	0.0, 100.0	14.5, 100.0	0.0, 100.0	33.3, 100.0	8.3, 100.0	.
Over the past 4 weeks, how often have you leaked urine?									.
More than once a day	515 (3.9%)	35 (5.1%)	71 (4.4%)	281 (3.4%)	9 (4.5%)	13 (2.7%)	3 (1.8%)	103 (5.4%)	<.0001
About once a day	456 (3.4%)	32 (4.7%)	50 (3.1%)	234 (2.8%)	9 (4.5%)	23 (4.8%)	7 (4.2%)	101 (5.3%)	.
More than once a week	392 (2.9%)	25 (3.7%)	50 (3.1%)	197 (2.4%)	5 (2.5%)	15 (3.2%)	12 (7.3%)	88 (4.6%)	.
About once a week	547 (4.1%)	37 (5.4%)	109 (6.7%)	254 (3.1%)	14 (6.9%)	21 (4.4%)	4 (2.4%)	108 (5.7%)	.
Rarely or never	11432 (85.7%)	552 (81.1%)	1345 (82.8%)	7328 (88.4%)	165 (81.7%)	404 (84.9%)	139 (84.2%)	1499 (78.9%)	.
Missing	141	7	15	104		1		14	.
Which of the following best describes your urinary control during the last 4 weeks?									.
No urinary control whatsoever	223 (1.7%)	2 (0.3%)	18 (1.1%)	167 (2.0%)	0 (0.0%)	16 (3.4%)	0 (0.0%)	20 (1.1%)	<.0001
Frequent dribbling	355 (2.7%)	12 (1.8%)	38 (2.3%)	212 (2.6%)	6 (3.0%)	25 (5.3%)	13 (7.9%)	49 (2.6%)	.
Occasional dribbling	2957 (22.1%)	158 (23.2%)	334 (20.5%)	1830 (22.0%)	49 (24.5%)	68 (14.3%)	14 (8.5%)	504 (26.5%)	.
Total control	9825 (73.5%)	510 (74.8%)	1237 (76.0%)	6098 (73.4%)	145 (72.5%)	367 (77.1%)	138 (83.6%)	1330 (69.9%)	.
Missing	123	6	13	91	2	1		10	.
How many pads or adult diapers per day did you usually use to control leakage during the last 4 weeks?									.
None	12969 (97.1%)	673 (98.5%)	1579 (98.6%)	8035 (96.4%)	195 (98.5%)	451 (94.7%)	165 (100.0%)	1871 (98.4%)	<.0001
1 pad per day	285 (2.1%)	9 (1.3%)	17 (1.1%)	227 (2.7%)	2 (1.0%)	11 (2.3%)	0 (0.0%)	19 (1.0%)	.
2 pads per day	64 (0.5%)	1 (0.1%)	3 (0.2%)	44 (0.5%)	0 (0.0%)	9 (1.9%)	0 (0.0%)	7 (0.4%)	.
3 or more pads per day	40 (0.3%)	0 (0.0%)	3 (0.2%)	27 (0.3%)	1 (0.5%)	5 (1.1%)	0 (0.0%)	4 (0.2%)	.
Missing	125	5	38	65	4	1		12	.

EPIC-26 Baseline (pre-treatment) Results

Statistic Variable or Category	TNGR Global (n=13483)	Australia/New Zealand (n=688)	Canada (n=1640)	Central Europe (n=8398)	United Kingdom (n=202)	Italy (n=477)	Spain (n=165)	USA (n=1913)	P Value
How big a problem, if any, has dripping or leaking urine been for you during the last 4 weeks?									.
No problem	10192 (78.0%)	513 (76.5%)	1261 (77.4%)	6376 (79.2%)	159 (78.7%)	369 (78.2%)	137 (83.0%)	1377 (73.8%)	<.0001
Very small problem	1915 (14.7%)	122 (18.2%)	273 (16.7%)	1084 (13.5%)	32 (15.8%)	65 (13.8%)	0 (0.0%)	339 (18.2%)	.
Small problem	603 (4.6%)	23 (3.4%)	69 (4.2%)	361 (4.5%)	6 (3.0%)	27 (5.7%)	12 (7.3%)	105 (5.6%)	.
Moderate problem	271 (2.1%)	12 (1.8%)	24 (1.5%)	174 (2.2%)	5 (2.5%)	8 (1.7%)	10 (6.1%)	38 (2.0%)	.
Big problem	79 (0.6%)	1 (0.1%)	3 (0.2%)	58 (0.7%)	0 (0.0%)	3 (0.6%)	6 (3.6%)	8 (0.4%)	.
Missing	423	17	10	345		5		46	.
Urinary Irritative/Obst.									.
n	12765	649	1625	7832	195	471	165	1828	<.0001
Mean (SD)	86.0 (15.51)	85.4 (15.41)	87.3 (14.52)	86.1 (15.59)	84.6 (14.49)	84.4 (17.30)	88.9 (15.77)	84.9 (15.56)	.
Median (Q1-Q3)	87.5 (75.0 - 100.0)	87.5 (81.3 - 100.0)	93.8 (81.3 - 100.0)	87.5 (75.0 - 100.0)	87.5 (75.0 - 100.0)	87.5 (75.0 - 100.0)	100.0 (87.5 - 100.0)	87.5 (75.0 - 100.0)	.
Min, Max	0.0, 100.0	12.5, 100.0	12.5, 100.0	0.0, 100.0	31.3, 100.0	18.8, 100.0	25.0, 100.0	6.3, 100.0	.
How big a problem, if any, has pain or burning on urination been for you during the last 4 weeks?									.
No problem	11299 (86.9%)	583 (87.1%)	1432 (87.7%)	6997 (87.3%)	175 (87.1%)	359 (76.2%)	139 (84.2%)	1614 (86.9%)	<.0001
Very small problem	1011 (7.8%)	58 (8.7%)	142 (8.7%)	586 (7.3%)	17 (8.5%)	74 (15.7%)	3 (1.8%)	131 (7.1%)	.
Small problem	385 (3.0%)	13 (1.9%)	31 (1.9%)	223 (2.8%)	4 (2.0%)	23 (4.9%)	17 (10.3%)	74 (4.0%)	.
Moderate problem	225 (1.7%)	13 (1.9%)	18 (1.1%)	141 (1.8%)	4 (2.0%)	13 (2.8%)	4 (2.4%)	32 (1.7%)	.
Big problem	89 (0.7%)	2 (0.3%)	10 (0.6%)	66 (0.8%)	1 (0.5%)	2 (0.4%)	2 (1.2%)	6 (0.3%)	.
Missing	474	19	7	385	1	6		56	.
How big a problem, if any, has bleeding with urination been for you during the last 4 weeks?									.
No problem	12176 (94.1%)	600 (89.3%)	1519 (93.1%)	7566 (95.2%)	183 (92.9%)	439 (93.2%)	164 (99.4%)	1705 (92.1%)	<.0001
Very small problem	456 (3.5%)	50 (7.4%)	83 (5.1%)	206 (2.6%)	7 (3.6%)	14 (3.0%)	0 (0.0%)	96 (5.2%)	.
Small problem	183 (1.4%)	18 (2.7%)	15 (0.9%)	101 (1.3%)	3 (1.5%)	11 (2.3%)	0 (0.0%)	35 (1.9%)	.
Moderate problem	95 (0.7%)	4 (0.6%)	15 (0.9%)	55 (0.7%)	4 (2.0%)	4 (0.8%)	0 (0.0%)	13 (0.7%)	.
Big problem	29 (0.2%)	0 (0.0%)	0 (0.0%)	22 (0.3%)	0 (0.0%)	3 (0.6%)	1 (0.6%)	3 (0.2%)	.

EPIC-26 Baseline (pre-treatment) Results

Statistic Variable or Category	TNGR Global (n=13483)	Australia/New Zealand (n=688)	Canada (n=1640)	Central Europe (n=8398)	United Kingdom (n=202)	Italy (n=477)	Spain (n=165)	USA (n=1913)	P Value
Missing	544	16	8	448	5	6		61	.
How big a problem, if any, has weak urine stream been for you during the last 4 weeks?									.
No problem	6361 (48.5%)	303 (45.0%)	845 (51.8%)	3874 (47.8%)	94 (47.2%)	240 (50.8%)	108 (65.5%)	897 (48.1%)	<.0001
Very small problem	3194 (24.4%)	190 (28.2%)	402 (24.7%)	2014 (24.8%)	50 (25.1%)	105 (22.2%)	2 (1.2%)	431 (23.1%)	.
Small problem	1930 (14.7%)	97 (14.4%)	232 (14.2%)	1193 (14.7%)	24 (12.1%)	66 (14.0%)	34 (20.6%)	284 (15.2%)	.
Moderate problem	1244 (9.5%)	69 (10.2%)	121 (7.4%)	770 (9.5%)	29 (14.6%)	51 (10.8%)	15 (9.1%)	189 (10.1%)	.
Big problem	384 (2.9%)	15 (2.2%)	30 (1.8%)	259 (3.2%)	2 (1.0%)	10 (2.1%)	6 (3.6%)	62 (3.3%)	.
Missing	370	14	10	288	3	5		50	.
How big a problem, if any, has need to urinate frequently during the day been for you during the last 4 weeks?									.
No problem	6043 (45.8%)	295 (44.2%)	795 (48.6%)	3792 (46.4%)	76 (37.8%)	207 (43.8%)	129 (78.2%)	749 (39.9%)	<.0001
Very small problem	2985 (22.6%)	167 (25.0%)	397 (24.3%)	1801 (22.1%)	50 (24.9%)	108 (22.8%)	1 (0.6%)	461 (24.6%)	.
Small problem	2108 (16.0%)	111 (16.6%)	231 (14.1%)	1296 (15.9%)	43 (21.4%)	74 (15.6%)	17 (10.3%)	336 (17.9%)	.
Moderate problem	1576 (12.0%)	81 (12.1%)	171 (10.5%)	959 (11.7%)	30 (14.9%)	66 (14.0%)	13 (7.9%)	256 (13.6%)	.
Big problem	471 (3.6%)	14 (2.1%)	42 (2.6%)	316 (3.9%)	2 (1.0%)	18 (3.8%)	5 (3.0%)	74 (3.9%)	.
Missing	300	20	4	234	1	4		37	.
Overall how big a problem has your urinary function been for you during the last 4 weeks?									.
No problem	7698 (58.2%)	354 (52.0%)	946 (58.1%)	4965 (60.7%)	96 (47.5%)	247 (51.9%)	99 (60.0%)	991 (52.1%)	<.0001
Very small problem	2780 (21.0%)	200 (29.4%)	366 (22.5%)	1579 (19.3%)	51 (25.2%)	106 (22.3%)	1 (0.6%)	477 (25.1%)	.
Small problem	1449 (10.9%)	69 (10.1%)	187 (11.5%)	819 (10.0%)	33 (16.3%)	62 (13.0%)	36 (21.8%)	243 (12.8%)	.
Moderate problem	1031 (7.8%)	53 (7.8%)	109 (6.7%)	625 (7.6%)	22 (10.9%)	54 (11.3%)	21 (12.7%)	147 (7.7%)	.
Big problem	276 (2.1%)	5 (0.7%)	21 (1.3%)	191 (2.3%)	0 (0.0%)	7 (1.5%)	8 (4.8%)	44 (2.3%)	.
Missing	249	7	11	219		1		11	.
Bowel									.
n	12850	675	1631	7849	195	463	165	1872	<.0001
Mean (SD)	95.5 (9.84)	93.8 (11.02)	95.5 (9.98)	96.0 (9.27)	94.2 (11.93)	93.4 (11.40)	97.7 (8.97)	94.5 (10.75)	.

EPIC-26 Baseline (pre-treatment) Results

Statistic Variable or Category	TNGR Global (n=13483)	Australia/New Zealand (n=688)	Canada (n=1640)	Central Europe (n=8398)	United Kingdom (n=202)	Italy (n=477)	Spain (n=165)	USA (n=1913)	P Value
Median (Q1-Q3)	100.0 (95.8 - 100.0)	100.0 (91.7 - 100.0)	100.0 (95.8 - 100.0)	100.0 (95.8 - 100.0)	100.0 (91.7 - 100.0)	100.0 (91.7 - 100.0)	100.0 (100.0 - 100.0)	100.0 (95.8 - 100.0)	.
Min, Max	8.3, 100.0	16.7, 100.0	12.5, 100.0	8.3, 100.0	33.3, 100.0	41.7, 100.0	20.8, 100.0	12.5, 100.0	.
How big a problem, if any, has urgency to have a bowel movement been for you?									.
No problem	11159 (83.8%)	486 (71.4%)	1338 (81.9%)	7166 (86.6%)	158 (78.2%)	365 (77.5%)	164 (99.4%)	1482 (78.3%)	<.0001
Very small problem	1225 (9.2%)	126 (18.5%)	169 (10.3%)	589 (7.1%)	30 (14.9%)	61 (13.0%)	0 (0.0%)	250 (13.2%)	.
Small problem	547 (4.1%)	47 (6.9%)	67 (4.1%)	308 (3.7%)	6 (3.0%)	23 (4.9%)	0 (0.0%)	96 (5.1%)	.
Moderate problem	308 (2.3%)	17 (2.5%)	50 (3.1%)	162 (2.0%)	6 (3.0%)	17 (3.6%)	0 (0.0%)	56 (3.0%)	.
Big problem	77 (0.6%)	5 (0.7%)	10 (0.6%)	46 (0.6%)	2 (1.0%)	5 (1.1%)	1 (0.6%)	8 (0.4%)	.
Missing	167	7	6	127		6		21	.
How big a problem, if any, has increased frequency of bowel movements been for you?									.
No problem	11185 (86.7%)	539 (80.1%)	1412 (86.7%)	6953 (88.1%)	164 (83.2%)	390 (84.2%)	161 (97.6%)	1566 (83.6%)	<.0001
Very small problem	1010 (7.8%)	81 (12.0%)	140 (8.6%)	545 (6.9%)	22 (11.2%)	40 (8.6%)	0 (0.0%)	182 (9.7%)	.
Small problem	431 (3.3%)	36 (5.3%)	43 (2.6%)	248 (3.1%)	4 (2.0%)	21 (4.5%)	1 (0.6%)	78 (4.2%)	.
Moderate problem	216 (1.7%)	12 (1.8%)	28 (1.7%)	114 (1.4%)	6 (3.0%)	12 (2.6%)	2 (1.2%)	42 (2.2%)	.
Big problem	52 (0.4%)	5 (0.7%)	6 (0.4%)	34 (0.4%)	1 (0.5%)	0 (0.0%)	1 (0.6%)	5 (0.3%)	.
Missing	589	15	11	504	5	14		40	.
How big a problem, if any, has losing control of your stools been for you?									.
No problem	12345 (96.0%)	625 (93.1%)	1564 (96.0%)	7611 (96.9%)	187 (93.0%)	439 (94.8%)	162 (98.2%)	1757 (93.9%)	<.0001
Very small problem	330 (2.6%)	31 (4.6%)	42 (2.6%)	164 (2.1%)	11 (5.5%)	14 (3.0%)	0 (0.0%)	68 (3.6%)	.
Small problem	113 (0.9%)	10 (1.5%)	13 (0.8%)	44 (0.6%)	2 (1.0%)	9 (1.9%)	0 (0.0%)	35 (1.9%)	.
Moderate problem	46 (0.4%)	3 (0.4%)	6 (0.4%)	24 (0.3%)	1 (0.5%)	1 (0.2%)	2 (1.2%)	9 (0.5%)	.
Big problem	22 (0.2%)	2 (0.3%)	5 (0.3%)	12 (0.2%)	0 (0.0%)	0 (0.0%)	1 (0.6%)	2 (0.1%)	.
Missing	627	17	10	543	1	14		42	.
How big a problem, if any, has bloody stools been for you?									.

EPIC-26 Baseline (pre-treatment) Results

Statistic Variable or Category	TNGR Global (n=13483)	Australia/New Zealand (n=688)	Canada (n=1640)	Central Europe (n=8398)	United Kingdom (n=202)	Italy (n=477)	Spain (n=165)	USA (n=1913)	P Value
No problem	12445 (96.7%)	645 (95.4%)	1561 (95.6%)	7654 (97.4%)	190 (97.9%)	443 (95.7%)	160 (97.0%)	1792 (95.6%)	0.0004
Very small problem	291 (2.3%)	25 (3.7%)	52 (3.2%)	139 (1.8%)	2 (1.0%)	11 (2.4%)	1 (0.6%)	61 (3.3%)	.
Small problem	95 (0.7%)	6 (0.9%)	14 (0.9%)	50 (0.6%)	1 (0.5%)	7 (1.5%)	4 (2.4%)	13 (0.7%)	.
Moderate problem	28 (0.2%)	0 (0.0%)	4 (0.2%)	14 (0.2%)	1 (0.5%)	2 (0.4%)	0 (0.0%)	7 (0.4%)	.
Big problem	6 (0.0%)	0 (0.0%)	2 (0.1%)	3 (0.0%)	0 (0.0%)	0 (0.0%)	0 (0.0%)	1 (0.1%)	.
Missing	618	12	7	538	8	14		39	.
How big a problem, if any, has abdominal/pelvic/rectal pain been for you?									.
No problem	10960 (85.0%)	540 (79.9%)	1400 (85.8%)	6772 (85.8%)	157 (79.7%)	366 (79.0%)	154 (93.3%)	1571 (84.1%)	<.0001
Very small problem	1127 (8.7%)	90 (13.3%)	155 (9.5%)	644 (8.2%)	18 (9.1%)	54 (11.7%)	0 (0.0%)	166 (8.9%)	.
Small problem	463 (3.6%)	29 (4.3%)	39 (2.4%)	281 (3.6%)	11 (5.6%)	26 (5.6%)	5 (3.0%)	72 (3.9%)	.
Moderate problem	268 (2.1%)	11 (1.6%)	30 (1.8%)	153 (1.9%)	9 (4.6%)	14 (3.0%)	5 (3.0%)	46 (2.5%)	.
Big problem	76 (0.6%)	6 (0.9%)	8 (0.5%)	42 (0.5%)	2 (1.0%)	3 (0.6%)	1 (0.6%)	14 (0.7%)	.
Missing	589	12	8	506	5	14		44	.
Overall, how big a problem have your bowel habits been for you during the last 4 weeks?									.
No problem	11200 (83.9%)	523 (77.6%)	1375 (84.1%)	7116 (85.7%)	166 (82.2%)	358 (75.4%)	155 (93.9%)	1507 (79.3%)	<.0001
Very small problem	1324 (9.9%)	105 (15.6%)	176 (10.8%)	697 (8.4%)	23 (11.4%)	55 (11.6%)	0 (0.0%)	268 (14.1%)	.
Small problem	472 (3.5%)	24 (3.6%)	46 (2.8%)	272 (3.3%)	5 (2.5%)	38 (8.0%)	3 (1.8%)	84 (4.4%)	.
Moderate problem	273 (2.0%)	18 (2.7%)	30 (1.8%)	157 (1.9%)	8 (4.0%)	20 (4.2%)	6 (3.6%)	34 (1.8%)	.
Big problem	82 (0.6%)	4 (0.6%)	7 (0.4%)	58 (0.7%)	0 (0.0%)	4 (0.8%)	1 (0.6%)	8 (0.4%)	.
Missing	132	14	6	98		2		12	.
Sexual									.
n	12575	662	1584	7684	196	470	165	1814	<.0001
Mean (SD)	62.0 (29.34)	65.5 (28.64)	64.8 (28.01)	61.2 (29.17)	58.8 (31.86)	54.3 (27.30)	61.5 (29.76)	64.2 (31.04)	.
Median (Q1-Q3)	66.7 (38.8 - 87.5)	70.8 (44.5 - 87.5)	70.8 (43.0 - 87.5)	65.3 (37.5 - 87.5)	66.7 (28.5 - 87.5)	58.3 (32.0 - 75.0)	75.0 (30.5 - 87.5)	70.8 (40.3 - 91.7)	.
Min, Max	0.0, 100.0	0.0, 100.0	0.0, 100.0	0.0, 100.0	0.0, 100.0	0.0, 100.0	0.0, 100.0	0.0, 100.0	.

EPIC-26 Baseline (pre-treatment) Results

Statistic Variable or Category	TNGR Global (n=13483)	Australia/New Zealand (n=688)	Canada (n=1640)	Central Europe (n=8398)	United Kingdom (n=202)	Italy (n=477)	Spain (n=165)	USA (n=1913)	P Value
How would you rate your ability to have an erection during the last 4 weeks?									.
Very poor to none	1834 (14.3%)	76 (11.3%)	181 (11.3%)	1119 (14.2%)	45 (22.5%)	91 (19.2%)	35 (21.2%)	287 (15.5%)	<.0001
Poor	1873 (14.6%)	98 (14.6%)	248 (15.5%)	1132 (14.4%)	28 (14.0%)	98 (20.7%)	17 (10.3%)	252 (13.6%)	.
Fair	3204 (24.9%)	151 (22.4%)	359 (22.4%)	2074 (26.3%)	45 (22.5%)	141 (29.7%)	25 (15.2%)	409 (22.0%)	.
Good	3844 (29.9%)	200 (29.7%)	553 (34.5%)	2381 (30.2%)	54 (27.0%)	107 (22.6%)	85 (51.5%)	464 (25.0%)	.
Very good	2101 (16.3%)	148 (22.0%)	261 (16.3%)	1181 (15.0%)	28 (14.0%)	37 (7.8%)	3 (1.8%)	443 (23.9%)	.
Missing	627	15	38	511	2	3		58	.
How would you rate your ability to reach orgasm (climax) during the last 4 weeks?									.
Very poor to none	1708 (13.5%)	79 (12.0%)	179 (11.2%)	1031 (13.3%)	41 (20.8%)	84 (17.9%)	37 (22.4%)	257 (14.2%)	<.0001
Poor	1552 (12.3%)	74 (11.2%)	197 (12.3%)	975 (12.6%)	24 (12.2%)	78 (16.6%)	11 (6.7%)	193 (10.6%)	.
Fair	2763 (21.9%)	139 (21.0%)	337 (21.1%)	1785 (23.1%)	43 (21.8%)	145 (30.9%)	13 (7.9%)	301 (16.6%)	.
Good	4209 (33.3%)	216 (32.7%)	569 (35.7%)	2639 (34.2%)	50 (25.4%)	120 (25.5%)	102 (61.8%)	513 (28.2%)	.
Very good	2397 (19.0%)	153 (23.1%)	314 (19.7%)	1294 (16.8%)	39 (19.8%)	43 (9.1%)	2 (1.2%)	552 (30.4%)	.
Missing	854	27	44	674	5	7		97	.
How would you describe the usual quality of your erections during the last 4 weeks?									.
None at all	1869 (14.7%)	74 (11.1%)	150 (9.4%)	1266 (16.2%)	32 (16.0%)	78 (16.5%)	33 (20.0%)	236 (12.8%)	<.0001
Not firm enough for any sexual activity	1655 (13.0%)	73 (10.9%)	230 (14.5%)	1015 (13.0%)	35 (17.5%)	74 (15.6%)	11 (6.7%)	217 (11.8%)	.
Firm enough for masturbation and foreplay	2360 (18.5%)	125 (18.7%)	310 (19.5%)	1487 (19.1%)	38 (19.0%)	72 (15.2%)	18 (10.9%)	310 (16.9%)	.
Firm enough for intercourse	6855 (53.8%)	397 (59.3%)	899 (56.6%)	4036 (51.7%)	95 (47.5%)	250 (52.7%)	103 (62.4%)	1075 (58.5%)	.
Missing	744	19	51	594	2	3		75	.
How would you describe the frequency of your erections during the last 4 weeks?									.
I never had an erection when I wanted one	1952 (15.7%)	85 (12.8%)	180 (11.4%)	1245 (16.4%)	40 (20.2%)	83 (17.7%)	47 (28.5%)	272 (15.0%)	<.0001

EPIC-26 Baseline (pre-treatment) Results

Statistic Variable or Category	TNGR Global (n=13483)	Australia/New Zealand (n=688)	Canada (n=1640)	Central Europe (n=8398)	United Kingdom (n=202)	Italy (n=477)	Spain (n=165)	USA (n=1913)	P Value
I had an erection less than half the time I wanted one	1741 (14.0%)	81 (12.2%)	218 (13.9%)	1132 (14.9%)	25 (12.6%)	71 (15.1%)	4 (2.4%)	210 (11.6%)	.
I had an erection about half the time I wanted one	1807 (14.5%)	77 (11.6%)	231 (14.7%)	1131 (14.9%)	20 (10.1%)	129 (27.4%)	12 (7.3%)	207 (11.4%)	.
I had an erection more than half the time I wanted one	2038 (16.3%)	143 (21.6%)	243 (15.4%)	1199 (15.8%)	28 (14.1%)	90 (19.1%)	8 (4.8%)	327 (18.0%)	.
I had an erection whenever I wanted one	4931 (39.5%)	277 (41.8%)	701 (44.6%)	2879 (38.0%)	85 (42.9%)	97 (20.6%)	94 (57.0%)	798 (44.0%)	.
Missing	1014	25	67	812	4	7		99	.
Overall, how would you rate your ability to function sexually during the last 4 weeks?									.
Very poor	1956 (15.4%)	95 (14.2%)	201 (12.7%)	1140 (14.7%)	47 (24.2%)	110 (23.3%)	46 (27.9%)	317 (17.4%)	<.0001
Poor	1937 (15.3%)	90 (13.5%)	244 (15.4%)	1242 (16.0%)	23 (11.9%)	86 (18.2%)	21 (12.7%)	231 (12.6%)	.
Fair	3058 (24.1%)	154 (23.1%)	330 (20.8%)	2001 (25.7%)	41 (21.1%)	145 (30.7%)	30 (18.2%)	357 (19.5%)	.
Good	3865 (30.5%)	207 (31.0%)	524 (33.0%)	2433 (31.3%)	53 (27.3%)	101 (21.4%)	61 (37.0%)	486 (26.6%)	.
Very good	1876 (14.8%)	121 (18.1%)	287 (18.1%)	965 (12.4%)	30 (15.5%)	30 (6.4%)	7 (4.2%)	436 (23.9%)	.
Missing	791	21	54	617	8	5		86	.
Overall, how big a problem have your sexual functions been for you during the last 4 weeks?									.
No problem	6212 (48.9%)	344 (51.3%)	792 (49.7%)	3813 (49.0%)	98 (49.2%)	212 (44.9%)	128 (77.6%)	825 (45.1%)	<.0001
Very small problem	2018 (15.9%)	106 (15.8%)	248 (15.5%)	1258 (16.2%)	39 (19.6%)	89 (18.9%)	0 (0.0%)	278 (15.2%)	.
Small problem	2058 (16.2%)	105 (15.6%)	267 (16.7%)	1283 (16.5%)	26 (13.1%)	69 (14.6%)	13 (7.9%)	295 (16.1%)	.
Moderate problem	1605 (12.6%)	70 (10.4%)	190 (11.9%)	980 (12.6%)	17 (8.5%)	74 (15.7%)	18 (10.9%)	256 (14.0%)	.
Big problem	823 (6.5%)	46 (6.9%)	98 (6.1%)	450 (5.8%)	19 (9.5%)	28 (5.9%)	6 (3.6%)	176 (9.6%)	.
Missing	767	17	45	614	3	5		83	.
Hormonal									.
n	12664	675	1620	7706	201	464	165	1833	<.0001
Mean (SD)	90.2 (13.55)	92.8 (10.33)	93.2 (10.81)	89.3 (14.37)	91.2 (10.94)	93.8 (11.95)	86.9 (14.22)	89.6 (13.14)	.
Median (Q1-Q3)	95.0 (85.0 - 100.0)	95.0 (90.0 - 100.0)	100.0 (90.0 - 100.0)	95.0 (81.3 - 100.0)	95.0 (85.0 - 100.0)	100.0 (90.0 - 100.0)	90.0 (80.0 - 100.0)	95.0 (85.0 - 100.0)	.

EPIC-26 Baseline (pre-treatment) Results

Statistic Variable or Category	TNGR Global (n=13483)	Australia/New Zealand (n=688)	Canada (n=1640)	Central Europe (n=8398)	United Kingdom (n=202)	Italy (n=477)	Spain (n=165)	USA (n=1913)	P Value
Min, Max	0.0, 100.0	50.0, 100.0	10.0, 100.0	0.0, 100.0	45.0, 100.0	25.0, 100.0	30.0, 100.0	15.0, 100.0	.
How big a problem during the last 4 weeks, if any, have hot flashes been for you?									.
No problem	11460 (89.5%)	633 (93.8%)	1546 (95.4%)	6809 (86.8%)	181 (89.6%)	424 (91.2%)	159 (96.4%)	1708 (93.1%)	<.0001
Very small problem	669 (5.2%)	21 (3.1%)	46 (2.8%)	512 (6.5%)	8 (4.0%)	25 (5.4%)	0 (0.0%)	57 (3.1%)	.
Small problem	357 (2.8%)	13 (1.9%)	16 (1.0%)	274 (3.5%)	8 (4.0%)	7 (1.5%)	3 (1.8%)	36 (2.0%)	.
Moderate problem	237 (1.9%)	7 (1.0%)	11 (0.7%)	185 (2.4%)	5 (2.5%)	6 (1.3%)	2 (1.2%)	21 (1.1%)	.
Big problem	83 (0.6%)	1 (0.1%)	2 (0.1%)	63 (0.8%)	0 (0.0%)	3 (0.6%)	1 (0.6%)	13 (0.7%)	.
Missing	677	13	19	555		12		78	.
How big a problem during the last 4 weeks, if any, has breast tenderness/enlargement been for you?									.
No problem	11704 (97.6%)	662 (99.1%)	1596 (98.8%)	6857 (97.1%)	196 (98.0%)	440 (95.0%)	164 (99.4%)	1789 (98.4%)	0.0005
Very small problem	156 (1.3%)	4 (0.6%)	11 (0.7%)	111 (1.6%)	2 (1.0%)	11 (2.4%)	0 (0.0%)	17 (0.9%)	.
Small problem	72 (0.6%)	2 (0.3%)	5 (0.3%)	52 (0.7%)	1 (0.5%)	6 (1.3%)	1 (0.6%)	5 (0.3%)	.
Moderate problem	49 (0.4%)	0 (0.0%)	4 (0.2%)	30 (0.4%)	1 (0.5%)	6 (1.3%)	0 (0.0%)	8 (0.4%)	.
Big problem	10 (0.1%)	0 (0.0%)	0 (0.0%)	10 (0.1%)	0 (0.0%)	0 (0.0%)	0 (0.0%)	0 (0.0%)	.
Missing	1492	20	24	1338	2	14		94	.
How big a problem during the last 4 weeks, if any, has feeling depressed been for you?									.
No problem	8364 (65.7%)	506 (75.7%)	1204 (74.3%)	4880 (62.9%)	142 (70.6%)	370 (79.6%)	71 (43.0%)	1191 (64.6%)	<.0001
Very small problem	1929 (15.2%)	93 (13.9%)	230 (14.2%)	1208 (15.6%)	36 (17.9%)	46 (9.9%)	2 (1.2%)	314 (17.0%)	.
Small problem	1322 (10.4%)	44 (6.6%)	118 (7.3%)	919 (11.8%)	16 (8.0%)	22 (4.7%)	23 (13.9%)	180 (9.8%)	.
Moderate problem	813 (6.4%)	19 (2.8%)	58 (3.6%)	548 (7.1%)	6 (3.0%)	22 (4.7%)	41 (24.8%)	119 (6.4%)	.
Big problem	295 (2.3%)	6 (0.9%)	11 (0.7%)	203 (2.6%)	1 (0.5%)	5 (1.1%)	28 (17.0%)	41 (2.2%)	.
Missing	760	20	19	640	1	12		68	.
How big a problem during the last 4 weeks, if any, has lack of energy been for you?									.

EPIC-26 Baseline (pre-treatment) Results

Statistic Variable or Category	TNGR Global (n=13483)	Australia/New Zealand (n=688)	Canada (n=1640)	Central Europe (n=8398)	United Kingdom (n=202)	Italy (n=477)	Spain (n=165)	USA (n=1913)	P Value
No problem	7499 (58.5%)	393 (57.9%)	1024 (63.1%)	4519 (57.7%)	106 (53.0%)	359 (77.0%)	129 (78.2%)	969 (52.4%)	<.0001
Very small problem	2492 (19.4%)	155 (22.8%)	334 (20.6%)	1521 (19.4%)	49 (24.5%)	57 (12.2%)	0 (0.0%)	376 (20.3%)	.
Small problem	1609 (12.6%)	77 (11.3%)	174 (10.7%)	1034 (13.2%)	25 (12.5%)	25 (5.4%)	7 (4.2%)	267 (14.4%)	.
Moderate problem	889 (6.9%)	43 (6.3%)	69 (4.2%)	552 (7.0%)	17 (8.5%)	21 (4.5%)	14 (8.5%)	173 (9.3%)	.
Big problem	326 (2.5%)	11 (1.6%)	23 (1.4%)	204 (2.6%)	3 (1.5%)	4 (0.9%)	15 (9.1%)	66 (3.6%)	.
Missing	668	9	16	568	2	11		62	.
How big a problem during the last 4 weeks, if any, has change in body weight been for you?									.
No problem	10473 (82.0%)	576 (84.6%)	1378 (84.9%)	6328 (81.2%)	163 (83.6%)	403 (86.7%)	160 (97.0%)	1465 (79.2%)	<.0001
Very small problem	1160 (9.1%)	63 (9.3%)	145 (8.9%)	708 (9.1%)	15 (7.7%)	31 (6.7%)	0 (0.0%)	198 (10.7%)	.
Small problem	649 (5.1%)	22 (3.2%)	62 (3.8%)	422 (5.4%)	13 (6.7%)	18 (3.9%)	1 (0.6%)	111 (6.0%)	.
Moderate problem	351 (2.7%)	17 (2.5%)	25 (1.5%)	236 (3.0%)	4 (2.1%)	11 (2.4%)	4 (2.4%)	54 (2.9%)	.
Big problem	136 (1.1%)	3 (0.4%)	13 (0.8%)	97 (1.2%)	0 (0.0%)	2 (0.4%)	0 (0.0%)	21 (1.1%)	.
Missing	714	7	17	607	7	12		64	.

EPIC-26 results - Radical Prostatectomy

Statistic Variable or Category	Radical Prostatectomy (n=11094)	Australia/New Zealand (n=340)	Canada (n=794)	Central Europe (n=7754)	United Kingdom (n=72)	Italy (n=414)	Spain (n=77)	USA (n=1643)	P Value
Urinary Incontinence									.
n	10570	330	762	7318	69	414	77	1600	<.0001
Mean (SD)	93.0 (13.72)	93.0 (13.04)	93.8 (12.67)	93.3 (13.44)	97.1 (7.37)	91.4 (17.06)	94.2 (14.83)	91.4 (14.65)	.
Median (Q1-Q3)	100.0 (91.8 - 100.0)	100.0 (91.8 - 100.0)	100.0 (93.8 - 100.0)	100.0 (91.8 - 100.0)	100.0 (100.0 - 100.0)	100.0 (91.8 - 100.0)	100.0 (100.0 - 100.0)	100.0 (85.5 - 100.0)	.
Min, Max	0.0, 100.0	39.5, 100.0	6.3, 100.0	0.0, 100.0	66.8, 100.0	0.0, 100.0	33.3, 100.0	14.5, 100.0	.
Over the past 4 weeks, how often have you leaked urine?									.
More than once a day	395 (3.6%)	20 (5.9%)	29 (3.7%)	248 (3.2%)	1 (1.4%)	13 (3.1%)	1 (1.3%)	83 (5.1%)	<.0001
About once a day	376 (3.4%)	15 (4.5%)	29 (3.7%)	214 (2.8%)	2 (2.8%)	22 (5.3%)	1 (1.3%)	93 (5.7%)	.
More than once a week	317 (2.9%)	11 (3.3%)	25 (3.2%)	182 (2.4%)	0 (0.0%)	15 (3.6%)	7 (9.1%)	77 (4.7%)	.
About once a week	403 (3.7%)	16 (4.7%)	43 (5.5%)	222 (2.9%)	5 (6.9%)	21 (5.1%)	0 (0.0%)	96 (5.9%)	.
Rarely or never	9477 (86.4%)	275 (81.6%)	658 (83.9%)	6787 (88.7%)	64 (88.9%)	343 (82.9%)	68 (88.3%)	1282 (78.6%)	.
Missing	126	3	10	101				12	.
Which of the following best describes your urinary control during the last 4 weeks?									.
No urinary control whatsoever	185 (1.7%)	1 (0.3%)	10 (1.3%)	147 (1.9%)	0 (0.0%)	13 (3.1%)	0 (0.0%)	14 (0.9%)	<.0001
Frequent dribbling	274 (2.5%)	5 (1.5%)	14 (1.8%)	188 (2.5%)	0 (0.0%)	24 (5.8%)	5 (6.5%)	38 (2.3%)	.
Occasional dribbling	2421 (22.0%)	78 (23.1%)	145 (18.4%)	1678 (21.9%)	10 (13.9%)	59 (14.3%)	5 (6.5%)	446 (27.3%)	.
Total control	8111 (73.8%)	254 (75.1%)	618 (78.5%)	5655 (73.7%)	62 (86.1%)	318 (76.8%)	67 (87.0%)	1137 (69.5%)	.
Missing	103	2	7	86				8	.
How many pads or adult diapers per day did you usually use to control leakage during the last 4 weeks?									.
None	10678 (97.1%)	333 (98.8%)	762 (98.4%)	7436 (96.7%)	69 (100.0%)	389 (94.0%)	77 (100.0%)	1612 (98.6%)	<.0001
1 pad per day	237 (2.2%)	4 (1.2%)	10 (1.3%)	195 (2.5%)	0 (0.0%)	11 (2.7%)	0 (0.0%)	17 (1.0%)	.
2 pads per day	52 (0.5%)	0 (0.0%)	1 (0.1%)	39 (0.5%)	0 (0.0%)	9 (2.2%)	0 (0.0%)	3 (0.2%)	.
3 or more pads per day	30 (0.3%)	0 (0.0%)	1 (0.1%)	21 (0.3%)	0 (0.0%)	5 (1.2%)	0 (0.0%)	3 (0.2%)	.
Missing	97	3	20	63	3			8	.

EPIC-26 results - Radical Prostatectomy

Statistic Variable or Category	Radical Prostatectomy (n=11094)	Australia/New Zealand (n=340)	Canada (n=794)	Central Europe (n=7754)	United Kingdom (n=72)	Italy (n=414)	Spain (n=77)	USA (n=1643)	P Value
How big a problem, if any, has dripping or leaking urine been for you during the last 4 weeks?									.
No problem	8428 (78.5%)	256 (76.4%)	623 (79.1%)	5922 (79.5%)	63 (87.5%)	319 (77.1%)	65 (84.4%)	1180 (73.3%)	<.0001
Very small problem	1552 (14.4%)	62 (18.5%)	126 (16.0%)	995 (13.4%)	7 (9.7%)	59 (14.3%)	0 (0.0%)	303 (18.8%)	.
Small problem	483 (4.5%)	12 (3.6%)	28 (3.6%)	322 (4.3%)	1 (1.4%)	25 (6.0%)	6 (7.8%)	89 (5.5%)	.
Moderate problem	216 (2.0%)	5 (1.5%)	9 (1.1%)	156 (2.1%)	1 (1.4%)	8 (1.9%)	5 (6.5%)	32 (2.0%)	.
Big problem	62 (0.6%)	0 (0.0%)	2 (0.3%)	51 (0.7%)	0 (0.0%)	3 (0.7%)	1 (1.3%)	5 (0.3%)	.
Missing	353	5	6	308				34	.
Urinary Irritative/Obst.									.
n	10502	319	787	7254	69	414	77	1582	0.0011
Mean (SD)	85.8 (15.62)	85.3 (15.90)	86.5 (15.07)	86.0 (15.55)	85.8 (12.12)	84.0 (17.63)	89.0 (15.70)	84.8 (15.70)	.
Median (Q1-Q3)	87.5 (75.0 - 100.0)	87.5 (81.3 - 100.0)	93.8 (75.0 - 100.0)	87.5 (75.0 - 100.0)	87.5 (75.0 - 93.8)	87.5 (75.0 - 100.0)	100.0 (87.5 - 100.0)	87.5 (75.0 - 100.0)	.
Min, Max	0.0, 100.0	12.5, 100.0	12.5, 100.0	0.0, 100.0	56.3, 100.0	18.8, 100.0	25.0, 100.0	6.3, 100.0	.
How big a problem, if any, has pain or burning on urination been for you during the last 4 weeks?									.
No problem	9300 (86.9%)	290 (86.6%)	688 (87.0%)	6490 (87.5%)	61 (85.9%)	310 (74.9%)	68 (88.3%)	1393 (86.8%)	<.0001
Very small problem	829 (7.7%)	32 (9.6%)	71 (9.0%)	537 (7.2%)	6 (8.5%)	69 (16.7%)	0 (0.0%)	114 (7.1%)	.
Small problem	317 (3.0%)	5 (1.5%)	17 (2.1%)	200 (2.7%)	3 (4.2%)	21 (5.1%)	7 (9.1%)	64 (4.0%)	.
Moderate problem	186 (1.7%)	7 (2.1%)	10 (1.3%)	128 (1.7%)	0 (0.0%)	12 (2.9%)	1 (1.3%)	28 (1.7%)	.
Big problem	75 (0.7%)	1 (0.3%)	5 (0.6%)	60 (0.8%)	1 (1.4%)	2 (0.5%)	1 (1.3%)	5 (0.3%)	.
Missing	387	5	3	339	1			39	.
How big a problem, if any, has bleeding with urination been for you during the last 4 weeks?									.
No problem	10059 (94.5%)	302 (89.6%)	732 (92.4%)	7023 (95.4%)	64 (91.4%)	382 (92.3%)	77 (100.0%)	1479 (92.4%)	<.0001
Very small problem	352 (3.3%)	24 (7.1%)	45 (5.7%)	184 (2.5%)	5 (7.1%)	14 (3.4%)	0 (0.0%)	80 (5.0%)	.
Small problem	144 (1.4%)	8 (2.4%)	8 (1.0%)	89 (1.2%)	1 (1.4%)	11 (2.7%)	0 (0.0%)	27 (1.7%)	.
Moderate problem	70 (0.7%)	3 (0.9%)	7 (0.9%)	45 (0.6%)	0 (0.0%)	4 (1.0%)	0 (0.0%)	11 (0.7%)	.
Big problem	25 (0.2%)	0 (0.0%)	0 (0.0%)	19 (0.3%)	0 (0.0%)	3 (0.7%)	0 (0.0%)	3 (0.2%)	.

EPIC-26 results - Radical Prostatectomy

Statistic Variable or Category	Radical Prostatectomy (n=11094)	Australia/New Zealand (n=340)	Canada (n=794)	Central Europe (n=7754)	United Kingdom (n=72)	Italy (n=414)	Spain (n=77)	USA (n=1643)	P Value
Missing	444	3	2	394	2			43	.
How big a problem, if any, has weak urine stream been for you during the last 4 weeks?									.
No problem	5172 (47.9%)	153 (45.8%)	397 (50.4%)	3567 (47.5%)	32 (45.7%)	211 (51.0%)	47 (61.0%)	765 (47.5%)	0.0023
Very small problem	2643 (24.5%)	95 (28.4%)	189 (24.0%)	1871 (24.9%)	18 (25.7%)	89 (21.5%)	1 (1.3%)	380 (23.6%)	.
Small problem	1604 (14.9%)	43 (12.9%)	128 (16.2%)	1106 (14.7%)	12 (17.1%)	58 (14.0%)	18 (23.4%)	239 (14.8%)	.
Moderate problem	1037 (9.6%)	33 (9.9%)	56 (7.1%)	718 (9.6%)	8 (11.4%)	47 (11.4%)	9 (11.7%)	166 (10.3%)	.
Big problem	342 (3.2%)	10 (3.0%)	18 (2.3%)	243 (3.2%)	0 (0.0%)	9 (2.2%)	2 (2.6%)	60 (3.7%)	.
Missing	296	6	6	249	2			33	.
How big a problem, if any, has need to urinate frequently during the day been for you during the last 4 weeks?									.
No problem	4930 (45.4%)	149 (45.6%)	373 (47.2%)	3495 (46.3%)	28 (38.9%)	185 (44.7%)	59 (76.6%)	641 (39.6%)	<.0001
Very small problem	2439 (22.5%)	76 (23.2%)	188 (23.8%)	1659 (22.0%)	20 (27.8%)	88 (21.3%)	1 (1.3%)	407 (25.1%)	.
Small problem	1736 (16.0%)	50 (15.3%)	114 (14.4%)	1201 (15.9%)	15 (20.8%)	64 (15.5%)	10 (13.0%)	282 (17.4%)	.
Moderate problem	1334 (12.3%)	43 (13.1%)	95 (12.0%)	895 (11.9%)	9 (12.5%)	62 (15.0%)	6 (7.8%)	224 (13.8%)	.
Big problem	411 (3.8%)	9 (2.8%)	21 (2.7%)	300 (4.0%)	0 (0.0%)	15 (3.6%)	1 (1.3%)	65 (4.0%)	.
Missing	244	13	3	204				24	.
Overall how big a problem has your urinary function been for you during the last 4 weeks?									.
No problem	6351 (58.3%)	178 (52.5%)	460 (58.3%)	4578 (60.5%)	37 (51.4%)	210 (50.7%)	47 (61.0%)	841 (51.4%)	<.0001
Very small problem	2266 (20.8%)	98 (28.9%)	175 (22.2%)	1465 (19.4%)	18 (25.0%)	94 (22.7%)	1 (1.3%)	415 (25.4%)	.
Small problem	1177 (10.8%)	33 (9.7%)	86 (10.9%)	764 (10.1%)	11 (15.3%)	55 (13.3%)	17 (22.1%)	211 (12.9%)	.
Moderate problem	855 (7.9%)	27 (8.0%)	55 (7.0%)	579 (7.7%)	6 (8.3%)	48 (11.6%)	10 (13.0%)	130 (8.0%)	.
Big problem	242 (2.2%)	3 (0.9%)	13 (1.6%)	179 (2.4%)	0 (0.0%)	7 (1.7%)	2 (2.6%)	38 (2.3%)	.
Missing	203	1	5	189				8	.
Bowel									.
n	10590	336	791	7288	69	414	77	1615	<.0001
Mean (SD)	95.5 (9.84)	93.6 (11.31)	94.9 (10.69)	96.0 (9.29)	94.8 (11.15)	93.4 (11.50)	98.4 (5.70)	94.4 (10.84)	.

EPIC-26 results - Radical Prostatectomy

Statistic Variable or Category	Radical Prostatectomy (n=11094)	Australia/New Zealand (n=340)	Canada (n=794)	Central Europe (n=7754)	United Kingdom (n=72)	Italy (n=414)	Spain (n=77)	USA (n=1643)	P Value
Median (Q1-Q3)	100.0 (95.8 - 100.0)	100.0 (91.7 - 100.0)	100.0 (95.8 - 100.0)	100.0 (95.8 - 100.0)	100.0 (95.8 - 100.0)	100.0 (91.7 - 100.0)	100.0 (100.0 - 100.0)	100.0 (91.7 - 100.0)	.
Min, Max	8.3, 100.0	33.3, 100.0	12.5, 100.0	8.3, 100.0	45.8, 100.0	41.7, 100.0	66.7, 100.0	12.5, 100.0	.
How big a problem, if any, has urgency to have a bowel movement been for you?									.
No problem	9231 (84.2%)	238 (70.6%)	638 (80.6%)	6625 (86.7%)	63 (87.5%)	322 (77.8%)	77 (100.0%)	1268 (77.9%)	<.0001
Very small problem	960 (8.8%)	65 (19.3%)	80 (10.1%)	534 (7.0%)	4 (5.6%)	53 (12.8%)	0 (0.0%)	224 (13.8%)	.
Small problem	445 (4.1%)	21 (6.2%)	35 (4.4%)	288 (3.8%)	2 (2.8%)	21 (5.1%)	0 (0.0%)	78 (4.8%)	.
Moderate problem	258 (2.4%)	10 (3.0%)	33 (4.2%)	150 (2.0%)	2 (2.8%)	13 (3.1%)	0 (0.0%)	50 (3.1%)	.
Big problem	65 (0.6%)	3 (0.9%)	6 (0.8%)	43 (0.6%)	1 (1.4%)	5 (1.2%)	0 (0.0%)	7 (0.4%)	.
Missing	135	3	2	114				16	.
How big a problem, if any, has increased frequency of bowel movements been for you?									.
No problem	9227 (86.8%)	261 (77.9%)	679 (85.8%)	6457 (88.1%)	58 (84.1%)	346 (83.6%)	77 (100.0%)	1349 (83.5%)	<.0001
Very small problem	823 (7.7%)	41 (12.2%)	69 (8.7%)	510 (7.0%)	8 (11.6%)	38 (9.2%)	0 (0.0%)	157 (9.7%)	.
Small problem	358 (3.4%)	23 (6.9%)	21 (2.7%)	226 (3.1%)	1 (1.4%)	20 (4.8%)	0 (0.0%)	67 (4.1%)	.
Moderate problem	178 (1.7%)	7 (2.1%)	20 (2.5%)	102 (1.4%)	2 (2.9%)	10 (2.4%)	0 (0.0%)	37 (2.3%)	.
Big problem	43 (0.4%)	3 (0.9%)	2 (0.3%)	33 (0.5%)	0 (0.0%)	0 (0.0%)	0 (0.0%)	5 (0.3%)	.
Missing	465	5	3	426	3			28	.
How big a problem, if any, has losing control of your stools been for you?									.
No problem	10194 (96.2%)	311 (92.8%)	761 (96.5%)	7070 (97.0%)	68 (94.4%)	391 (94.4%)	77 (100.0%)	1516 (93.9%)	<.0001
Very small problem	264 (2.5%)	19 (5.7%)	17 (2.2%)	150 (2.1%)	3 (4.2%)	13 (3.1%)	0 (0.0%)	62 (3.8%)	.
Small problem	87 (0.8%)	3 (0.9%)	7 (0.9%)	40 (0.5%)	0 (0.0%)	9 (2.2%)	0 (0.0%)	28 (1.7%)	.
Moderate problem	32 (0.3%)	1 (0.3%)	2 (0.3%)	20 (0.3%)	1 (1.4%)	1 (0.2%)	0 (0.0%)	7 (0.4%)	.
Big problem	17 (0.2%)	1 (0.3%)	2 (0.3%)	12 (0.2%)	0 (0.0%)	0 (0.0%)	0 (0.0%)	2 (0.1%)	.
Missing	500	5	5	462				28	.
How big a problem, if any, has bloody stools been for you?									.

EPIC-26 results - Radical Prostatectomy

Statistic Variable or Category	Radical Prostatectomy (n=11094)	Australia/New Zealand (n=340)	Canada (n=794)	Central Europe (n=7754)	United Kingdom (n=72)	Italy (n=414)	Spain (n=77)	USA (n=1643)	P Value
No problem	10261 (96.8%)	323 (95.8%)	754 (95.2%)	7104 (97.4%)	68 (98.6%)	395 (95.4%)	74 (96.1%)	1543 (95.5%)	0.0056
Very small problem	234 (2.2%)	13 (3.9%)	28 (3.5%)	128 (1.8%)	1 (1.4%)	11 (2.7%)	1 (1.3%)	52 (3.2%)	.
Small problem	76 (0.7%)	1 (0.3%)	6 (0.8%)	47 (0.6%)	0 (0.0%)	7 (1.7%)	2 (2.6%)	13 (0.8%)	.
Moderate problem	24 (0.2%)	0 (0.0%)	3 (0.4%)	13 (0.2%)	0 (0.0%)	1 (0.2%)	0 (0.0%)	7 (0.4%)	.
Big problem	5 (0.0%)	0 (0.0%)	1 (0.1%)	3 (0.0%)	0 (0.0%)	0 (0.0%)	0 (0.0%)	1 (0.1%)	.
Missing	494	3	2	459	3			27	.
How big a problem, if any, has abdominal/pelvic/rectal pain been for you?									.
No problem	8998 (84.7%)	273 (81.5%)	654 (82.6%)	6274 (85.6%)	54 (77.1%)	329 (79.5%)	73 (94.8%)	1341 (83.2%)	0.0040
Very small problem	943 (8.9%)	41 (12.2%)	87 (11.0%)	605 (8.3%)	6 (8.6%)	47 (11.4%)	0 (0.0%)	157 (9.7%)	.
Small problem	391 (3.7%)	13 (3.9%)	27 (3.4%)	262 (3.6%)	6 (8.6%)	22 (5.3%)	1 (1.3%)	60 (3.7%)	.
Moderate problem	229 (2.2%)	6 (1.8%)	18 (2.3%)	145 (2.0%)	3 (4.3%)	13 (3.1%)	3 (3.9%)	41 (2.5%)	.
Big problem	65 (0.6%)	2 (0.6%)	6 (0.8%)	40 (0.5%)	1 (1.4%)	3 (0.7%)	0 (0.0%)	13 (0.8%)	.
Missing	468	5	2	428	2			31	.
Overall, how big a problem have your bowel habits been for you during the last 4 weeks?									.
No problem	9230 (84.0%)	260 (77.6%)	652 (82.5%)	6586 (85.9%)	61 (84.7%)	309 (74.6%)	72 (93.5%)	1290 (78.9%)	<.0001
Very small problem	1069 (9.7%)	51 (15.2%)	94 (11.9%)	634 (8.3%)	8 (11.1%)	49 (11.8%)	0 (0.0%)	233 (14.3%)	.
Small problem	397 (3.6%)	10 (3.0%)	27 (3.4%)	249 (3.2%)	1 (1.4%)	35 (8.5%)	2 (2.6%)	73 (4.5%)	.
Moderate problem	220 (2.0%)	12 (3.6%)	14 (1.8%)	140 (1.8%)	2 (2.8%)	17 (4.1%)	3 (3.9%)	32 (2.0%)	.
Big problem	70 (0.6%)	2 (0.6%)	3 (0.4%)	55 (0.7%)	0 (0.0%)	4 (1.0%)	0 (0.0%)	6 (0.4%)	.
Missing	108	5	4	90				9	.
Sexual									.
n	10341	327	779	7106	68	414	77	1570	<.0001
Mean (SD)	62.8 (29.04)	67.2 (27.68)	68.0 (26.58)	62.1 (28.95)	63.3 (32.29)	54.4 (26.78)	74.4 (21.56)	64.2 (30.82)	.
Median (Q1-Q3)	66.7 (40.3 - 87.5)	75.0 (44.5 - 87.5)	75.0 (48.7 - 87.5)	66.7 (38.8 - 87.5)	70.8 (30.5 - 89.6)	58.3 (34.7 - 75.0)	83.3 (66.7 - 87.5)	70.8 (40.3 - 91.7)	.
Min, Max	0.0, 100.0	0.0, 100.0	0.0, 100.0	0.0, 100.0	0.0, 100.0	0.0, 100.0	9.7, 100.0	0.0, 100.0	.

EPIC-26 results - Radical Prostatectomy

Statistic Variable or Category	Radical Prostatectomy (n=11094)	Australia/New Zealand (n=340)	Canada (n=794)	Central Europe (n=7754)	United Kingdom (n=72)	Italy (n=414)	Spain (n=77)	USA (n=1643)	P Value
How would you rate your ability to have an erection during the last 4 weeks?									.
Very poor to none	1390 (13.2%)	33 (9.9%)	65 (8.3%)	957 (13.1%)	16 (22.5%)	74 (17.9%)	5 (6.5%)	240 (15.0%)	<.0001
Poor	1528 (14.5%)	48 (14.4%)	109 (13.9%)	1053 (14.4%)	8 (11.3%)	89 (21.5%)	4 (5.2%)	217 (13.6%)	.
Fair	2665 (25.2%)	69 (20.7%)	182 (23.3%)	1914 (26.2%)	12 (16.9%)	126 (30.4%)	10 (13.0%)	352 (22.1%)	.
Good	3197 (30.3%)	108 (32.3%)	282 (36.1%)	2227 (30.5%)	20 (28.2%)	94 (22.7%)	55 (71.4%)	411 (25.8%)	.
Very good	1788 (16.9%)	76 (22.8%)	144 (18.4%)	1143 (15.7%)	15 (21.1%)	31 (7.5%)	3 (3.9%)	376 (23.6%)	.
Missing	526	6	12	460	1			47	.
How would you rate your ability to reach orgasm (climax) during the last 4 weeks?									.
Very poor to none	1294 (12.5%)	37 (11.3%)	71 (9.1%)	881 (12.3%)	13 (18.6%)	71 (17.1%)	7 (9.1%)	214 (13.6%)	<.0001
Poor	1261 (12.1%)	42 (12.8%)	74 (9.5%)	899 (12.6%)	7 (10.0%)	68 (16.4%)	2 (2.6%)	169 (10.8%)	.
Fair	2291 (22.1%)	62 (18.9%)	161 (20.6%)	1646 (23.0%)	16 (22.9%)	131 (31.6%)	4 (5.2%)	271 (17.3%)	.
Good	3512 (33.8%)	111 (33.8%)	292 (37.4%)	2475 (34.6%)	19 (27.1%)	107 (25.8%)	62 (80.5%)	446 (28.4%)	.
Very good	2031 (19.5%)	76 (23.2%)	182 (23.3%)	1251 (17.5%)	15 (21.4%)	37 (8.9%)	2 (2.6%)	468 (29.8%)	.
Missing	705	12	14	602	2			75	.
How would you describe the usual quality of your erections during the last 4 weeks?									.
None at all	1440 (13.7%)	29 (8.7%)	53 (6.8%)	1086 (15.0%)	11 (15.5%)	62 (15.0%)	4 (5.2%)	195 (12.3%)	<.0001
Not firm enough for any sexual activity	1332 (12.7%)	35 (10.5%)	101 (12.9%)	929 (12.9%)	9 (12.7%)	67 (16.2%)	2 (2.6%)	189 (11.9%)	.
Firm enough for masturbation and foreplay	1966 (18.8%)	66 (19.9%)	150 (19.2%)	1388 (19.2%)	13 (18.3%)	66 (15.9%)	6 (7.8%)	277 (17.5%)	.
Firm enough for intercourse	5737 (54.8%)	202 (60.8%)	477 (61.1%)	3813 (52.8%)	38 (53.5%)	219 (52.9%)	65 (84.4%)	923 (58.3%)	.
Missing	619	8	13	538	1			59	.
How would you describe the frequency of your erections during the last 4 weeks?									.
I never had an erection when I wanted one	1484 (14.5%)	27 (8.3%)	64 (8.3%)	1073 (15.3%)	12 (17.1%)	67 (16.2%)	9 (11.7%)	232 (14.8%)	<.0001

EPIC-26 results - Radical Prostatectomy

Statistic Variable or Category	Radical Prostatectomy (n=11094)	Australia/New Zealand (n=340)	Canada (n=794)	Central Europe (n=7754)	United Kingdom (n=72)	Italy (n=414)	Spain (n=77)	USA (n=1643)	P Value
I had an erection less than half the time I wanted one	1452 (14.2%)	42 (12.8%)	101 (13.0%)	1049 (15.0%)	9 (12.9%)	66 (15.9%)	1 (1.3%)	184 (11.7%)	.
I had an erection about half the time I wanted one	1502 (14.7%)	41 (12.5%)	113 (14.6%)	1036 (14.8%)	6 (8.6%)	123 (29.7%)	5 (6.5%)	178 (11.3%)	.
I had an erection more than half the time I wanted one	1693 (16.5%)	65 (19.9%)	124 (16.0%)	1116 (15.9%)	12 (17.1%)	83 (20.0%)	4 (5.2%)	289 (18.4%)	.
I had an erection whenever I wanted one	4113 (40.2%)	152 (46.5%)	372 (48.1%)	2736 (39.0%)	31 (44.3%)	75 (18.1%)	58 (75.3%)	689 (43.8%)	.
Missing	850	13	20	744	2			71	.
Overall, how would you rate your ability to function sexually during the last 4 weeks?									.
Very poor	1504 (14.4%)	45 (13.7%)	75 (9.6%)	998 (13.9%)	13 (19.1%)	93 (22.5%)	10 (13.0%)	270 (17.1%)	<.0001
Poor	1596 (15.3%)	42 (12.8%)	119 (15.3%)	1131 (15.7%)	8 (11.8%)	80 (19.3%)	11 (14.3%)	205 (13.0%)	.
Fair	2541 (24.3%)	79 (24.0%)	160 (20.5%)	1842 (25.6%)	13 (19.1%)	128 (30.9%)	13 (16.9%)	306 (19.4%)	.
Good	3245 (31.1%)	104 (31.6%)	272 (34.9%)	2296 (31.9%)	20 (29.4%)	88 (21.3%)	37 (48.1%)	428 (27.1%)	.
Very good	1558 (14.9%)	59 (17.9%)	154 (19.7%)	928 (12.9%)	14 (20.6%)	25 (6.0%)	6 (7.8%)	372 (23.5%)	.
Missing	650	11	14	559	4			62	.
Overall, how big a problem have your sexual functions been for you during the last 4 weeks?									.
No problem	5090 (48.7%)	170 (51.1%)	393 (50.3%)	3539 (49.2%)	38 (54.3%)	180 (43.5%)	62 (80.5%)	708 (44.7%)	<.0001
Very small problem	1693 (16.2%)	59 (17.7%)	135 (17.3%)	1159 (16.1%)	16 (22.9%)	79 (19.1%)	0 (0.0%)	245 (15.5%)	.
Small problem	1682 (16.1%)	50 (15.0%)	120 (15.3%)	1187 (16.5%)	5 (7.1%)	65 (15.7%)	5 (6.5%)	250 (15.8%)	.
Moderate problem	1343 (12.8%)	29 (8.7%)	97 (12.4%)	903 (12.6%)	5 (7.1%)	70 (16.9%)	7 (9.1%)	232 (14.6%)	.
Big problem	645 (6.2%)	25 (7.5%)	37 (4.7%)	405 (5.6%)	6 (8.6%)	20 (4.8%)	3 (3.9%)	149 (9.4%)	.
Missing	641	7	12	561	2			59	.
Hormonal									.
n	10399	340	782	7131	72	414	77	1583	<.0001
Mean (SD)	89.9 (13.81)	93.2 (10.03)	92.3 (11.59)	89.3 (14.35)	91.9 (10.48)	94.0 (11.80)	86.2 (13.77)	89.4 (13.22)	.
Median (Q1-Q3)	95.0 (85.0 - 100.0)	100.0 (90.0 - 100.0)	100.0 (90.0 - 100.0)	95.0 (81.3 - 100.0)	95.0 (90.0 - 100.0)	100.0 (90.0 - 100.0)	85.0 (80.0 - 100.0)	95.0 (85.0 - 100.0)	.

EPIC-26 results - Radical Prostatectomy

Statistic Variable or Category	Radical Prostatectomy (n=11094)	Australia/New Zealand (n=340)	Canada (n=794)	Central Europe (n=7754)	United Kingdom (n=72)	Italy (n=414)	Spain (n=77)	USA (n=1643)	P Value
Min, Max	5.0, 100.0	50.0, 100.0	10.0, 100.0	5.0, 100.0	60.0, 100.0	25.0, 100.0	45.0, 100.0	15.0, 100.0	.
How big a problem during the last 4 weeks, if any, have hot flashes been for you?									.
No problem	9335 (88.7%)	324 (95.3%)	742 (94.8%)	6288 (86.7%)	65 (90.3%)	374 (90.3%)	77 (100.0%)	1465 (92.5%)	<.0001
Very small problem	598 (5.7%)	8 (2.4%)	27 (3.4%)	481 (6.6%)	3 (4.2%)	25 (6.0%)	0 (0.0%)	54 (3.4%)	.
Small problem	301 (2.9%)	5 (1.5%)	6 (0.8%)	249 (3.4%)	3 (4.2%)	6 (1.4%)	0 (0.0%)	32 (2.0%)	.
Moderate problem	213 (2.0%)	3 (0.9%)	7 (0.9%)	176 (2.4%)	1 (1.4%)	6 (1.4%)	0 (0.0%)	20 (1.3%)	.
Big problem	73 (0.7%)	0 (0.0%)	1 (0.1%)	57 (0.8%)	0 (0.0%)	3 (0.7%)	0 (0.0%)	12 (0.8%)	.
Missing	574		11	503				60	.
How big a problem during the last 4 weeks, if any, has breast tenderness/enlargement been for you?									.
No problem	9559 (97.6%)	337 (99.4%)	768 (98.6%)	6367 (97.3%)	68 (94.4%)	394 (95.2%)	77 (100.0%)	1548 (98.5%)	0.0238
Very small problem	133 (1.4%)	2 (0.6%)	6 (0.8%)	96 (1.5%)	2 (2.8%)	11 (2.7%)	0 (0.0%)	16 (1.0%)	.
Small problem	60 (0.6%)	0 (0.0%)	3 (0.4%)	49 (0.7%)	1 (1.4%)	4 (1.0%)	0 (0.0%)	3 (0.2%)	.
Moderate problem	39 (0.4%)	0 (0.0%)	2 (0.3%)	26 (0.4%)	1 (1.4%)	5 (1.2%)	0 (0.0%)	5 (0.3%)	.
Big problem	8 (0.1%)	0 (0.0%)	0 (0.0%)	8 (0.1%)	0 (0.0%)	0 (0.0%)	0 (0.0%)	0 (0.0%)	.
Missing	1295	1	15	1208				71	.
How big a problem during the last 4 weeks, if any, has feeling depressed been for you?									.
No problem	6710 (64.2%)	253 (75.5%)	540 (68.9%)	4492 (62.6%)	52 (72.2%)	335 (80.9%)	27 (35.1%)	1011 (63.5%)	<.0001
Very small problem	1627 (15.6%)	49 (14.6%)	131 (16.7%)	1117 (15.6%)	10 (13.9%)	38 (9.2%)	2 (2.6%)	280 (17.6%)	.
Small problem	1136 (10.9%)	21 (6.3%)	69 (8.8%)	853 (11.9%)	9 (12.5%)	18 (4.3%)	8 (10.4%)	158 (9.9%)	.
Moderate problem	714 (6.8%)	10 (3.0%)	37 (4.7%)	521 (7.3%)	0 (0.0%)	19 (4.6%)	23 (29.9%)	104 (6.5%)	.
Big problem	260 (2.5%)	2 (0.6%)	7 (0.9%)	191 (2.7%)	1 (1.4%)	4 (1.0%)	17 (22.1%)	38 (2.4%)	.
Missing	647	5	10	580				52	.
How big a problem during the last 4 weeks, if any, has lack of energy been for you?									.

EPIC-26 results - Radical Prostatectomy

Statistic Variable or Category	Radical Prostatectomy (n=11094)	Australia/New Zealand (n=340)	Canada (n=794)	Central Europe (n=7754)	United Kingdom (n=72)	Italy (n=414)	Spain (n=77)	USA (n=1643)	P Value
No problem	6119 (58.2%)	200 (59.0%)	475 (60.5%)	4187 (57.9%)	42 (58.3%)	323 (78.0%)	62 (80.5%)	830 (52.0%)	<.0001
Very small problem	2047 (19.5%)	79 (23.3%)	172 (21.9%)	1403 (19.4%)	15 (20.8%)	51 (12.3%)	0 (0.0%)	327 (20.5%)	.
Small problem	1325 (12.6%)	32 (9.4%)	87 (11.1%)	944 (13.0%)	11 (15.3%)	21 (5.1%)	2 (2.6%)	228 (14.3%)	.
Moderate problem	751 (7.1%)	21 (6.2%)	38 (4.8%)	515 (7.1%)	3 (4.2%)	15 (3.6%)	4 (5.2%)	155 (9.7%)	.
Big problem	279 (2.7%)	7 (2.1%)	13 (1.7%)	188 (2.6%)	1 (1.4%)	4 (1.0%)	9 (11.7%)	57 (3.6%)	.
Missing	573	1	9	517				46	.
How big a problem during the last 4 weeks, if any, has change in body weight been for you?									.
No problem	8558 (81.6%)	299 (87.9%)	662 (84.3%)	5848 (81.2%)	61 (87.1%)	359 (86.7%)	75 (97.4%)	1254 (78.7%)	<.0001
Very small problem	959 (9.1%)	23 (6.8%)	72 (9.2%)	652 (9.1%)	5 (7.1%)	29 (7.0%)	0 (0.0%)	178 (11.2%)	.
Small problem	547 (5.2%)	8 (2.4%)	31 (3.9%)	397 (5.5%)	2 (2.9%)	14 (3.4%)	1 (1.3%)	94 (5.9%)	.
Moderate problem	295 (2.8%)	9 (2.6%)	10 (1.3%)	214 (3.0%)	2 (2.9%)	11 (2.7%)	1 (1.3%)	48 (3.0%)	.
Big problem	123 (1.2%)	1 (0.3%)	10 (1.3%)	91 (1.3%)	0 (0.0%)	1 (0.2%)	0 (0.0%)	20 (1.3%)	.
Missing	612		9	552	2			49	.

EPIC-26 results - EBRT

Statistic Variable or Category	EBRT (n=1037)	Australia/New Zealand (n=104)	Canada (n=354)	Central Europe (n=439)	United Kingdom (n=53)	Italy (n=24)	Spain (n=47)	USA (n=16)	P Value
Urinary Incontinence									.
n	983	102	344	401	52	21	47	16	0.0448
Mean (SD)	91.5 (15.85)	94.8 (11.43)	91.9 (14.02)	91.0 (16.96)	91.6 (15.39)	94.5 (10.58)	90.4 (20.73)	76.0 (28.04)	.
Median (Q1-Q3)	100.0 (87.5 - 100.0)	100.0 (100.0 - 100.0)	100.0 (85.5 - 100.0)	100.0 (91.8 - 100.0)	100.0 (85.5 - 100.0)	100.0 (91.8 - 100.0)	100.0 (100.0 - 100.0)	92.8 (50.0 - 100.0)	.
Min, Max	6.3, 100.0	52.0, 100.0	25.0, 100.0	6.3, 100.0	14.5, 100.0	60.5, 100.0	33.3, 100.0	25.0, 100.0	.
Over the past 4 weeks, how often have you leaked urine?									.
More than once a day	63 (6.1%)	4 (3.9%)	23 (6.6%)	26 (5.9%)	2 (3.8%)	0 (0.0%)	2 (4.3%)	6 (37.5%)	0.0010
About once a day	33 (3.2%)	4 (3.9%)	8 (2.3%)	12 (2.7%)	3 (5.7%)	1 (4.3%)	4 (8.5%)	1 (6.3%)	.
More than once a week	26 (2.5%)	3 (2.9%)	10 (2.8%)	9 (2.1%)	2 (3.8%)	0 (0.0%)	2 (4.3%)	0 (0.0%)	.
About once a week	58 (5.6%)	3 (2.9%)	30 (8.5%)	21 (4.8%)	3 (5.7%)	0 (0.0%)	1 (2.1%)	0 (0.0%)	.
Rarely or never	850 (82.5%)	89 (86.4%)	280 (79.8%)	369 (84.4%)	43 (81.1%)	22 (95.7%)	38 (80.9%)	9 (56.3%)	.
Missing	7	1	3	2		1			.
Which of the following best describes your urinary control during the last 4 weeks?									.
No urinary control whatsoever	24 (2.3%)	0 (0.0%)	5 (1.4%)	16 (3.7%)	0 (0.0%)	1 (4.3%)	0 (0.0%)	2 (12.5%)	0.0002
Frequent dribbling	43 (4.2%)	1 (1.0%)	13 (3.7%)	17 (3.9%)	3 (5.8%)	0 (0.0%)	6 (12.8%)	3 (18.8%)	.
Occasional dribbling	220 (21.4%)	18 (17.5%)	80 (22.9%)	98 (22.5%)	13 (25.0%)	5 (21.7%)	3 (6.4%)	3 (18.8%)	.
Total control	739 (72.0%)	84 (81.6%)	252 (72.0%)	304 (69.9%)	36 (69.2%)	17 (73.9%)	38 (80.9%)	8 (50.0%)	.
Missing	11	1	4	4	1	1			.
How many pads or adult diapers per day did you usually use to control leakage during the last 4 weeks?									.
None	1001 (97.4%)	103 (100.0%)	345 (99.1%)	416 (95.0%)	52 (98.1%)	23 (100.0%)	47 (100.0%)	15 (93.8%)	0.1171
1 pad per day	19 (1.8%)	0 (0.0%)	2 (0.6%)	16 (3.7%)	0 (0.0%)	0 (0.0%)	0 (0.0%)	1 (6.3%)	.
2 pads per day	4 (0.4%)	0 (0.0%)	1 (0.3%)	3 (0.7%)	0 (0.0%)	0 (0.0%)	0 (0.0%)	0 (0.0%)	.
3 or more pads per day	4 (0.4%)	0 (0.0%)	0 (0.0%)	3 (0.7%)	1 (1.9%)	0 (0.0%)	0 (0.0%)	0 (0.0%)	.
Missing	9	1	6	1		1			.

EPIC-26 results - EBRT

Statistic Variable or Category	EBRT (n=1037)	Australia/New Zealand (n=104)	Canada (n=354)	Central Europe (n=439)	United Kingdom (n=53)	Italy (n=24)	Spain (n=47)	USA (n=16)	P Value
How big a problem, if any, has dripping or leaking urine been for you during the last 4 weeks?									.
No problem	750 (75.3%)	85 (83.3%)	258 (73.3%)	304 (75.1%)	37 (69.8%)	17 (81.0%)	38 (80.9%)	11 (68.8%)	<.0001
Very small problem	163 (16.4%)	12 (11.8%)	69 (19.6%)	63 (15.6%)	15 (28.3%)	3 (14.3%)	0 (0.0%)	1 (6.3%)	.
Small problem	46 (4.6%)	5 (4.9%)	15 (4.3%)	21 (5.2%)	0 (0.0%)	1 (4.8%)	3 (6.4%)	1 (6.3%)	.
Moderate problem	29 (2.9%)	0 (0.0%)	9 (2.6%)	14 (3.5%)	1 (1.9%)	0 (0.0%)	3 (6.4%)	2 (12.5%)	.
Big problem	8 (0.8%)	0 (0.0%)	1 (0.3%)	3 (0.7%)	0 (0.0%)	0 (0.0%)	3 (6.4%)	1 (6.3%)	.
Missing	41	2	2	34		3			.
Urinary Irritative/Obst.									.
n	975	101	351	390	52	20	47	14	0.1532
Mean (SD)	86.6 (15.14)	86.6 (13.75)	86.8 (14.67)	86.5 (16.10)	83.2 (15.03)	92.8 (9.13)	88.3 (13.27)	81.3 (19.76)	.
Median (Q1-Q3)	87.5 (81.3 - 100.0)	93.8 (81.3 - 100.0)	93.8 (81.3 - 100.0)	93.8 (81.3 - 100.0)	87.5 (75.0 - 93.8)	93.8 (87.5 - 100.0)	87.5 (81.3 - 100.0)	90.6 (62.5 - 100.0)	.
Min, Max	18.8, 100.0	43.8, 100.0	25.0, 100.0	18.8, 100.0	31.3, 100.0	68.8, 100.0	50.0, 100.0	37.5, 100.0	.
How big a problem, if any, has pain or burning on urination been for you during the last 4 weeks?									.
No problem	862 (86.6%)	89 (87.3%)	313 (88.9%)	344 (84.5%)	47 (88.7%)	19 (95.0%)	38 (80.9%)	12 (85.7%)	0.0013
Very small problem	77 (7.7%)	11 (10.8%)	30 (8.5%)	30 (7.4%)	4 (7.5%)	1 (5.0%)	1 (2.1%)	0 (0.0%)	.
Small problem	30 (3.0%)	1 (1.0%)	3 (0.9%)	18 (4.4%)	0 (0.0%)	0 (0.0%)	7 (14.9%)	1 (7.1%)	.
Moderate problem	17 (1.7%)	1 (1.0%)	3 (0.9%)	9 (2.2%)	2 (3.8%)	0 (0.0%)	1 (2.1%)	1 (7.1%)	.
Big problem	9 (0.9%)	0 (0.0%)	3 (0.9%)	6 (1.5%)	0 (0.0%)	0 (0.0%)	0 (0.0%)	0 (0.0%)	.
Missing	42	2	2	32		4		2	.
How big a problem, if any, has bleeding with urination been for you during the last 4 weeks?									.
No problem	916 (93.3%)	93 (92.1%)	326 (92.9%)	366 (92.2%)	51 (98.1%)	20 (100.0%)	47 (100.0%)	13 (92.9%)	0.6380
Very small problem	39 (4.0%)	6 (5.9%)	18 (5.1%)	15 (3.8%)	0 (0.0%)	0 (0.0%)	0 (0.0%)	0 (0.0%)	.
Small problem	13 (1.3%)	2 (2.0%)	3 (0.9%)	7 (1.8%)	0 (0.0%)	0 (0.0%)	0 (0.0%)	1 (7.1%)	.
Moderate problem	12 (1.2%)	0 (0.0%)	4 (1.1%)	7 (1.8%)	1 (1.9%)	0 (0.0%)	0 (0.0%)	0 (0.0%)	.
Big problem	2 (0.2%)	0 (0.0%)	0 (0.0%)	2 (0.5%)	0 (0.0%)	0 (0.0%)	0 (0.0%)	0 (0.0%)	.

EPIC-26 results - EBRT

Statistic Variable or Category	EBRT (n=1037)	Australia/New Zealand (n=104)	Canada (n=354)	Central Europe (n=439)	United Kingdom (n=53)	Italy (n=24)	Spain (n=47)	USA (n=16)	P Value
Missing	55	3	3	42	1	4		2	.
How big a problem, if any, has weak urine stream been for you during the last 4 weeks?									.
No problem	521 (52.2%)	52 (50.5%)	187 (53.1%)	208 (50.6%)	24 (46.2%)	15 (75.0%)	29 (61.7%)	6 (42.9%)	0.2092
Very small problem	222 (22.2%)	24 (23.3%)	84 (23.9%)	94 (22.9%)	13 (25.0%)	3 (15.0%)	1 (2.1%)	3 (21.4%)	.
Small problem	156 (15.6%)	18 (17.5%)	48 (13.6%)	66 (16.1%)	6 (11.5%)	2 (10.0%)	13 (27.7%)	3 (21.4%)	.
Moderate problem	84 (8.4%)	7 (6.8%)	29 (8.2%)	36 (8.8%)	8 (15.4%)	0 (0.0%)	3 (6.4%)	1 (7.1%)	.
Big problem	16 (1.6%)	2 (1.9%)	4 (1.1%)	7 (1.7%)	1 (1.9%)	0 (0.0%)	1 (2.1%)	1 (7.1%)	.
Missing	38	1	2	28	1	4		2	.
How big a problem, if any, has need to urinate frequently during the day been for you during the last 4 weeks?									.
No problem	467 (46.2%)	40 (38.8%)	157 (44.5%)	204 (48.7%)	16 (30.2%)	9 (40.9%)	36 (76.6%)	5 (35.7%)	0.0047
Very small problem	240 (23.7%)	32 (31.1%)	88 (24.9%)	95 (22.7%)	13 (24.5%)	9 (40.9%)	0 (0.0%)	3 (21.4%)	.
Small problem	170 (16.8%)	21 (20.4%)	58 (16.4%)	69 (16.5%)	12 (22.6%)	2 (9.1%)	5 (10.6%)	3 (21.4%)	.
Moderate problem	107 (10.6%)	9 (8.7%)	38 (10.8%)	41 (9.8%)	11 (20.8%)	2 (9.1%)	4 (8.5%)	2 (14.3%)	.
Big problem	27 (2.7%)	1 (1.0%)	12 (3.4%)	10 (2.4%)	1 (1.9%)	0 (0.0%)	2 (4.3%)	1 (7.1%)	.
Missing	26	1	1	20		2		2	.
Overall how big a problem has your urinary function been for you during the last 4 weeks?									.
No problem	577 (57.5%)	58 (56.9%)	190 (54.4%)	261 (63.0%)	21 (39.6%)	15 (65.2%)	24 (51.1%)	8 (50.0%)	<.0001
Very small problem	220 (21.9%)	29 (28.4%)	86 (24.6%)	79 (19.1%)	17 (32.1%)	5 (21.7%)	0 (0.0%)	4 (25.0%)	.
Small problem	116 (11.6%)	9 (8.8%)	42 (12.0%)	38 (9.2%)	10 (18.9%)	2 (8.7%)	14 (29.8%)	1 (6.3%)	.
Moderate problem	76 (7.6%)	6 (5.9%)	28 (8.0%)	28 (6.8%)	5 (9.4%)	1 (4.3%)	6 (12.8%)	2 (12.5%)	.
Big problem	15 (1.5%)	0 (0.0%)	3 (0.9%)	8 (1.9%)	0 (0.0%)	0 (0.0%)	3 (6.4%)	1 (6.3%)	.
Missing	33	2	5	25		1			.
Bowel									.
n	948	102	349	368	51	16	47	15	0.0046
Mean (SD)	95.6 (9.91)	94.6 (9.50)	95.5 (10.05)	95.9 (9.43)	94.9 (12.29)	95.3 (7.89)	98.5 (6.58)	90.3 (18.28)	.

EPIC-26 results - EBRT

Statistic Variable or Category	EBRT (n=1037)	Australia/New Zealand (n=104)	Canada (n=354)	Central Europe (n=439)	United Kingdom (n=53)	Italy (n=24)	Spain (n=47)	USA (n=16)	P Value
Median (Q1-Q3)	100.0 (95.8 - 100.0)	100.0 (91.7 - 100.0)	100.0 (95.8 - 100.0)	100.0 (95.8 - 100.0)	100.0 (95.8 - 100.0)	97.9 (95.8 - 100.0)	100.0 (100.0 - 100.0)	100.0 (87.5 - 100.0)	.
Min, Max	25.0, 100.0	50.0, 100.0	25.0, 100.0	33.3, 100.0	33.3, 100.0	70.8, 100.0	62.5, 100.0	37.5, 100.0	.
How big a problem, if any, has urgency to have a bowel movement been for you?									.
No problem	843 (83.0%)	79 (76.0%)	288 (82.3%)	360 (84.5%)	41 (77.4%)	15 (75.0%)	47 (100.0%)	13 (81.3%)	0.0411
Very small problem	108 (10.6%)	15 (14.4%)	38 (10.9%)	43 (10.1%)	8 (15.1%)	4 (20.0%)	0 (0.0%)	0 (0.0%)	.
Small problem	35 (3.4%)	7 (6.7%)	15 (4.3%)	9 (2.1%)	2 (3.8%)	0 (0.0%)	0 (0.0%)	2 (12.5%)	.
Moderate problem	22 (2.2%)	3 (2.9%)	6 (1.7%)	11 (2.6%)	1 (1.9%)	1 (5.0%)	0 (0.0%)	0 (0.0%)	.
Big problem	8 (0.8%)	0 (0.0%)	3 (0.9%)	3 (0.7%)	1 (1.9%)	0 (0.0%)	0 (0.0%)	1 (6.3%)	.
Missing	21		4	13		4			.
How big a problem, if any, has increased frequency of bowel movements been for you?									.
No problem	829 (86.9%)	85 (83.3%)	303 (87.1%)	325 (87.1%)	44 (83.0%)	14 (87.5%)	46 (97.9%)	12 (80.0%)	0.2054
Very small problem	74 (7.8%)	11 (10.8%)	32 (9.2%)	25 (6.7%)	5 (9.4%)	1 (6.3%)	0 (0.0%)	0 (0.0%)	.
Small problem	32 (3.4%)	6 (5.9%)	8 (2.3%)	13 (3.5%)	2 (3.8%)	1 (6.3%)	0 (0.0%)	2 (13.3%)	.
Moderate problem	15 (1.6%)	0 (0.0%)	3 (0.9%)	9 (2.4%)	1 (1.9%)	0 (0.0%)	1 (2.1%)	1 (6.7%)	.
Big problem	4 (0.4%)	0 (0.0%)	2 (0.6%)	1 (0.3%)	1 (1.9%)	0 (0.0%)	0 (0.0%)	0 (0.0%)	.
Missing	83	2	6	66		8		1	.
How big a problem, if any, has losing control of your stools been for you?									.
No problem	904 (95.0%)	96 (94.1%)	333 (95.1%)	353 (95.4%)	49 (94.2%)	15 (93.8%)	46 (97.9%)	12 (80.0%)	0.1597
Very small problem	31 (3.3%)	3 (2.9%)	13 (3.7%)	11 (3.0%)	2 (3.8%)	1 (6.3%)	0 (0.0%)	1 (6.7%)	.
Small problem	7 (0.7%)	3 (2.9%)	0 (0.0%)	2 (0.5%)	1 (1.9%)	0 (0.0%)	0 (0.0%)	1 (6.7%)	.
Moderate problem	9 (0.9%)	0 (0.0%)	3 (0.9%)	4 (1.1%)	0 (0.0%)	0 (0.0%)	1 (2.1%)	1 (6.7%)	.
Big problem	1 (0.1%)	0 (0.0%)	1 (0.3%)	0 (0.0%)	0 (0.0%)	0 (0.0%)	0 (0.0%)	0 (0.0%)	.
Missing	85	2	4	69	1	8		1	.
How big a problem, if any, has bloody stools been for you?									.

EPIC-26 results - EBRT

Statistic Variable or Category	EBRT (n=1037)	Australia/New Zealand (n=104)	Canada (n=354)	Central Europe (n=439)	United Kingdom (n=53)	Italy (n=24)	Spain (n=47)	USA (n=16)	P Value
No problem	920 (96.6%)	98 (96.1%)	332 (94.9%)	361 (97.3%)	51 (100.0%)	16 (100.0%)	47 (100.0%)	15 (100.0%)	0.9566
Very small problem	24 (2.5%)	3 (2.9%)	15 (4.3%)	6 (1.6%)	0 (0.0%)	0 (0.0%)	0 (0.0%)	0 (0.0%)	.
Small problem	5 (0.5%)	1 (1.0%)	1 (0.3%)	3 (0.8%)	0 (0.0%)	0 (0.0%)	0 (0.0%)	0 (0.0%)	.
Moderate problem	2 (0.2%)	0 (0.0%)	1 (0.3%)	1 (0.3%)	0 (0.0%)	0 (0.0%)	0 (0.0%)	0 (0.0%)	.
Big problem	1 (0.1%)	0 (0.0%)	1 (0.3%)	0 (0.0%)	0 (0.0%)	0 (0.0%)	0 (0.0%)	0 (0.0%)	.
Missing	85	2	4	68	2	8		1	.
How big a problem, if any, has abdominal/pelvic/rectal pain been for you?									.
No problem	830 (87.2%)	82 (80.4%)	306 (87.4%)	331 (89.2%)	42 (82.4%)	13 (81.3%)	45 (95.7%)	11 (73.3%)	0.0301
Very small problem	74 (7.8%)	12 (11.8%)	32 (9.1%)	20 (5.4%)	5 (9.8%)	3 (18.8%)	0 (0.0%)	2 (13.3%)	.
Small problem	22 (2.3%)	4 (3.9%)	3 (0.9%)	13 (3.5%)	0 (0.0%)	0 (0.0%)	1 (2.1%)	1 (6.7%)	.
Moderate problem	21 (2.2%)	2 (2.0%)	7 (2.0%)	6 (1.6%)	4 (7.8%)	0 (0.0%)	1 (2.1%)	1 (6.7%)	.
Big problem	5 (0.5%)	2 (2.0%)	2 (0.6%)	1 (0.3%)	0 (0.0%)	0 (0.0%)	0 (0.0%)	0 (0.0%)	.
Missing	85	2	4	68	2	8		1	.
Overall, how big a problem have your bowel habits been for you during the last 4 weeks?									.
No problem	849 (82.8%)	82 (79.6%)	291 (82.7%)	353 (81.7%)	46 (86.8%)	19 (86.4%)	45 (95.7%)	13 (81.3%)	0.1699
Very small problem	109 (10.6%)	17 (16.5%)	41 (11.6%)	44 (10.2%)	4 (7.5%)	2 (9.1%)	0 (0.0%)	1 (6.3%)	.
Small problem	29 (2.8%)	3 (2.9%)	6 (1.7%)	17 (3.9%)	1 (1.9%)	1 (4.5%)	0 (0.0%)	1 (6.3%)	.
Moderate problem	31 (3.0%)	1 (1.0%)	10 (2.8%)	16 (3.7%)	2 (3.8%)	0 (0.0%)	2 (4.3%)	0 (0.0%)	.
Big problem	7 (0.7%)	0 (0.0%)	4 (1.1%)	2 (0.5%)	0 (0.0%)	0 (0.0%)	0 (0.0%)	1 (6.3%)	.
Missing	12	1	2	7		2			.
Sexual									.
n	953	99	339	382	51	21	47	14	0.0005
Mean (SD)	49.7 (30.08)	59.1 (29.05)	52.8 (29.67)	45.6 (29.76)	45.9 (30.75)	44.0 (26.36)	45.1 (31.44)	57.8 (34.62)	.
Median (Q1-Q3)	50.0 (20.8 - 79.2)	58.3 (34.7 - 87.5)	54.2 (26.6 - 82.0)	43.0 (16.7 - 70.8)	43.0 (16.7 - 77.8)	50.0 (20.8 - 57.0)	30.5 (16.7 - 83.3)	62.5 (43.0 - 87.5)	.
Min, Max	0.0, 100.0	0.0, 100.0	0.0, 100.0	0.0, 100.0	0.0, 100.0	0.0, 100.0	0.0, 91.7	0.0, 100.0	.

EPIC-26 results - EBRT

Statistic Variable or Category	EBRT (n=1037)	Australia/New Zealand (n=104)	Canada (n=354)	Central Europe (n=439)	United Kingdom (n=53)	Italy (n=24)	Spain (n=47)	USA (n=16)	P Value
How would you rate your ability to have an erection during the last 4 weeks?									.
Very poor to none	260 (26.6%)	16 (15.8%)	68 (19.8%)	132 (33.3%)	16 (30.8%)	6 (27.3%)	20 (42.6%)	2 (13.3%)	<.0001
Poor	179 (18.3%)	13 (12.9%)	77 (22.4%)	63 (15.9%)	10 (19.2%)	6 (27.3%)	7 (14.9%)	3 (20.0%)	.
Fair	238 (24.4%)	32 (31.7%)	80 (23.3%)	97 (24.5%)	11 (21.2%)	7 (31.8%)	7 (14.9%)	4 (26.7%)	.
Good	220 (22.5%)	19 (18.8%)	86 (25.1%)	86 (21.7%)	13 (25.0%)	2 (9.1%)	13 (27.7%)	1 (6.7%)	.
Very good	79 (8.1%)	21 (20.8%)	32 (9.3%)	18 (4.5%)	2 (3.8%)	1 (4.5%)	0 (0.0%)	5 (33.3%)	.
Missing	61	3	11	43	1	2		1	.
How would you rate your ability to reach orgasm (climax) during the last 4 weeks?									.
Very poor to none	242 (25.3%)	12 (12.0%)	64 (18.7%)	124 (32.6%)	16 (32.0%)	4 (20.0%)	20 (42.6%)	2 (13.3%)	<.0001
Poor	162 (17.0%)	11 (11.0%)	72 (21.0%)	59 (15.5%)	7 (14.0%)	5 (25.0%)	6 (12.8%)	2 (13.3%)	.
Fair	216 (22.6%)	24 (24.0%)	83 (24.2%)	84 (22.1%)	11 (22.0%)	7 (35.0%)	3 (6.4%)	4 (26.7%)	.
Good	253 (26.5%)	29 (29.0%)	94 (27.4%)	95 (25.0%)	11 (22.0%)	3 (15.0%)	18 (38.3%)	3 (20.0%)	.
Very good	82 (8.6%)	24 (24.0%)	30 (8.7%)	18 (4.7%)	5 (10.0%)	1 (5.0%)	0 (0.0%)	4 (26.7%)	.
Missing	82	4	11	59	3	4		1	.
How would you describe the usual quality of your erections during the last 4 weeks?									.
None at all	274 (28.2%)	16 (15.8%)	68 (19.9%)	146 (37.3%)	11 (21.2%)	7 (31.8%)	20 (42.6%)	6 (37.5%)	<.0001
Not firm enough for any sexual activity	169 (17.4%)	15 (14.9%)	66 (19.4%)	60 (15.3%)	16 (30.8%)	5 (22.7%)	6 (12.8%)	1 (6.3%)	.
Firm enough for masturbation and foreplay	169 (17.4%)	19 (18.8%)	64 (18.8%)	64 (16.4%)	11 (21.2%)	4 (18.2%)	3 (6.4%)	4 (25.0%)	.
Firm enough for intercourse	358 (36.9%)	51 (50.5%)	143 (41.9%)	121 (30.9%)	14 (26.9%)	6 (27.3%)	18 (38.3%)	5 (31.3%)	.
Missing	67	3	13	48	1	2			.
How would you describe the frequency of your erections during the last 4 weeks?									.
I never had an erection when I wanted one	290 (30.4%)	24 (24.0%)	72 (21.2%)	139 (36.6%)	17 (33.3%)	8 (38.1%)	27 (57.4%)	3 (20.0%)	<.0001

EPIC-26 results - EBRT

Statistic Variable or Category	EBRT (n=1037)	Australia/New Zealand (n=104)	Canada (n=354)	Central Europe (n=439)	United Kingdom (n=53)	Italy (n=24)	Spain (n=47)	USA (n=16)	P Value
I had an erection less than half the time I wanted one	148 (15.5%)	13 (13.0%)	65 (19.2%)	57 (15.0%)	9 (17.6%)	2 (9.5%)	0 (0.0%)	2 (13.3%)	.
I had an erection about half the time I wanted one	135 (14.2%)	11 (11.0%)	56 (16.5%)	53 (13.9%)	4 (7.8%)	4 (19.0%)	4 (8.5%)	3 (20.0%)	.
I had an erection more than half the time I wanted one	122 (12.8%)	26 (26.0%)	41 (12.1%)	43 (11.3%)	6 (11.8%)	2 (9.5%)	2 (4.3%)	2 (13.3%)	.
I had an erection whenever I wanted one	258 (27.1%)	26 (26.0%)	105 (31.0%)	88 (23.2%)	15 (29.4%)	5 (23.8%)	14 (29.8%)	5 (33.3%)	.
Missing	84	4	15	59	2	3		1	.
Overall, how would you rate your ability to function sexually during the last 4 weeks?									.
Very poor	266 (27.6%)	18 (18.0%)	76 (22.6%)	118 (30.1%)	20 (40.0%)	7 (30.4%)	24 (51.1%)	3 (20.0%)	<.0001
Poor	185 (19.2%)	17 (17.0%)	68 (20.2%)	82 (20.9%)	7 (14.0%)	5 (21.7%)	4 (8.5%)	2 (13.3%)	.
Fair	223 (23.2%)	27 (27.0%)	74 (22.0%)	96 (24.5%)	9 (18.0%)	9 (39.1%)	6 (12.8%)	2 (13.3%)	.
Good	211 (21.9%)	24 (24.0%)	79 (23.5%)	81 (20.7%)	10 (20.0%)	1 (4.3%)	12 (25.5%)	4 (26.7%)	.
Very good	78 (8.1%)	14 (14.0%)	39 (11.6%)	15 (3.8%)	4 (8.0%)	1 (4.3%)	1 (2.1%)	4 (26.7%)	.
Missing	74	4	18	47	3	1		1	.
Overall, how big a problem have your sexual functions been for you during the last 4 weeks?									.
No problem	453 (46.5%)	47 (46.5%)	140 (40.8%)	187 (47.7%)	20 (38.5%)	11 (47.8%)	40 (85.1%)	8 (50.0%)	0.0015
Very small problem	125 (12.8%)	11 (10.9%)	44 (12.8%)	55 (14.0%)	8 (15.4%)	6 (26.1%)	0 (0.0%)	1 (6.3%)	.
Small problem	181 (18.6%)	24 (23.8%)	74 (21.6%)	63 (16.1%)	10 (19.2%)	3 (13.0%)	4 (8.5%)	3 (18.8%)	.
Moderate problem	121 (12.4%)	13 (12.9%)	52 (15.2%)	46 (11.7%)	7 (13.5%)	1 (4.3%)	1 (2.1%)	1 (6.3%)	.
Big problem	94 (9.7%)	6 (5.9%)	33 (9.6%)	41 (10.5%)	7 (13.5%)	2 (8.7%)	2 (4.3%)	3 (18.8%)	.
Missing	63	3	11	47	1	1			.
Hormonal									.
n	960	100	350	376	52	20	47	15	0.0012
Mean (SD)	90.9 (13.42)	90.2 (13.08)	93.2 (11.15)	89.8 (15.33)	89.0 (12.37)	91.8 (10.48)	87.3 (13.98)	90.0 (12.82)	.
Median (Q1-Q3)	95.0 (85.0 - 100.0)	95.0 (85.0 - 100.0)	100.0 (90.0 - 100.0)	95.0 (85.0 - 100.0)	91.9 (85.0 - 97.5)	100.0 (83.1 - 100.0)	90.0 (80.0 - 100.0)	95.0 (80.0 - 100.0)	.

EPIC-26 results - EBRT

Statistic Variable or Category	EBRT (n=1037)	Australia/New Zealand (n=104)	Canada (n=354)	Central Europe (n=439)	United Kingdom (n=53)	Italy (n=24)	Spain (n=47)	USA (n=16)	P Value
Min, Max	0.0, 100.0	50.0, 100.0	30.0, 100.0	0.0, 100.0	45.0, 100.0	70.0, 100.0	30.0, 100.0	60.0, 100.0	.
How big a problem during the last 4 weeks, if any, have hot flashes been for you?									.
No problem	895 (91.5%)	89 (89.0%)	339 (96.3%)	346 (88.7%)	45 (84.9%)	19 (95.0%)	41 (87.2%)	16 (100.0%)	0.0353
Very small problem	32 (3.3%)	7 (7.0%)	6 (1.7%)	16 (4.1%)	3 (5.7%)	0 (0.0%)	0 (0.0%)	0 (0.0%)	.
Small problem	29 (3.0%)	1 (1.0%)	5 (1.4%)	16 (4.1%)	3 (5.7%)	1 (5.0%)	3 (6.4%)	0 (0.0%)	.
Moderate problem	15 (1.5%)	3 (3.0%)	1 (0.3%)	7 (1.8%)	2 (3.8%)	0 (0.0%)	2 (4.3%)	0 (0.0%)	.
Big problem	7 (0.7%)	0 (0.0%)	1 (0.3%)	5 (1.3%)	0 (0.0%)	0 (0.0%)	1 (2.1%)	0 (0.0%)	.
Missing	59	4	2	49		4			.
How big a problem during the last 4 weeks, if any, has breast tenderness/enlargement been for you?									.
No problem	874 (96.6%)	94 (96.9%)	343 (98.0%)	307 (94.2%)	51 (100.0%)	18 (94.7%)	46 (97.9%)	15 (100.0%)	0.5615
Very small problem	17 (1.9%)	2 (2.1%)	3 (0.9%)	12 (3.7%)	0 (0.0%)	0 (0.0%)	0 (0.0%)	0 (0.0%)	.
Small problem	7 (0.8%)	1 (1.0%)	2 (0.6%)	2 (0.6%)	0 (0.0%)	1 (5.3%)	1 (2.1%)	0 (0.0%)	.
Moderate problem	6 (0.7%)	0 (0.0%)	2 (0.6%)	4 (1.2%)	0 (0.0%)	0 (0.0%)	0 (0.0%)	0 (0.0%)	.
Big problem	1 (0.1%)	0 (0.0%)	0 (0.0%)	1 (0.3%)	0 (0.0%)	0 (0.0%)	0 (0.0%)	0 (0.0%)	.
Missing	132	7	4	113	2	5		1	.
How big a problem during the last 4 weeks, if any, has feeling depressed been for you?									.
No problem	687 (71.0%)	69 (70.4%)	271 (77.4%)	261 (67.8%)	36 (69.2%)	15 (75.0%)	24 (51.1%)	11 (73.3%)	<.0001
Very small problem	133 (13.8%)	17 (17.3%)	42 (12.0%)	61 (15.8%)	10 (19.2%)	3 (15.0%)	0 (0.0%)	0 (0.0%)	.
Small problem	82 (8.5%)	8 (8.2%)	22 (6.3%)	37 (9.6%)	5 (9.6%)	1 (5.0%)	8 (17.0%)	1 (6.7%)	.
Moderate problem	47 (4.9%)	4 (4.1%)	12 (3.4%)	16 (4.2%)	1 (1.9%)	0 (0.0%)	11 (23.4%)	3 (20.0%)	.
Big problem	18 (1.9%)	0 (0.0%)	3 (0.9%)	10 (2.6%)	0 (0.0%)	1 (5.0%)	4 (8.5%)	0 (0.0%)	.
Missing	70	6	4	54	1	4		1	.
How big a problem during the last 4 weeks, if any, has lack of energy been for you?									.

EPIC-26 results - EBRT

Statistic Variable or Category	EBRT (n=1037)	Australia/New Zealand (n=104)	Canada (n=354)	Central Europe (n=439)	United Kingdom (n=53)	Italy (n=24)	Spain (n=47)	USA (n=16)	P Value
No problem	573 (58.4%)	53 (52.5%)	218 (61.9%)	224 (56.9%)	20 (39.2%)	16 (76.2%)	35 (74.5%)	7 (46.7%)	0.0001
Very small problem	190 (19.4%)	21 (20.8%)	71 (20.2%)	77 (19.5%)	16 (31.4%)	2 (9.5%)	0 (0.0%)	3 (20.0%)	.
Small problem	126 (12.8%)	13 (12.9%)	40 (11.4%)	60 (15.2%)	6 (11.8%)	2 (9.5%)	4 (8.5%)	1 (6.7%)	.
Moderate problem	71 (7.2%)	11 (10.9%)	19 (5.4%)	20 (5.1%)	9 (17.6%)	1 (4.8%)	7 (14.9%)	4 (26.7%)	.
Big problem	21 (2.1%)	3 (3.0%)	4 (1.1%)	13 (3.3%)	0 (0.0%)	0 (0.0%)	1 (2.1%)	0 (0.0%)	.
Missing	56	3	2	45	2	3		1	.
How big a problem during the last 4 weeks, if any, has change in body weight been for you?									.
No problem	806 (82.4%)	77 (74.8%)	297 (84.6%)	318 (81.3%)	39 (78.0%)	15 (71.4%)	46 (97.9%)	14 (93.3%)	0.0278
Very small problem	89 (9.1%)	14 (13.6%)	33 (9.4%)	36 (9.2%)	4 (8.0%)	2 (9.5%)	0 (0.0%)	0 (0.0%)	.
Small problem	46 (4.7%)	7 (6.8%)	12 (3.4%)	16 (4.1%)	6 (12.0%)	4 (19.0%)	0 (0.0%)	1 (6.7%)	.
Moderate problem	31 (3.2%)	5 (4.9%)	7 (2.0%)	17 (4.3%)	1 (2.0%)	0 (0.0%)	1 (2.1%)	0 (0.0%)	.
Big problem	6 (0.6%)	0 (0.0%)	2 (0.6%)	4 (1.0%)	0 (0.0%)	0 (0.0%)	0 (0.0%)	0 (0.0%)	.
Missing	59	1	3	48	3	3		1	.

EPIC-26 results - Brachytherapy

Statistic Variable or Category	Brachytherapy (n=271)	Australia/New Zealand (n=22)	Canada (n=83)	Central Europe (n=89)	United Kingdom (n=30)	Spain (n=41)	USA (n=6)	P Value
Urinary Incontinence								.
n	267	21	81	89	29	41	6	0.7476
Mean (SD)	95.2 (10.16)	94.4 (9.63)	95.1 (9.24)	95.9 (8.11)	97.8 (6.20)	92.6 (16.44)	95.5 (11.02)	.
Median (Q1-Q3)	100.0 (100.0 - 100.0)	100.0 (85.5 - 100.0)	100.0 (91.8 - 100.0)	100.0 (93.8 - 100.0)	100.0 (100.0 - 100.0)	100.0 (100.0 - 100.0)	100.0 (100.0 - 100.0)	.
Min, Max	39.5, 100.0	73.0, 100.0	60.5, 100.0	64.8, 100.0	73.0, 100.0	39.5, 100.0	73.0, 100.0	.
Over the past 4 weeks, how often have you leaked urine?								.
More than once a day	3 (1.1%)	0 (0.0%)	1 (1.2%)	2 (2.2%)	0 (0.0%)	0 (0.0%)	0 (0.0%)	0.4912
About once a day	4 (1.5%)	0 (0.0%)	1 (1.2%)	0 (0.0%)	1 (3.3%)	2 (4.9%)	0 (0.0%)	.
More than once a week	8 (3.0%)	2 (9.1%)	2 (2.4%)	1 (1.1%)	0 (0.0%)	3 (7.3%)	0 (0.0%)	.
About once a week	16 (5.9%)	1 (4.5%)	6 (7.2%)	5 (5.6%)	0 (0.0%)	3 (7.3%)	1 (16.7%)	.
Rarely or never	240 (88.6%)	19 (86.4%)	73 (88.0%)	81 (91.0%)	29 (96.7%)	33 (80.5%)	5 (83.3%)	.
Which of the following best describes your urinary control during the last 4 weeks?								.
No urinary control whatsoever	2 (0.7%)	0 (0.0%)	1 (1.2%)	1 (1.1%)	0 (0.0%)	0 (0.0%)	0 (0.0%)	0.4821
Frequent dribbling	2 (0.7%)	0 (0.0%)	0 (0.0%)	0 (0.0%)	0 (0.0%)	2 (4.9%)	0 (0.0%)	.
Occasional dribbling	53 (19.6%)	6 (27.3%)	18 (21.7%)	18 (20.2%)	4 (13.8%)	6 (14.6%)	1 (16.7%)	.
Total control	213 (78.9%)	16 (72.7%)	64 (77.1%)	70 (78.7%)	25 (86.2%)	33 (80.5%)	5 (83.3%)	.
Missing	1				1			.
How many pads or adult diapers per day did you usually use to control leakage during the last 4 weeks?								.
None	267 (99.3%)	22 (100.0%)	80 (98.8%)	88 (98.9%)	30 (100.0%)	41 (100.0%)	6 (100.0%)	0.9301
1 pad per day	1 (0.4%)	0 (0.0%)	0 (0.0%)	1 (1.1%)	0 (0.0%)	0 (0.0%)	0 (0.0%)	.
3 or more pads per day	1 (0.4%)	0 (0.0%)	1 (1.2%)	0 (0.0%)	0 (0.0%)	0 (0.0%)	0 (0.0%)	.
Missing	2		2					.
How big a problem, if any, has dripping or leaking urine been for you during the last 4 weeks?								.

EPIC-26 results - Brachytherapy

Statistic Variable or Category	Brachytherapy (n=271)	Australia/New Zealand (n=22)	Canada (n=83)	Central Europe (n=89)	United Kingdom (n=30)	Spain (n=41)	USA (n=6)	P Value
No problem	229 (84.8%)	15 (71.4%)	70 (84.3%)	77 (86.5%)	28 (93.3%)	34 (82.9%)	5 (83.3%)	0.0029
Very small problem	27 (10.0%)	6 (28.6%)	10 (12.0%)	9 (10.1%)	2 (6.7%)	0 (0.0%)	0 (0.0%)	.
Small problem	10 (3.7%)	0 (0.0%)	3 (3.6%)	3 (3.4%)	0 (0.0%)	3 (7.3%)	1 (16.7%)	.
Moderate problem	2 (0.7%)	0 (0.0%)	0 (0.0%)	0 (0.0%)	0 (0.0%)	2 (4.9%)	0 (0.0%)	.
Big problem	2 (0.7%)	0 (0.0%)	0 (0.0%)	0 (0.0%)	0 (0.0%)	2 (4.9%)	0 (0.0%)	.
Missing	1	1						.
Urinary Irritative/Obst.								.
n	258	19	83	81	28	41	6	0.5220
Mean (SD)	92.0 (11.28)	89.1 (11.76)	93.5 (7.59)	92.2 (10.15)	93.3 (9.31)	89.3 (18.66)	90.6 (7.65)	.
Median (Q1-Q3)	93.8 (87.5 - 100.0)	93.8 (81.3 - 100.0)	93.8 (87.5 - 100.0)	93.8 (87.5 - 100.0)	96.9 (90.6 - 100.0)	100.0 (81.3 - 100.0)	87.5 (87.5 - 100.0)	.
Min, Max	31.3, 100.0	62.5, 100.0	62.5, 100.0	62.5, 100.0	68.8, 100.0	31.3, 100.0	81.3, 100.0	.
How big a problem, if any, has pain or burning on urination been for you during the last 4 weeks?								.
No problem	237 (90.5%)	18 (85.7%)	78 (94.0%)	75 (92.6%)	27 (90.0%)	33 (80.5%)	6 (100.0%)	0.1064
Very small problem	17 (6.5%)	3 (14.3%)	4 (4.8%)	5 (6.2%)	3 (10.0%)	2 (4.9%)	0 (0.0%)	.
Small problem	5 (1.9%)	0 (0.0%)	1 (1.2%)	1 (1.2%)	0 (0.0%)	3 (7.3%)	0 (0.0%)	.
Moderate problem	2 (0.8%)	0 (0.0%)	0 (0.0%)	0 (0.0%)	0 (0.0%)	2 (4.9%)	0 (0.0%)	.
Big problem	1 (0.4%)	0 (0.0%)	0 (0.0%)	0 (0.0%)	0 (0.0%)	1 (2.4%)	0 (0.0%)	.
Missing	9	1		8				.
How big a problem, if any, has bleeding with urination been for you during the last 4 weeks?								.
No problem	256 (97.3%)	17 (81.0%)	82 (98.8%)	82 (98.8%)	29 (100.0%)	40 (97.6%)	6 (100.0%)	0.0006
Very small problem	4 (1.5%)	2 (9.5%)	1 (1.2%)	1 (1.2%)	0 (0.0%)	0 (0.0%)	0 (0.0%)	.
Small problem	2 (0.8%)	2 (9.5%)	0 (0.0%)	0 (0.0%)	0 (0.0%)	0 (0.0%)	0 (0.0%)	.
Big problem	1 (0.4%)	0 (0.0%)	0 (0.0%)	0 (0.0%)	0 (0.0%)	1 (2.4%)	0 (0.0%)	.
Missing	8	1		6	1			.

EPIC-26 results - Brachytherapy

Statistic Variable or Category	Brachytherapy (n=271)	Australia/New Zealand (n=22)	Canada (n=83)	Central Europe (n=89)	United Kingdom (n=30)	Spain (n=41)	USA (n=6)	P Value
How big a problem, if any, has weak urine stream been for you during the last 4 weeks?								.
No problem	175 (66.5%)	13 (61.9%)	52 (62.7%)	54 (65.9%)	21 (70.0%)	32 (78.0%)	3 (50.0%)	0.0413
Very small problem	58 (22.1%)	6 (28.6%)	25 (30.1%)	18 (22.0%)	7 (23.3%)	0 (0.0%)	2 (33.3%)	.
Small problem	17 (6.5%)	1 (4.8%)	4 (4.8%)	8 (9.8%)	0 (0.0%)	3 (7.3%)	1 (16.7%)	.
Moderate problem	8 (3.0%)	1 (4.8%)	1 (1.2%)	1 (1.2%)	2 (6.7%)	3 (7.3%)	0 (0.0%)	.
Big problem	5 (1.9%)	0 (0.0%)	1 (1.2%)	1 (1.2%)	0 (0.0%)	3 (7.3%)	0 (0.0%)	.
Missing	8	1		7				.
How big a problem, if any, has need to urinate frequently during the day been for you during the last 4 weeks?								.
No problem	163 (62.2%)	9 (45.0%)	51 (61.4%)	49 (59.0%)	17 (58.6%)	34 (82.9%)	3 (50.0%)	0.0056
Very small problem	62 (23.7%)	7 (35.0%)	26 (31.3%)	21 (25.3%)	7 (24.1%)	0 (0.0%)	1 (16.7%)	.
Small problem	23 (8.8%)	3 (15.0%)	5 (6.0%)	7 (8.4%)	4 (13.8%)	2 (4.9%)	2 (33.3%)	.
Moderate problem	12 (4.6%)	1 (5.0%)	1 (1.2%)	6 (7.2%)	1 (3.4%)	3 (7.3%)	0 (0.0%)	.
Big problem	2 (0.8%)	0 (0.0%)	0 (0.0%)	0 (0.0%)	0 (0.0%)	2 (4.9%)	0 (0.0%)	.
Missing	9	2		6	1			.
Overall how big a problem has your urinary function been for you during the last 4 weeks?								.
No problem	199 (73.4%)	13 (59.1%)	60 (72.3%)	72 (80.9%)	22 (73.3%)	28 (68.3%)	4 (66.7%)	<.0001
Very small problem	44 (16.2%)	7 (31.8%)	19 (22.9%)	13 (14.6%)	4 (13.3%)	0 (0.0%)	1 (16.7%)	.
Small problem	17 (6.3%)	2 (9.1%)	4 (4.8%)	2 (2.2%)	3 (10.0%)	5 (12.2%)	1 (16.7%)	.
Moderate problem	8 (3.0%)	0 (0.0%)	0 (0.0%)	2 (2.2%)	1 (3.3%)	5 (12.2%)	0 (0.0%)	.
Big problem	3 (1.1%)	0 (0.0%)	0 (0.0%)	0 (0.0%)	0 (0.0%)	3 (7.3%)	0 (0.0%)	.
Bowel								.
n	263	22	83	81	30	41	6	0.0009
Mean (SD)	96.6 (9.83)	88.8 (16.77)	97.7 (4.97)	98.5 (5.44)	94.9 (12.66)	95.3 (14.53)	98.6 (2.15)	.
Median (Q1-Q3)	100.0 (100.0 - 100.0)	95.8 (83.3 - 100.0)	100.0 (95.8 - 100.0)	100.0 (100.0 - 100.0)	100.0 (95.8 - 100.0)	100.0 (100.0 - 100.0)	100.0 (95.8 - 100.0)	.
Min, Max	20.8, 100.0	41.7, 100.0	70.8, 100.0	66.7, 100.0	41.7, 100.0	20.8, 100.0	95.8, 100.0	.

EPIC-26 results - Brachytherapy

Statistic Variable or Category	Brachytherapy (n=271)	Australia/New Zealand (n=22)	Canada (n=83)	Central Europe (n=89)	United Kingdom (n=30)	Spain (n=41)	USA (n=6)	P Value
How big a problem, if any, has urgency to have a bowel movement been for you?								.
No problem	237 (87.5%)	13 (59.1%)	71 (85.5%)	84 (94.4%)	24 (80.0%)	40 (97.6%)	5 (83.3%)	0.0002
Very small problem	22 (8.1%)	4 (18.2%)	10 (12.0%)	2 (2.2%)	5 (16.7%)	0 (0.0%)	1 (16.7%)	.
Small problem	8 (3.0%)	3 (13.6%)	2 (2.4%)	3 (3.4%)	0 (0.0%)	0 (0.0%)	0 (0.0%)	.
Moderate problem	3 (1.1%)	2 (9.1%)	0 (0.0%)	0 (0.0%)	1 (3.3%)	0 (0.0%)	0 (0.0%)	.
Big problem	1 (0.4%)	0 (0.0%)	0 (0.0%)	0 (0.0%)	0 (0.0%)	1 (2.4%)	0 (0.0%)	.
How big a problem, if any, has increased frequency of bowel movements been for you?								.
No problem	240 (91.3%)	13 (59.1%)	78 (94.0%)	78 (96.3%)	27 (90.0%)	38 (92.7%)	6 (100.0%)	0.0002
Very small problem	13 (4.9%)	5 (22.7%)	5 (6.0%)	1 (1.2%)	2 (6.7%)	0 (0.0%)	0 (0.0%)	.
Small problem	4 (1.5%)	1 (4.5%)	0 (0.0%)	2 (2.5%)	0 (0.0%)	1 (2.4%)	0 (0.0%)	.
Moderate problem	5 (1.9%)	3 (13.6%)	0 (0.0%)	0 (0.0%)	1 (3.3%)	1 (2.4%)	0 (0.0%)	.
Big problem	1 (0.4%)	0 (0.0%)	0 (0.0%)	0 (0.0%)	0 (0.0%)	1 (2.4%)	0 (0.0%)	.
Missing	8			8				.
How big a problem, if any, has losing control of your stools been for you?								.
No problem	252 (95.8%)	17 (77.3%)	82 (98.8%)	80 (98.8%)	28 (93.3%)	39 (95.1%)	6 (100.0%)	0.0019
Very small problem	7 (2.7%)	4 (18.2%)	1 (1.2%)	0 (0.0%)	2 (6.7%)	0 (0.0%)	0 (0.0%)	.
Small problem	2 (0.8%)	1 (4.5%)	0 (0.0%)	1 (1.2%)	0 (0.0%)	0 (0.0%)	0 (0.0%)	.
Moderate problem	1 (0.4%)	0 (0.0%)	0 (0.0%)	0 (0.0%)	0 (0.0%)	1 (2.4%)	0 (0.0%)	.
Big problem	1 (0.4%)	0 (0.0%)	0 (0.0%)	0 (0.0%)	0 (0.0%)	1 (2.4%)	0 (0.0%)	.
Missing	8			8				.
How big a problem, if any, has bloody stools been for you?								.
No problem	254 (96.9%)	20 (90.9%)	82 (98.8%)	80 (98.8%)	27 (93.1%)	39 (95.1%)	6 (100.0%)	0.1514
Very small problem	4 (1.5%)	1 (4.5%)	1 (1.2%)	1 (1.2%)	1 (3.4%)	0 (0.0%)	0 (0.0%)	.
Small problem	3 (1.1%)	1 (4.5%)	0 (0.0%)	0 (0.0%)	0 (0.0%)	2 (4.9%)	0 (0.0%)	.
Moderate problem	1 (0.4%)	0 (0.0%)	0 (0.0%)	0 (0.0%)	1 (3.4%)	0 (0.0%)	0 (0.0%)	.

EPIC-26 results - Brachytherapy

Statistic Variable or Category	Brachytherapy (n=271)	Australia/New Zealand (n=22)	Canada (n=83)	Central Europe (n=89)	United Kingdom (n=30)	Spain (n=41)	USA (n=6)	P Value
Missing	9			8	1			.
How big a problem, if any, has abdominal/pelvic/rectal pain been for you?								.
No problem	230 (87.5%)	16 (72.7%)	72 (86.7%)	77 (93.9%)	24 (82.8%)	36 (87.8%)	5 (83.3%)	0.2458
Very small problem	19 (7.2%)	4 (18.2%)	8 (9.6%)	4 (4.9%)	2 (6.9%)	0 (0.0%)	1 (16.7%)	.
Small problem	8 (3.0%)	1 (4.5%)	2 (2.4%)	0 (0.0%)	2 (6.9%)	3 (7.3%)	0 (0.0%)	.
Moderate problem	3 (1.1%)	1 (4.5%)	1 (1.2%)	0 (0.0%)	0 (0.0%)	1 (2.4%)	0 (0.0%)	.
Big problem	3 (1.1%)	0 (0.0%)	0 (0.0%)	1 (1.2%)	1 (3.4%)	1 (2.4%)	0 (0.0%)	.
Missing	8			7	1			.
Overall, how big a problem have your bowel habits been for you during the last 4 weeks?								.
No problem	246 (91.1%)	16 (76.2%)	76 (91.6%)	84 (94.4%)	26 (86.7%)	38 (92.7%)	6 (100.0%)	0.3743
Very small problem	13 (4.8%)	2 (9.5%)	5 (6.0%)	4 (4.5%)	2 (6.7%)	0 (0.0%)	0 (0.0%)	.
Small problem	7 (2.6%)	2 (9.5%)	2 (2.4%)	1 (1.1%)	1 (3.3%)	1 (2.4%)	0 (0.0%)	.
Moderate problem	3 (1.1%)	1 (4.8%)	0 (0.0%)	0 (0.0%)	1 (3.3%)	1 (2.4%)	0 (0.0%)	.
Big problem	1 (0.4%)	0 (0.0%)	0 (0.0%)	0 (0.0%)	0 (0.0%)	1 (2.4%)	0 (0.0%)	.
Missing	1	1						.
Sexual								.
n	267	22	80	88	30	41	6	0.0066
Mean (SD)	66.1 (27.07)	70.7 (23.67)	68.3 (25.24)	63.2 (25.95)	74.8 (29.08)	56.0 (30.70)	85.9 (18.66)	.
Median (Q1-Q3)	75.0 (45.8 - 87.5)	80.6 (45.8 - 87.5)	75.0 (49.3 - 87.5)	64.6 (44.4 - 83.3)	87.5 (70.8 - 95.8)	66.7 (22.2 - 83.3)	91.7 (79.2 - 100.0)	.
Min, Max	0.0, 100.0	26.3, 100.0	13.8, 100.0	0.0, 100.0	12.5, 100.0	4.2, 87.5	52.8, 100.0	.
How would you rate your ability to have an erection during the last 4 weeks?								.
Very poor to none	31 (11.6%)	0 (0.0%)	7 (8.8%)	10 (11.4%)	4 (13.3%)	10 (24.4%)	0 (0.0%)	0.0260
Poor	33 (12.4%)	5 (22.7%)	10 (12.5%)	9 (10.2%)	3 (10.0%)	6 (14.6%)	0 (0.0%)	.
Fair	61 (22.8%)	6 (27.3%)	17 (21.3%)	24 (27.3%)	5 (16.7%)	8 (19.5%)	1 (16.7%)	.

EPIC-26 results - Brachytherapy

Statistic Variable or Category	Brachytherapy (n=271)	Australia/New Zealand (n=22)	Canada (n=83)	Central Europe (n=89)	United Kingdom (n=30)	Spain (n=41)	USA (n=6)	P Value
Good	103 (38.6%)	6 (27.3%)	31 (38.8%)	37 (42.0%)	10 (33.3%)	17 (41.5%)	2 (33.3%)	.
Very good	39 (14.6%)	5 (22.7%)	15 (18.8%)	8 (9.1%)	8 (26.7%)	0 (0.0%)	3 (50.0%)	.
Missing	4		3	1				.
How would you rate your ability to reach orgasm (climax) during the last 4 weeks?								.
Very poor to none	33 (12.5%)	1 (4.5%)	9 (11.3%)	10 (11.6%)	3 (10.0%)	10 (24.4%)	0 (0.0%)	0.0144
Poor	21 (7.9%)	2 (9.1%)	6 (7.5%)	7 (8.1%)	3 (10.0%)	3 (7.3%)	0 (0.0%)	.
Fair	57 (21.5%)	6 (27.3%)	18 (22.5%)	24 (27.9%)	2 (6.7%)	6 (14.6%)	1 (16.7%)	.
Good	108 (40.8%)	7 (31.8%)	29 (36.3%)	36 (41.9%)	12 (40.0%)	22 (53.7%)	2 (33.3%)	.
Very good	46 (17.4%)	6 (27.3%)	18 (22.5%)	9 (10.5%)	10 (33.3%)	0 (0.0%)	3 (50.0%)	.
Missing	6		3	3				.
How would you describe the usual quality of your erections during the last 4 weeks?								.
None at all	32 (12.0%)	0 (0.0%)	5 (6.3%)	14 (16.1%)	4 (13.3%)	9 (22.0%)	0 (0.0%)	0.1429
Not firm enough for any sexual activity	30 (11.3%)	3 (13.6%)	11 (13.8%)	11 (12.6%)	2 (6.7%)	3 (7.3%)	0 (0.0%)	.
Firm enough for masturbation and foreplay	49 (18.4%)	5 (22.7%)	17 (21.3%)	16 (18.4%)	1 (3.3%)	9 (22.0%)	1 (16.7%)	.
Firm enough for intercourse	155 (58.3%)	14 (63.6%)	47 (58.8%)	46 (52.9%)	23 (76.7%)	20 (48.8%)	5 (83.3%)	.
Missing	5		3	2				.
How would you describe the frequency of your erections during the last 4 weeks?								.
I never had an erection when I wanted one	37 (14.0%)	2 (9.1%)	7 (9.0%)	13 (14.9%)	4 (13.3%)	11 (26.8%)	0 (0.0%)	0.0073
I had an erection less than half the time I wanted one	30 (11.4%)	3 (13.6%)	12 (15.4%)	11 (12.6%)	1 (3.3%)	3 (7.3%)	0 (0.0%)	.
I had an erection about half the time I wanted one	35 (13.3%)	1 (4.5%)	11 (14.1%)	19 (21.8%)	1 (3.3%)	3 (7.3%)	0 (0.0%)	.
I had an erection more than half the time I wanted one	42 (15.9%)	6 (27.3%)	12 (15.4%)	17 (19.5%)	3 (10.0%)	2 (4.9%)	2 (33.3%)	.

EPIC-26 results - Brachytherapy

Statistic Variable or Category	Brachytherapy (n=271)	Australia/New Zealand (n=22)	Canada (n=83)	Central Europe (n=89)	United Kingdom (n=30)	Spain (n=41)	USA (n=6)	P Value
I had an erection whenever I wanted one	120 (45.5%)	10 (45.5%)	36 (46.2%)	27 (31.0%)	21 (70.0%)	22 (53.7%)	4 (66.7%)	.
Missing	7		5	2				.
Overall, how would you rate your ability to function sexually during the last 4 weeks?								.
Very poor	32 (12.1%)	1 (4.5%)	8 (10.0%)	6 (7.0%)	5 (16.7%)	12 (29.3%)	0 (0.0%)	0.0024
Poor	33 (12.5%)	2 (9.1%)	10 (12.5%)	15 (17.4%)	0 (0.0%)	6 (14.6%)	0 (0.0%)	.
Fair	68 (25.7%)	6 (27.3%)	19 (23.8%)	26 (30.2%)	5 (16.7%)	11 (26.8%)	1 (16.7%)	.
Good	91 (34.3%)	8 (36.4%)	27 (33.8%)	31 (36.0%)	11 (36.7%)	12 (29.3%)	2 (33.3%)	.
Very good	41 (15.5%)	5 (22.7%)	16 (20.0%)	8 (9.3%)	9 (30.0%)	0 (0.0%)	3 (50.0%)	.
Missing	6		3	3				.
Overall, how big a problem have your sexual functions been for you during the last 4 weeks?								.
No problem	155 (57.8%)	11 (50.0%)	43 (53.1%)	50 (56.8%)	22 (73.3%)	26 (63.4%)	3 (50.0%)	0.2756
Very small problem	50 (18.7%)	4 (18.2%)	20 (24.7%)	19 (21.6%)	5 (16.7%)	0 (0.0%)	2 (33.3%)	.
Small problem	24 (9.0%)	2 (9.1%)	9 (11.1%)	7 (8.0%)	2 (6.7%)	4 (9.8%)	0 (0.0%)	.
Moderate problem	36 (13.4%)	5 (22.7%)	8 (9.9%)	11 (12.5%)	1 (3.3%)	10 (24.4%)	1 (16.7%)	.
Big problem	3 (1.1%)	0 (0.0%)	1 (1.2%)	1 (1.1%)	0 (0.0%)	1 (2.4%)	0 (0.0%)	.
Missing	3		2	1				.
Hormonal								.
n	267	22	82	86	30	41	6	0.7590
Mean (SD)	91.5 (12.41)	93.2 (10.30)	92.7 (10.52)	92.1 (11.97)	90.7 (13.24)	87.8 (15.57)	87.5 (19.43)	.
Median (Q1-Q3)	100.0 (85.0 - 100.0)	97.5 (90.0 - 100.0)	100.0 (90.0 - 100.0)	100.0 (85.0 - 100.0)	97.5 (85.0 - 100.0)	90.0 (85.0 - 100.0)	95.0 (85.0 - 100.0)	.
Min, Max	40.0, 100.0	60.0, 100.0	60.0, 100.0	40.0, 100.0	45.0, 100.0	45.0, 100.0	50.0, 100.0	.
How big a problem during the last 4 weeks, if any, have hot flashes been for you?								.
No problem	252 (93.7%)	20 (90.9%)	77 (93.9%)	82 (93.2%)	26 (86.7%)	41 (100.0%)	6 (100.0%)	0.6353
Very small problem	5 (1.9%)	1 (4.5%)	2 (2.4%)	2 (2.3%)	0 (0.0%)	0 (0.0%)	0 (0.0%)	.

EPIC-26 results - Brachytherapy

Statistic Variable or Category	Brachytherapy (n=271)	Australia/New Zealand (n=22)	Canada (n=83)	Central Europe (n=89)	United Kingdom (n=30)	Spain (n=41)	USA (n=6)	P Value
Small problem	7 (2.6%)	1 (4.5%)	1 (1.2%)	3 (3.4%)	2 (6.7%)	0 (0.0%)	0 (0.0%)	.
Moderate problem	5 (1.9%)	0 (0.0%)	2 (2.4%)	1 (1.1%)	2 (6.7%)	0 (0.0%)	0 (0.0%)	.
Missing	2		1	1				.
How big a problem during the last 4 weeks, if any, has breast tenderness/enlargement been for you?								.
No problem	258 (98.9%)	22 (100.0%)	81 (98.8%)	79 (98.8%)	30 (100.0%)	41 (100.0%)	5 (83.3%)	<.0001
Very small problem	1 (0.4%)	0 (0.0%)	1 (1.2%)	0 (0.0%)	0 (0.0%)	0 (0.0%)	0 (0.0%)	.
Moderate problem	1 (0.4%)	0 (0.0%)	0 (0.0%)	0 (0.0%)	0 (0.0%)	0 (0.0%)	1 (16.7%)	.
Big problem	1 (0.4%)	0 (0.0%)	0 (0.0%)	1 (1.3%)	0 (0.0%)	0 (0.0%)	0 (0.0%)	.
Missing	10		1	9				.
How big a problem during the last 4 weeks, if any, has feeling depressed been for you?								.
No problem	183 (68.5%)	17 (77.3%)	61 (74.4%)	61 (70.9%)	20 (66.7%)	20 (48.8%)	4 (66.7%)	<.0001
Very small problem	32 (12.0%)	2 (9.1%)	12 (14.6%)	9 (10.5%)	8 (26.7%)	0 (0.0%)	1 (16.7%)	.
Small problem	28 (10.5%)	1 (4.5%)	7 (8.5%)	13 (15.1%)	0 (0.0%)	7 (17.1%)	0 (0.0%)	.
Moderate problem	14 (5.2%)	0 (0.0%)	2 (2.4%)	2 (2.3%)	2 (6.7%)	7 (17.1%)	1 (16.7%)	.
Big problem	10 (3.7%)	2 (9.1%)	0 (0.0%)	1 (1.2%)	0 (0.0%)	7 (17.1%)	0 (0.0%)	.
Missing	4		1	3				.
How big a problem during the last 4 weeks, if any, has lack of energy been for you?								.
No problem	176 (65.7%)	13 (59.1%)	54 (65.9%)	55 (63.2%)	19 (63.3%)	32 (78.0%)	3 (50.0%)	0.0151
Very small problem	41 (15.3%)	7 (31.8%)	14 (17.1%)	16 (18.4%)	3 (10.0%)	0 (0.0%)	1 (16.7%)	.
Small problem	30 (11.2%)	1 (4.5%)	11 (13.4%)	12 (13.8%)	4 (13.3%)	1 (2.4%)	1 (16.7%)	.
Moderate problem	11 (4.1%)	1 (4.5%)	2 (2.4%)	2 (2.3%)	3 (10.0%)	3 (7.3%)	0 (0.0%)	.
Big problem	10 (3.7%)	0 (0.0%)	1 (1.2%)	2 (2.3%)	1 (3.3%)	5 (12.2%)	1 (16.7%)	.
Missing	3		1	2				.

EPIC-26 results - Brachytherapy

Statistic Variable or Category	Brachytherapy (n=271)	Australia/New Zealand (n=22)	Canada (n=83)	Central Europe (n=89)	United Kingdom (n=30)	Spain (n=41)	USA (n=6)	P Value
How big a problem during the last 4 weeks, if any, has change in body weight been for you?								.
No problem	228 (85.4%)	19 (86.4%)	66 (80.5%)	73 (84.9%)	26 (86.7%)	39 (95.1%)	5 (83.3%)	0.5935
Very small problem	21 (7.9%)	3 (13.6%)	7 (8.5%)	9 (10.5%)	1 (3.3%)	0 (0.0%)	1 (16.7%)	.
Small problem	8 (3.0%)	0 (0.0%)	4 (4.9%)	2 (2.3%)	2 (6.7%)	0 (0.0%)	0 (0.0%)	.
Moderate problem	9 (3.4%)	0 (0.0%)	5 (6.1%)	1 (1.2%)	1 (3.3%)	2 (4.9%)	0 (0.0%)	.
Big problem	1 (0.4%)	0 (0.0%)	0 (0.0%)	1 (1.2%)	0 (0.0%)	0 (0.0%)	0 (0.0%)	.
Missing	4		1	3				.

EPIC-26 results - Active Surveillance

Statistic Variable or Category	Active Surveillance (n=1081)	Australia/New Zealand (n=222)	Canada (n=409)	Central Europe (n=116)	United Kingdom (n=47)	Italy (n=39)	USA (n=248)	P Value
Urinary Incontinence								.
n	1035	210	397	111	46	37	234	0.0006
Mean (SD)	91.6 (15.45)	91.7 (14.81)	93.1 (13.50)	86.6 (20.71)	85.9 (18.36)	96.7 (7.28)	91.7 (15.86)	.
Median (Q1-Q3)	100.0 (87.5 - 100.0)	100.0 (85.5 - 100.0)	100.0 (91.8 - 100.0)	100.0 (79.3 - 100.0)	91.8 (73.0 - 100.0)	100.0 (100.0 - 100.0)	100.0 (91.8 - 100.0)	.
Min, Max	8.3, 100.0	22.8, 100.0	22.8, 100.0	8.3, 100.0	39.5, 100.0	75.0, 100.0	8.3, 100.0	.
Over the past 4 weeks, how often have you leaked urine?								.
More than once a day	54 (5.0%)	11 (5.0%)	18 (4.4%)	5 (4.3%)	6 (12.8%)	0 (0.0%)	14 (5.7%)	0.0365
About once a day	43 (4.0%)	13 (5.9%)	12 (2.9%)	8 (7.0%)	3 (6.4%)	0 (0.0%)	7 (2.8%)	.
More than once a week	41 (3.8%)	9 (4.1%)	13 (3.2%)	5 (4.3%)	3 (6.4%)	0 (0.0%)	11 (4.5%)	.
About once a week	70 (6.5%)	17 (7.8%)	30 (7.4%)	6 (5.2%)	6 (12.8%)	0 (0.0%)	11 (4.5%)	.
Rarely or never	865 (80.6%)	169 (77.2%)	334 (82.1%)	91 (79.1%)	29 (61.7%)	39 (100.0%)	203 (82.5%)	.
Missing	8	3	2	1			2	.
Which of the following best describes your urinary control during the last 4 weeks?								.
No urinary control whatsoever	12 (1.1%)	1 (0.5%)	2 (0.5%)	3 (2.6%)	0 (0.0%)	2 (5.1%)	4 (1.6%)	0.0005
Frequent dribbling	36 (3.4%)	6 (2.7%)	11 (2.7%)	7 (6.1%)	3 (6.4%)	1 (2.6%)	8 (3.3%)	.
Occasional dribbling	263 (24.5%)	56 (25.6%)	91 (22.4%)	36 (31.3%)	22 (46.8%)	4 (10.3%)	54 (22.0%)	.
Total control	762 (71.0%)	156 (71.2%)	303 (74.4%)	69 (60.0%)	22 (46.8%)	32 (82.1%)	180 (73.2%)	.
Missing	8	3	2	1			2	.
How many pads or adult diapers per day did you usually use to control leakage during the last 4 weeks?								.
None	1023 (96.1%)	215 (97.3%)	392 (98.2%)	95 (82.6%)	44 (95.7%)	39 (100.0%)	238 (97.5%)	<.0001
1 pad per day	28 (2.6%)	5 (2.3%)	5 (1.3%)	15 (13.0%)	2 (4.3%)	0 (0.0%)	1 (0.4%)	.
2 pads per day	8 (0.8%)	1 (0.5%)	1 (0.3%)	2 (1.7%)	0 (0.0%)	0 (0.0%)	4 (1.6%)	.
3 or more pads per day	5 (0.5%)	0 (0.0%)	1 (0.3%)	3 (2.6%)	0 (0.0%)	0 (0.0%)	1 (0.4%)	.
Missing	17	1	10	1	1		4	.

EPIC-26 results - Active Surveillance

Statistic Variable or Category	Active Surveillance (n=1081)	Australia/New Zealand (n=222)	Canada (n=409)	Central Europe (n=116)	United Kingdom (n=47)	Italy (n=39)	USA (n=248)	P Value
How big a problem, if any, has dripping or leaking urine been for you during the last 4 weeks?								.
No problem	785 (74.5%)	157 (73.7%)	310 (76.2%)	73 (64.6%)	31 (66.0%)	33 (89.2%)	181 (76.7%)	0.0005
Very small problem	173 (16.4%)	42 (19.7%)	68 (16.7%)	17 (15.0%)	8 (17.0%)	3 (8.1%)	35 (14.8%)	.
Small problem	64 (6.1%)	6 (2.8%)	23 (5.7%)	15 (13.3%)	5 (10.6%)	1 (2.7%)	14 (5.9%)	.
Moderate problem	24 (2.3%)	7 (3.3%)	6 (1.5%)	4 (3.5%)	3 (6.4%)	0 (0.0%)	4 (1.7%)	.
Big problem	7 (0.7%)	1 (0.5%)	0 (0.0%)	4 (3.5%)	0 (0.0%)	0 (0.0%)	2 (0.8%)	.
Missing	28	9	2	3		2	12	.
Urinary Irritative/Obst.								.
n	1030	210	404	107	46	37	226	0.0005
Mean (SD)	85.6 (15.33)	84.6 (15.70)	87.8 (14.10)	82.1 (18.33)	79.3 (17.17)	84.1 (16.04)	85.9 (14.42)	.
Median (Q1-Q3)	87.5 (75.0 - 100.0)	87.5 (75.0 - 100.0)	93.8 (81.3 - 100.0)	87.5 (75.0 - 93.8)	81.3 (68.8 - 93.8)	87.5 (81.3 - 93.8)	87.5 (75.0 - 100.0)	.
Min, Max	6.3, 100.0	18.8, 100.0	25.0, 100.0	6.3, 100.0	31.3, 100.0	43.8, 100.0	31.3, 100.0	.
How big a problem, if any, has pain or burning on urination been for you during the last 4 weeks?								.
No problem	900 (86.1%)	186 (88.2%)	353 (86.7%)	88 (80.0%)	40 (85.1%)	30 (81.1%)	203 (87.1%)	0.8548
Very small problem	88 (8.4%)	12 (5.7%)	37 (9.1%)	14 (12.7%)	4 (8.5%)	4 (10.8%)	17 (7.3%)	.
Small problem	33 (3.2%)	7 (3.3%)	10 (2.5%)	4 (3.6%)	1 (2.1%)	2 (5.4%)	9 (3.9%)	.
Moderate problem	20 (1.9%)	5 (2.4%)	5 (1.2%)	4 (3.6%)	2 (4.3%)	1 (2.7%)	3 (1.3%)	.
Big problem	4 (0.4%)	1 (0.5%)	2 (0.5%)	0 (0.0%)	0 (0.0%)	0 (0.0%)	1 (0.4%)	.
Missing	36	11	2	6		2	15	.
How big a problem, if any, has bleeding with urination been for you during the last 4 weeks?								.
No problem	945 (90.5%)	188 (88.3%)	379 (93.3%)	95 (86.4%)	39 (84.8%)	37 (100.0%)	207 (89.2%)	0.0086
Very small problem	61 (5.8%)	18 (8.5%)	19 (4.7%)	6 (5.5%)	2 (4.3%)	0 (0.0%)	16 (6.9%)	.
Small problem	24 (2.3%)	6 (2.8%)	4 (1.0%)	5 (4.5%)	2 (4.3%)	0 (0.0%)	7 (3.0%)	.
Moderate problem	13 (1.2%)	1 (0.5%)	4 (1.0%)	3 (2.7%)	3 (6.5%)	0 (0.0%)	2 (0.9%)	.
Big problem	1 (0.1%)	0 (0.0%)	0 (0.0%)	1 (0.9%)	0 (0.0%)	0 (0.0%)	0 (0.0%)	.

EPIC-26 results - Active Surveillance

Statistic Variable or Category	Active Surveillance (n=1081)	Australia/New Zealand (n=222)	Canada (n=409)	Central Europe (n=116)	United Kingdom (n=47)	Italy (n=39)	USA (n=248)	P Value
Missing	37	9	3	6	1	2	16	.
How big a problem, if any, has weak urine stream been for you during the last 4 weeks?								.
No problem	493 (46.8%)	85 (39.4%)	209 (51.4%)	45 (40.2%)	17 (36.2%)	14 (36.8%)	123 (52.8%)	0.0005
Very small problem	271 (25.7%)	65 (30.1%)	104 (25.6%)	31 (27.7%)	12 (25.5%)	13 (34.2%)	46 (19.7%)	.
Small problem	153 (14.5%)	35 (16.2%)	52 (12.8%)	13 (11.6%)	6 (12.8%)	6 (15.8%)	41 (17.6%)	.
Moderate problem	115 (10.9%)	28 (13.0%)	35 (8.6%)	15 (13.4%)	11 (23.4%)	4 (10.5%)	22 (9.4%)	.
Big problem	21 (2.0%)	3 (1.4%)	7 (1.7%)	8 (7.1%)	1 (2.1%)	1 (2.6%)	1 (0.4%)	.
Missing	28	6	2	4		1	15	.
How big a problem, if any, has need to urinate frequently during the day been for you during the last 4 weeks?								.
No problem	483 (45.6%)	97 (44.5%)	214 (52.3%)	44 (39.3%)	15 (31.9%)	13 (35.1%)	100 (42.2%)	0.0357
Very small problem	244 (23.0%)	52 (23.9%)	95 (23.2%)	26 (23.2%)	10 (21.3%)	11 (29.7%)	50 (21.1%)	.
Small problem	179 (16.9%)	37 (17.0%)	54 (13.2%)	19 (17.0%)	12 (25.5%)	8 (21.6%)	49 (20.7%)	.
Moderate problem	123 (11.6%)	28 (12.8%)	37 (9.0%)	17 (15.2%)	9 (19.1%)	2 (5.4%)	30 (12.7%)	.
Big problem	31 (2.9%)	4 (1.8%)	9 (2.2%)	6 (5.4%)	1 (2.1%)	3 (8.1%)	8 (3.4%)	.
Missing	21	4		4		2	11	.
Overall how big a problem has your urinary function been for you during the last 4 weeks?								.
No problem	571 (53.5%)	105 (48.2%)	236 (57.8%)	54 (48.6%)	16 (34.0%)	22 (56.4%)	138 (56.3%)	0.0063
Very small problem	250 (23.4%)	66 (30.3%)	86 (21.1%)	22 (19.8%)	12 (25.5%)	7 (17.9%)	57 (23.3%)	.
Small problem	139 (13.0%)	25 (11.5%)	55 (13.5%)	15 (13.5%)	9 (19.1%)	5 (12.8%)	30 (12.2%)	.
Moderate problem	92 (8.6%)	20 (9.2%)	26 (6.4%)	16 (14.4%)	10 (21.3%)	5 (12.8%)	15 (6.1%)	.
Big problem	16 (1.5%)	2 (0.9%)	5 (1.2%)	4 (3.6%)	0 (0.0%)	0 (0.0%)	5 (2.0%)	.
Missing	13	4	1	5			3	.
Bowel								.
n	1049	215	408	112	45	33	236	<.0001
Mean (SD)	95.2 (9.79)	94.2 (10.43)	96.1 (9.16)	95.1 (9.16)	92.1 (12.35)	92.6 (11.82)	95.4 (9.52)	.

EPIC-26 results - Active Surveillance

Statistic Variable or Category	Active Surveillance (n=1081)	Australia/New Zealand (n=222)	Canada (n=409)	Central Europe (n=116)	United Kingdom (n=47)	Italy (n=39)	USA (n=248)	P Value
Median (Q1-Q3)	100.0 (95.8 - 100.0)	100.0 (91.7 - 100.0)	100.0 (95.8 - 100.0)	100.0 (91.7 - 100.0)	95.8 (91.7 - 100.0)	100.0 (87.5 - 100.0)	100.0 (95.8 - 100.0)	.
Min, Max	16.7, 100.0	16.7, 100.0	40.0, 100.0	54.2, 100.0	37.5, 100.0	58.3, 100.0	45.8, 100.0	.
How big a problem, if any, has urgency to have a bowel movement been for you?								.
No problem	848 (79.3%)	156 (71.6%)	341 (83.4%)	97 (83.6%)	30 (63.8%)	28 (75.7%)	196 (80.7%)	0.0017
Very small problem	135 (12.6%)	42 (19.3%)	41 (10.0%)	10 (8.6%)	13 (27.7%)	4 (10.8%)	25 (10.3%)	.
Small problem	59 (5.5%)	16 (7.3%)	15 (3.7%)	8 (6.9%)	2 (4.3%)	2 (5.4%)	16 (6.6%)	.
Moderate problem	25 (2.3%)	2 (0.9%)	11 (2.7%)	1 (0.9%)	2 (4.3%)	3 (8.1%)	6 (2.5%)	.
Big problem	3 (0.3%)	2 (0.9%)	1 (0.2%)	0 (0.0%)	0 (0.0%)	0 (0.0%)	0 (0.0%)	.
Missing	11	4				2	5	.
How big a problem, if any, has increased frequency of bowel movements been for you?								.
No problem	889 (84.8%)	180 (84.1%)	352 (86.5%)	93 (83.0%)	35 (77.8%)	30 (90.9%)	199 (84.0%)	0.4198
Very small problem	100 (9.5%)	24 (11.2%)	34 (8.4%)	9 (8.0%)	7 (15.6%)	1 (3.0%)	25 (10.5%)	.
Small problem	37 (3.5%)	6 (2.8%)	14 (3.4%)	7 (6.3%)	1 (2.2%)	0 (0.0%)	9 (3.8%)	.
Moderate problem	18 (1.7%)	2 (0.9%)	5 (1.2%)	3 (2.7%)	2 (4.4%)	2 (6.1%)	4 (1.7%)	.
Big problem	4 (0.4%)	2 (0.9%)	2 (0.5%)	0 (0.0%)	0 (0.0%)	0 (0.0%)	0 (0.0%)	.
Missing	33	8	2	4	2	6	11	.
How big a problem, if any, has losing control of your stools been for you?								.
No problem	995 (95.0%)	201 (94.8%)	388 (95.1%)	108 (96.4%)	42 (89.4%)	33 (100.0%)	223 (94.9%)	0.7996
Very small problem	28 (2.7%)	5 (2.4%)	11 (2.7%)	3 (2.7%)	4 (8.5%)	0 (0.0%)	5 (2.1%)	.
Small problem	17 (1.6%)	3 (1.4%)	6 (1.5%)	1 (0.9%)	1 (2.1%)	0 (0.0%)	6 (2.6%)	.
Moderate problem	4 (0.4%)	2 (0.9%)	1 (0.2%)	0 (0.0%)	0 (0.0%)	0 (0.0%)	1 (0.4%)	.
Big problem	3 (0.3%)	1 (0.5%)	2 (0.5%)	0 (0.0%)	0 (0.0%)	0 (0.0%)	0 (0.0%)	.
Missing	34	10	1	4		6	13	.
How big a problem, if any, has bloody stools been for you?								.

EPIC-26 results - Active Surveillance

Statistic Variable or Category	Active Surveillance (n=1081)	Australia/New Zealand (n=222)	Canada (n=409)	Central Europe (n=116)	United Kingdom (n=47)	Italy (n=39)	USA (n=248)	P Value
No problem	1010 (96.1%)	204 (94.9%)	393 (96.3%)	109 (96.5%)	44 (97.8%)	32 (97.0%)	228 (96.2%)	0.0002
Very small problem	29 (2.8%)	8 (3.7%)	8 (2.0%)	4 (3.5%)	0 (0.0%)	0 (0.0%)	9 (3.8%)	.
Small problem	11 (1.0%)	3 (1.4%)	7 (1.7%)	0 (0.0%)	1 (2.2%)	0 (0.0%)	0 (0.0%)	.
Moderate problem	1 (0.1%)	0 (0.0%)	0 (0.0%)	0 (0.0%)	0 (0.0%)	1 (3.0%)	0 (0.0%)	.
Missing	30	7	1	3	2	6	11	.
How big a problem, if any, has abdominal/pelvic/rectal pain been for you?								.
No problem	902 (85.7%)	169 (77.9%)	368 (90.4%)	90 (79.6%)	37 (78.7%)	24 (72.7%)	214 (90.7%)	<.0001
Very small problem	91 (8.6%)	33 (15.2%)	28 (6.9%)	15 (13.3%)	5 (10.6%)	4 (12.1%)	6 (2.5%)	.
Small problem	42 (4.0%)	11 (5.1%)	7 (1.7%)	6 (5.3%)	3 (6.4%)	4 (12.1%)	11 (4.7%)	.
Moderate problem	15 (1.4%)	2 (0.9%)	4 (1.0%)	2 (1.8%)	2 (4.3%)	1 (3.0%)	4 (1.7%)	.
Big problem	3 (0.3%)	2 (0.9%)	0 (0.0%)	0 (0.0%)	0 (0.0%)	0 (0.0%)	1 (0.4%)	.
Missing	28	5	2	3		6	12	.
Overall, how big a problem have your bowel habits been for you during the last 4 weeks?								.
No problem	875 (81.8%)	165 (76.7%)	356 (87.0%)	93 (80.9%)	33 (70.2%)	30 (76.9%)	198 (80.8%)	0.0285
Very small problem	133 (12.4%)	35 (16.3%)	36 (8.8%)	15 (13.0%)	9 (19.1%)	4 (10.3%)	34 (13.9%)	.
Small problem	39 (3.6%)	9 (4.2%)	11 (2.7%)	5 (4.3%)	2 (4.3%)	2 (5.1%)	10 (4.1%)	.
Moderate problem	19 (1.8%)	4 (1.9%)	6 (1.5%)	1 (0.9%)	3 (6.4%)	3 (7.7%)	2 (0.8%)	.
Big problem	4 (0.4%)	2 (0.9%)	0 (0.0%)	1 (0.9%)	0 (0.0%)	0 (0.0%)	1 (0.4%)	.
Missing	11	7		1			3	.
Sexual								.
n	1014	214	386	108	47	35	224	0.0034
Mean (SD)	64.7 (29.53)	65.3 (30.06)	68.2 (27.07)	58.3 (28.06)	56.2 (28.89)	59.2 (32.70)	63.9 (32.51)	.
Median (Q1-Q3)	70.8 (44.5 - 87.5)	70.8 (46.6 - 87.5)	75.0 (48.7 - 87.5)	61.9 (40.2 - 79.2)	57.0 (32.0 - 79.2)	70.8 (25.0 - 87.5)	70.8 (38.1 - 91.7)	.
Min, Max	0.0, 100.0	0.0, 100.0	0.0, 100.0	0.0, 100.0	4.2, 100.0	0.0, 100.0	0.0, 100.0	.

EPIC-26 results - Active Surveillance

Statistic Variable or Category	Active Surveillance (n=1081)	Australia/New Zealand (n=222)	Canada (n=409)	Central Europe (n=116)	United Kingdom (n=47)	Italy (n=39)	USA (n=248)	P Value
How would you rate your ability to have an erection during the last 4 weeks?								.
Very poor to none	153 (14.6%)	27 (12.5%)	41 (10.3%)	20 (18.3%)	9 (19.1%)	11 (28.9%)	45 (18.9%)	<.0001
Poor	133 (12.7%)	32 (14.8%)	52 (13.1%)	7 (6.4%)	7 (14.9%)	3 (7.9%)	32 (13.4%)	.
Fair	240 (23.0%)	44 (20.4%)	80 (20.2%)	39 (35.8%)	17 (36.2%)	8 (21.1%)	52 (21.8%)	.
Good	324 (31.0%)	67 (31.0%)	154 (38.8%)	31 (28.4%)	11 (23.4%)	11 (28.9%)	50 (21.0%)	.
Very good	195 (18.7%)	46 (21.3%)	70 (17.6%)	12 (11.0%)	3 (6.4%)	5 (13.2%)	59 (24.8%)	.
Missing	36	6	12	7		1	10	.
How would you rate your ability to reach orgasm (climax) during the last 4 weeks?								.
Very poor to none	139 (13.6%)	29 (13.7%)	35 (8.9%)	16 (15.1%)	9 (19.1%)	9 (25.0%)	41 (18.1%)	<.0001
Poor	108 (10.6%)	19 (9.0%)	45 (11.5%)	10 (9.4%)	7 (14.9%)	5 (13.9%)	22 (9.7%)	.
Fair	199 (19.5%)	47 (22.3%)	75 (19.1%)	31 (29.2%)	14 (29.8%)	7 (19.4%)	25 (11.0%)	.
Good	336 (32.9%)	69 (32.7%)	154 (39.2%)	33 (31.1%)	8 (17.0%)	10 (27.8%)	62 (27.3%)	.
Very good	238 (23.3%)	47 (22.3%)	84 (21.4%)	16 (15.1%)	9 (19.1%)	5 (13.9%)	77 (33.9%)	.
Missing	61	11	16	10		3	21	.
How would you describe the usual quality of your erections during the last 4 weeks?								.
None at all	123 (12.0%)	29 (13.6%)	24 (6.2%)	20 (18.2%)	6 (12.8%)	9 (23.7%)	35 (15.1%)	0.0002
Not firm enough for any sexual activity	124 (12.1%)	20 (9.3%)	52 (13.4%)	15 (13.6%)	8 (17.0%)	2 (5.3%)	27 (11.6%)	.
Firm enough for masturbation and foreplay	176 (17.1%)	35 (16.4%)	79 (20.4%)	19 (17.3%)	13 (27.7%)	2 (5.3%)	28 (12.1%)	.
Firm enough for intercourse	605 (58.9%)	130 (60.7%)	232 (59.9%)	56 (50.9%)	20 (42.6%)	25 (65.8%)	142 (61.2%)	.
Missing	53	8	22	6		1	16	.
How would you describe the frequency of your erections during the last 4 weeks?								.
I never had an erection when I wanted one	141 (14.0%)	32 (15.0%)	37 (9.7%)	20 (18.3%)	7 (14.9%)	8 (22.9%)	37 (16.7%)	0.0206

EPIC-26 results - Active Surveillance

Statistic Variable or Category	Active Surveillance (n=1081)	Australia/New Zealand (n=222)	Canada (n=409)	Central Europe (n=116)	United Kingdom (n=47)	Italy (n=39)	USA (n=248)	P Value
I had an erection less than half the time I wanted one	111 (11.0%)	23 (10.7%)	40 (10.5%)	15 (13.8%)	6 (12.8%)	3 (8.6%)	24 (10.9%)	.
I had an erection about half the time I wanted one	135 (13.4%)	24 (11.2%)	51 (13.4%)	23 (21.1%)	9 (19.1%)	2 (5.7%)	26 (11.8%)	.
I had an erection more than half the time I wanted one	181 (18.0%)	46 (21.5%)	66 (17.3%)	23 (21.1%)	7 (14.9%)	5 (14.3%)	34 (15.4%)	.
I had an erection whenever I wanted one	440 (43.7%)	89 (41.6%)	188 (49.2%)	28 (25.7%)	18 (38.3%)	17 (48.6%)	100 (45.2%)	.
Missing	73	8	27	7		4	27	.
Overall, how would you rate your ability to function sexually during the last 4 weeks?								.
Very poor	154 (15.1%)	31 (14.4%)	42 (10.8%)	18 (16.7%)	9 (19.6%)	10 (28.6%)	44 (19.6%)	0.0001
Poor	123 (12.1%)	29 (13.4%)	47 (12.1%)	14 (13.0%)	8 (17.4%)	1 (2.9%)	24 (10.7%)	.
Fair	226 (22.2%)	42 (19.4%)	77 (19.7%)	37 (34.3%)	14 (30.4%)	8 (22.9%)	48 (21.3%)	.
Good	318 (31.2%)	71 (32.9%)	146 (37.4%)	25 (23.1%)	12 (26.1%)	12 (34.3%)	52 (23.1%)	.
Very good	199 (19.5%)	43 (19.9%)	78 (20.0%)	14 (13.0%)	3 (6.5%)	4 (11.4%)	57 (25.3%)	.
Missing	61	6	19	8	1	4	23	.
Overall, how big a problem have your sexual functions been for you during the last 4 weeks?								.
No problem	514 (50.3%)	116 (54.0%)	216 (55.5%)	37 (33.3%)	18 (38.3%)	21 (60.0%)	106 (47.3%)	0.0004
Very small problem	150 (14.7%)	32 (14.9%)	49 (12.6%)	25 (22.5%)	10 (21.3%)	4 (11.4%)	30 (13.4%)	.
Small problem	171 (16.7%)	29 (13.5%)	64 (16.5%)	26 (23.4%)	9 (19.1%)	1 (2.9%)	42 (18.8%)	.
Moderate problem	105 (10.3%)	23 (10.7%)	33 (8.5%)	20 (18.0%)	4 (8.5%)	3 (8.6%)	22 (9.8%)	.
Big problem	81 (7.9%)	15 (7.0%)	27 (6.9%)	3 (2.7%)	6 (12.8%)	6 (17.1%)	24 (10.7%)	.
Missing	60	7	20	5		4	24	.
Hormonal								.
n	1038	213	406	113	47	30	229	<.0001
Mean (SD)	92.8 (10.72)	93.3 (9.18)	94.9 (8.61)	87.8 (13.78)	92.9 (7.85)	92.0 (14.83)	91.0 (12.45)	.
Median (Q1-Q3)	100.0 (90.0 - 100.0)	95.0 (90.0 - 100.0)	100.0 (95.0 - 100.0)	90.0 (80.0 - 100.0)	95.0 (90.0 - 100.0)	100.0 (90.0 - 100.0)	95.0 (87.5 - 100.0)	.
Min, Max	37.5, 100.0	60.0, 100.0	50.0, 100.0	40.0, 100.0	70.0, 100.0	40.0, 100.0	37.5, 100.0	.

EPIC-26 results - Active Surveillance

Statistic Variable or Category	Active Surveillance (n=1081)	Australia/New Zealand (n=222)	Canada (n=409)	Central Europe (n=116)	United Kingdom (n=47)	Italy (n=39)	USA (n=248)	P Value
How big a problem during the last 4 weeks, if any, have hot flashes been for you?								.
No problem	978 (94.1%)	200 (93.9%)	388 (96.0%)	93 (81.6%)	45 (95.7%)	31 (100.0%)	221 (96.1%)	0.0010
Very small problem	34 (3.3%)	5 (2.3%)	11 (2.7%)	13 (11.4%)	2 (4.3%)	0 (0.0%)	3 (1.3%)	.
Small problem	20 (1.9%)	6 (2.8%)	4 (1.0%)	6 (5.3%)	0 (0.0%)	0 (0.0%)	4 (1.7%)	.
Moderate problem	4 (0.4%)	1 (0.5%)	1 (0.2%)	1 (0.9%)	0 (0.0%)	0 (0.0%)	1 (0.4%)	.
Big problem	3 (0.3%)	1 (0.5%)	0 (0.0%)	1 (0.9%)	0 (0.0%)	0 (0.0%)	1 (0.4%)	.
Missing	42	9	5	2		8	18	.
How big a problem during the last 4 weeks, if any, has breast tenderness/enlargement been for you?								.
No problem	1013 (98.7%)	209 (99.5%)	404 (99.8%)	104 (96.3%)	47 (100.0%)	28 (93.3%)	221 (97.8%)	0.0015
Very small problem	5 (0.5%)	0 (0.0%)	1 (0.2%)	3 (2.8%)	0 (0.0%)	0 (0.0%)	1 (0.4%)	.
Small problem	5 (0.5%)	1 (0.5%)	0 (0.0%)	1 (0.9%)	0 (0.0%)	1 (3.3%)	2 (0.9%)	.
Moderate problem	3 (0.3%)	0 (0.0%)	0 (0.0%)	0 (0.0%)	0 (0.0%)	1 (3.3%)	2 (0.9%)	.
Missing	55	12	4	8		9	22	.
How big a problem during the last 4 weeks, if any, has feeling depressed been for you?								.
No problem	784 (75.2%)	167 (78.4%)	332 (82.0%)	66 (58.4%)	34 (72.3%)	20 (64.5%)	165 (70.8%)	0.0013
Very small problem	137 (13.1%)	25 (11.7%)	45 (11.1%)	21 (18.6%)	8 (17.0%)	5 (16.1%)	33 (14.2%)	.
Small problem	76 (7.3%)	14 (6.6%)	20 (4.9%)	16 (14.2%)	2 (4.3%)	3 (9.7%)	21 (9.0%)	.
Moderate problem	38 (3.6%)	5 (2.3%)	7 (1.7%)	9 (8.0%)	3 (6.4%)	3 (9.7%)	11 (4.7%)	.
Big problem	7 (0.7%)	2 (0.9%)	1 (0.2%)	1 (0.9%)	0 (0.0%)	0 (0.0%)	3 (1.3%)	.
Missing	39	9	4	3		8	15	.
How big a problem during the last 4 weeks, if any, has lack of energy been for you?								.
No problem	631 (60.4%)	127 (58.5%)	277 (68.4%)	53 (47.3%)	25 (53.2%)	20 (64.5%)	129 (55.4%)	<.0001
Very small problem	214 (20.5%)	48 (22.1%)	77 (19.0%)	25 (22.3%)	15 (31.9%)	4 (12.9%)	45 (19.3%)	.

EPIC-26 results - Active Surveillance

Statistic Variable or Category	Active Surveillance (n=1081)	Australia/New Zealand (n=222)	Canada (n=409)	Central Europe (n=116)	United Kingdom (n=47)	Italy (n=39)	USA (n=248)	P Value
Small problem	128 (12.2%)	31 (14.3%)	36 (8.9%)	18 (16.1%)	4 (8.5%)	2 (6.5%)	37 (15.9%)	.
Moderate problem	56 (5.4%)	10 (4.6%)	10 (2.5%)	15 (13.4%)	2 (4.3%)	5 (16.1%)	14 (6.0%)	.
Big problem	16 (1.5%)	1 (0.5%)	5 (1.2%)	1 (0.9%)	1 (2.1%)	0 (0.0%)	8 (3.4%)	.
Missing	36	5	4	4		8	15	.
How big a problem during the last 4 weeks, if any, has change in body weight been for you?								.
No problem	881 (84.5%)	181 (83.8%)	353 (87.2%)	89 (79.5%)	37 (82.2%)	29 (96.7%)	192 (82.1%)	0.1859
Very small problem	91 (8.7%)	23 (10.6%)	33 (8.1%)	11 (9.8%)	5 (11.1%)	0 (0.0%)	19 (8.1%)	.
Small problem	48 (4.6%)	7 (3.2%)	15 (3.7%)	7 (6.3%)	3 (6.7%)	0 (0.0%)	16 (6.8%)	.
Moderate problem	16 (1.5%)	3 (1.4%)	3 (0.7%)	4 (3.6%)	0 (0.0%)	0 (0.0%)	6 (2.6%)	.
Big problem	6 (0.6%)	2 (0.9%)	1 (0.2%)	1 (0.9%)	0 (0.0%)	1 (3.3%)	1 (0.4%)	.
Missing	39	6	4	4	2	9	14	.

Characteristics of patients diagnosed as clinically localized or locally advanced.

Statistic Variable or Category	TNGR Global (n=13483)	Clinically Localized (n=12742)	Locally Advanced (n=566)	Unknown (n=175)	P Value
Urinary Incontinence					.
n	12855	1154	196	200	0.0563
Mean (SD)	92.8 (13.99)	90.4 (16.78)	88.4 (17.86)	89.5 (16.02)	.
Median (Q1-Q3)	100.0 (91.8 - 100.0)	100.0 (85.5 - 100.0)	100.0 (74.0 - 100.0)	100.0 (79.3 - 100.0)	.
Min, Max	0.0, 100.0	0.0, 100.0	22.8, 100.0	14.5, 100.0	.
Over the past 4 weeks, how often have you leaked urine?					.
More than once a day	515 (3.9%)	467 (3.7%)	36 (6.4%)	12 (6.9%)	0.0001
About once a day	456 (3.4%)	429 (3.4%)	20 (3.6%)	7 (4.0%)	.
More than once a week	392 (2.9%)	373 (3.0%)	13 (2.3%)	6 (3.4%)	.
About once a week	547 (4.1%)	508 (4.0%)	22 (3.9%)	17 (9.7%)	.
Rarely or never	11432 (85.7%)	10831 (85.9%)	468 (83.7%)	133 (76.0%)	.
Missing	141	134	7		.
Which of the following best describes your urinary control during the last 4 weeks?					.
No urinary control whatsoever	223 (1.7%)	207 (1.6%)	14 (2.5%)	2 (1.2%)	0.0016
Frequent dribbling	355 (2.7%)	321 (2.5%)	29 (5.2%)	5 (2.9%)	.
Occasional dribbling	2957 (22.1%)	2792 (22.1%)	116 (20.7%)	49 (28.3%)	.
Total control	9825 (73.5%)	9307 (73.7%)	401 (71.6%)	117 (67.6%)	.
Missing	123	115	6	2	.
How many pads or adult diapers per day did you usually use to control leakage during the last 4 weeks?					.
None	12969 (97.1%)	12266 (97.1%)	530 (95.2%)	173 (100.0%)	0.0012
1 pad per day	285 (2.1%)	270 (2.1%)	15 (2.7%)	0 (0.0%)	.
2 pads per day	64 (0.5%)	58 (0.5%)	6 (1.1%)	0 (0.0%)	.
3 or more pads per day	40 (0.3%)	34 (0.3%)	6 (1.1%)	0 (0.0%)	.
Missing	125	114	9	2	.
How big a problem, if any, has dripping or leaking urine been for you during the last 4 weeks?					.
No problem	10192 (78.0%)	9656 (78.2%)	413 (75.5%)	123 (71.9%)	<.0001
Very small problem	1915 (14.7%)	1812 (14.7%)	74 (13.5%)	29 (17.0%)	.
Small problem	603 (4.6%)	562 (4.6%)	27 (4.9%)	14 (8.2%)	.
Moderate problem	271 (2.1%)	245 (2.0%)	22 (4.0%)	4 (2.3%)	.
Big problem	79 (0.6%)	67 (0.5%)	11 (2.0%)	1 (0.6%)	.
Missing	423	400	19	4	.

Statistic Variable or Category	TNGR Global (n=13483)	Clinically Localized (n=12742)	Locally Advanced (n=566)	Unknown (n=175)	P Value
Urinary Irritative/Obst.					.
n	12765	1164	193	196	<0.0001
Mean (SD)	86.0 (15.51)	85.7 (15.43)	81.4 (17.57)	83.3 (16.69)	.
Median (Q1-Q3)	87.5 (75.0 - 100.0)	87.5 (75.0 - 100.0)	87.5 (68.8 - 93.8)	87.5 (75.0 - 93.8)	.
Min, Max	0.0, 100.0	18.8, 100.0	31.3, 100.0	12.5, 100.0	.
How big a problem, if any, has pain or burning on urination been for you during the last 4 weeks?					.
No problem	11299 (86.9%)	10709 (87.1%)	445 (82.3%)	145 (84.3%)	0.0002
Very small problem	1011 (7.8%)	951 (7.7%)	43 (7.9%)	17 (9.9%)	.
Small problem	385 (3.0%)	356 (2.9%)	24 (4.4%)	5 (2.9%)	.
Moderate problem	225 (1.7%)	198 (1.6%)	22 (4.1%)	5 (2.9%)	.
Big problem	89 (0.7%)	82 (0.7%)	7 (1.3%)	0 (0.0%)	.
Missing	474	446	25	3	.
How big a problem, if any, has bleeding with urination been for you during the last 4 weeks?					.
No problem	12176 (94.1%)	11520 (94.2%)	498 (93.1%)	158 (92.4%)	0.1719
Very small problem	456 (3.5%)	426 (3.5%)	20 (3.7%)	10 (5.8%)	.
Small problem	183 (1.4%)	173 (1.4%)	9 (1.7%)	1 (0.6%)	.
Moderate problem	95 (0.7%)	89 (0.7%)	4 (0.7%)	2 (1.2%)	.
Big problem	29 (0.2%)	25 (0.2%)	4 (0.7%)	0 (0.0%)	.
Missing	544	509	31	4	.
How big a problem, if any, has weak urine stream been for you during the last 4 weeks?					.
No problem	6361 (48.5%)	6055 (48.9%)	235 (43.2%)	71 (40.6%)	0.0006
Very small problem	3194 (24.4%)	3026 (24.4%)	120 (22.1%)	48 (27.4%)	.
Small problem	1930 (14.7%)	1810 (14.6%)	90 (16.5%)	30 (17.1%)	.
Moderate problem	1244 (9.5%)	1153 (9.3%)	72 (13.2%)	19 (10.9%)	.
Big problem	384 (2.9%)	350 (2.8%)	27 (5.0%)	7 (4.0%)	.
Missing	370	348	22		.
How big a problem, if any, has need to urinate frequently during the day been for you during the last 4 weeks?					.
No problem	6043 (45.8%)	5751 (46.2%)	226 (40.9%)	66 (37.7%)	0.0029
Very small problem	2985 (22.6%)	2833 (22.7%)	114 (20.6%)	38 (21.7%)	.
Small problem	2108 (16.0%)	1972 (15.8%)	99 (17.9%)	37 (21.1%)	.
Moderate problem	1576 (12.0%)	1465 (11.8%)	86 (15.6%)	25 (14.3%)	.
Big problem	471 (3.6%)	434 (3.5%)	28 (5.1%)	9 (5.1%)	.
Missing	300	287	13		.

Statistic Variable or Category	TNGR Global (n=13483)	Clinically Localized (n=12742)	Locally Advanced (n=566)	Unknown (n=175)	P Value
Overall how big a problem has your urinary function been for you during the last 4 weeks?					.
No problem	7698 (58.2%)	7327 (58.6%)	287 (52.2%)	84 (48.3%)	0.0001
Very small problem	2780 (21.0%)	2630 (21.0%)	110 (20.0%)	40 (23.0%)	.
Small problem	1449 (10.9%)	1348 (10.8%)	73 (13.3%)	28 (16.1%)	.
Moderate problem	1031 (7.8%)	953 (7.6%)	59 (10.7%)	19 (10.9%)	.
Big problem	276 (2.1%)	252 (2.0%)	21 (3.8%)	3 (1.7%)	.
Missing	249	232	16	1	.
Bowel					.
n	12850	1179	195	199	0.0100
Mean (SD)	95.5 (9.84)	94.7 (10.77)	93.8 (11.83)	92.5 (12.04)	.
Median (Q1-Q3)	100.0 (95.8 - 100.0)	100.0 (95.8 - 100.0)	100.0 (91.7 - 100.0)	100.0 (87.5 - 100.0)	.
Min, Max	8.3, 100.0	20.8, 100.0	20.8, 100.0	37.5, 100.0	.
How big a problem, if any, has urgency to have a bowel movement been for you?					.
No problem	11159 (83.8%)	10566 (83.9%)	463 (83.9%)	130 (74.7%)	0.0071
Very small problem	1225 (9.2%)	1151 (9.1%)	44 (8.0%)	30 (17.2%)	.
Small problem	547 (4.1%)	516 (4.1%)	22 (4.0%)	9 (5.2%)	.
Moderate problem	308 (2.3%)	288 (2.3%)	16 (2.9%)	4 (2.3%)	.
Big problem	77 (0.6%)	69 (0.5%)	7 (1.3%)	1 (0.6%)	.
Missing	167	152	14	1	.
How big a problem, if any, has increased frequency of bowel movements been for you?					.
No problem	11185 (86.7%)	10601 (86.9%)	441 (84.2%)	143 (82.7%)	0.0097
Very small problem	1010 (7.8%)	936 (7.7%)	50 (9.5%)	24 (13.9%)	.
Small problem	431 (3.3%)	413 (3.4%)	14 (2.7%)	4 (2.3%)	.
Moderate problem	216 (1.7%)	200 (1.6%)	15 (2.9%)	1 (0.6%)	.
Big problem	52 (0.4%)	47 (0.4%)	4 (0.8%)	1 (0.6%)	.
Missing	589	545	42	2	.
How big a problem, if any, has losing control of your stools been for you?					.
No problem	12345 (96.0%)	11682 (96.1%)	506 (96.2%)	157 (91.3%)	0.0048
Very small problem	330 (2.6%)	303 (2.5%)	14 (2.7%)	13 (7.6%)	.
Small problem	113 (0.9%)	109 (0.9%)	3 (0.6%)	1 (0.6%)	.
Moderate problem	46 (0.4%)	43 (0.4%)	3 (0.6%)	0 (0.0%)	.
Big problem	22 (0.2%)	21 (0.2%)	0 (0.0%)	1 (0.6%)	.
Missing	627	584	40	3	.

Statistic Variable or Category	TNGR Global (n=13483)	Clinically Localized (n=12742)	Locally Advanced (n=566)	Unknown (n=175)	P Value
How big a problem, if any, has bloody stools been for you?					.
No problem	12445 (96.7%)	11769 (96.7%)	510 (97.5%)	166 (96.0%)	0.0049
Very small problem	291 (2.3%)	279 (2.3%)	5 (1.0%)	7 (4.0%)	.
Small problem	95 (0.7%)	91 (0.7%)	4 (0.8%)	0 (0.0%)	.
Moderate problem	28 (0.2%)	26 (0.2%)	2 (0.4%)	0 (0.0%)	.
Big problem	6 (0.0%)	4 (0.0%)	2 (0.4%)	0 (0.0%)	.
Missing	618	573	43	2	.
How big a problem, if any, has abdominal/pelvic/rectal pain been for you?					.
No problem	10960 (85.0%)	10382 (85.2%)	430 (81.0%)	148 (85.5%)	0.0057
Very small problem	1127 (8.7%)	1051 (8.6%)	62 (11.7%)	14 (8.1%)	.
Small problem	463 (3.6%)	436 (3.6%)	19 (3.6%)	8 (4.6%)	.
Moderate problem	268 (2.1%)	256 (2.1%)	11 (2.1%)	1 (0.6%)	.
Big problem	76 (0.6%)	65 (0.5%)	9 (1.7%)	2 (1.2%)	.
Missing	589	552	35	2	.
Overall, how big a problem have your bowel habits been for you during the last 4 weeks?					.
No problem	11200 (83.9%)	10608 (84.1%)	461 (82.2%)	131 (75.7%)	0.0018
Very small problem	1324 (9.9%)	1234 (9.8%)	60 (10.7%)	30 (17.3%)	.
Small problem	472 (3.5%)	449 (3.6%)	14 (2.5%)	9 (5.2%)	.
Moderate problem	273 (2.0%)	250 (2.0%)	20 (3.6%)	3 (1.7%)	.
Big problem	82 (0.6%)	76 (0.6%)	6 (1.1%)	0 (0.0%)	.
Missing	132	125	5	2	.
Sexual					.
n	12575	1158	188	200	<0.0001
Mean (SD)	62.0 (29.34)	59.2 (30.99)	41.0 (29.75)	53.3 (31.77)	.
Median (Q1-Q3)	66.7 (38.8 - 87.5)	62.5 (32.0 - 87.5)	34.7 (16.7 - 63.0)	59.0 (20.8 - 83.3)	.
Min, Max	0.0, 100.0	0.0, 100.0	0.0, 100.0	0.0, 100.0	.
How would you rate your ability to have an erection during the last 4 weeks?					.
Very poor to none	1834 (14.3%)	1684 (13.9%)	121 (22.2%)	29 (17.2%)	<.0001
Poor	1873 (14.6%)	1753 (14.4%)	102 (18.8%)	18 (10.7%)	.
Fair	3204 (24.9%)	3042 (25.1%)	122 (22.4%)	40 (23.7%)	.
Good	3844 (29.9%)	3654 (30.1%)	140 (25.7%)	50 (29.6%)	.
Very good	2101 (16.3%)	2010 (16.6%)	59 (10.8%)	32 (18.9%)	.
Missing	627	599	22	6	.

Statistic Variable or Category	TNGR Global (n=13483)	Clinically Localized (n=12742)	Locally Advanced (n=566)	Unknown (n=175)	P Value
How would you rate your ability to reach orgasm (climax) during the last 4 weeks?					.
Very poor to none	1708 (13.5%)	1574 (13.2%)	108 (20.2%)	26 (15.3%)	<.0001
Poor	1552 (12.3%)	1455 (12.2%)	82 (15.3%)	15 (8.8%)	.
Fair	2763 (21.9%)	2611 (21.9%)	108 (20.2%)	44 (25.9%)	.
Good	4209 (33.3%)	3996 (33.5%)	166 (31.0%)	47 (27.6%)	.
Very good	2397 (19.0%)	2288 (19.2%)	71 (13.3%)	38 (22.4%)	.
Missing	854	818	31	5	.
How would you describe the usual quality of your erections during the last 4 weeks?					.
None at all	1869 (14.7%)	1720 (14.3%)	126 (23.2%)	23 (13.5%)	<.0001
Not firm enough for any sexual activity	1655 (13.0%)	1567 (13.0%)	68 (12.5%)	20 (11.8%)	.
Firm enough for masturbation and foreplay	2360 (18.5%)	2210 (18.4%)	111 (20.5%)	39 (22.9%)	.
Firm enough for intercourse	6855 (53.8%)	6530 (54.3%)	237 (43.7%)	88 (51.8%)	.
Missing	744	715	24	5	.
How would you describe the frequency of your erections during the last 4 weeks?					.
I never had an erection when I wanted one	1952 (15.7%)	1795 (15.3%)	130 (24.2%)	27 (16.3%)	<.0001
I had an erection less than half the time I wanted one	1741 (14.0%)	1635 (13.9%)	86 (16.0%)	20 (12.0%)	.
I had an erection about half the time I wanted one	1807 (14.5%)	1704 (14.5%)	80 (14.9%)	23 (13.9%)	.
I had an erection more than half the time I wanted one	2038 (16.3%)	1934 (16.4%)	70 (13.0%)	34 (20.5%)	.
I had an erection whenever I wanted one	4931 (39.5%)	4697 (39.9%)	172 (32.0%)	62 (37.3%)	.
Missing	1014	977	28	9	.
Overall, how would you rate your ability to function sexually during the last 4 weeks?					.
No problem	1956 (15.4%)	1806 (15.1%)	119 (22.0%)	31 (18.3%)	<.0001
Very small problem	1937 (15.3%)	1811 (15.1%)	106 (19.6%)	20 (11.8%)	.
Small problem	3058 (24.1%)	2900 (24.2%)	117 (21.7%)	41 (24.3%)	.
Moderate problem	3865 (30.5%)	3679 (30.7%)	141 (26.1%)	45 (26.6%)	.
Big problem	1876 (14.8%)	1787 (14.9%)	57 (10.6%)	32 (18.9%)	.
Missing	791	759	26	6	.

Statistic Variable or Category	TNGR Global (n=13483)	Clinically Localized (n=12742)	Locally Advanced (n=566)	Unknown (n=175)	P Value
Overall, how big a problem have your sexual functions been for you during the last 4 weeks?					.
No problem	6212 (48.9%)	5893 (49.1%)	247 (45.3%)	72 (42.6%)	0.0812
Very small problem	2018 (15.9%)	1887 (15.7%)	93 (17.1%)	38 (22.5%)	.
Small problem	2058 (16.2%)	1949 (16.2%)	86 (15.8%)	23 (13.6%)	.
Moderate problem	1605 (12.6%)	1500 (12.5%)	84 (15.4%)	21 (12.4%)	.
Big problem	823 (6.5%)	773 (6.4%)	35 (6.4%)	15 (8.9%)	.
Missing	767	740	21	6	.
Hormonal					.
n	12664	1175	197	200	0.6720
Mean (SD)	90.2 (13.55)	90.1 (13.31)	86.4 (16.31)	87.8 (15.39)	.
Median (Q1-Q3)	95.0 (85.0 - 100.0)	95.0 (85.0 - 100.0)	90.0 (80.0 - 100.0)	93.8 (85.0 - 100.0)	.
Min, Max	0.0, 100.0	20.0, 100.0	25.0, 100.0	25.0, 100.0	.
How big a problem during the last 4 weeks, if any, have hot flashes been for you?					.
No problem	11460 (89.5%)	10849 (89.6%)	452 (85.8%)	159 (93.0%)	0.0011
Very small problem	669 (5.2%)	636 (5.3%)	29 (5.5%)	4 (2.3%)	.
Small problem	357 (2.8%)	327 (2.7%)	29 (5.5%)	1 (0.6%)	.
Moderate problem	237 (1.9%)	220 (1.8%)	11 (2.1%)	6 (3.5%)	.
Big problem	83 (0.6%)	76 (0.6%)	6 (1.1%)	1 (0.6%)	.
Missing	677	634	39	4	.
How big a problem during the last 4 weeks, if any, has breast tenderness/enlargement been for you?					.
No problem	11704 (97.6%)	11060 (97.6%)	478 (97.4%)	166 (97.1%)	0.7825
Very small problem	156 (1.3%)	146 (1.3%)	6 (1.2%)	4 (2.3%)	.
Small problem	72 (0.6%)	68 (0.6%)	3 (0.6%)	1 (0.6%)	.
Moderate problem	49 (0.4%)	45 (0.4%)	4 (0.8%)	0 (0.0%)	.
Big problem	10 (0.1%)	10 (0.1%)	0 (0.0%)	0 (0.0%)	.
Missing	1492	1413	75	4	.
How big a problem during the last 4 weeks, if any, has feeling depressed been for you?					.
No problem	8364 (65.7%)	7906 (65.7%)	353 (67.4%)	105 (61.8%)	0.0260
Very small problem	1929 (15.2%)	1828 (15.2%)	69 (13.2%)	32 (18.8%)	.
Small problem	1322 (10.4%)	1254 (10.4%)	46 (8.8%)	22 (12.9%)	.
Moderate problem	813 (6.4%)	773 (6.4%)	33 (6.3%)	7 (4.1%)	.
Big problem	295 (2.3%)	268 (2.2%)	23 (4.4%)	4 (2.4%)	.

Statistic Variable or Category	TNGR Global (n=13483)	Clinically Localized (n=12742)	Locally Advanced (n=566)	Unknown (n=175)	P Value
Missing	760	713	42	5	.
How big a problem during the last 4 weeks, if any, has lack of energy been for you?					.
No problem	7499 (58.5%)	7088 (58.5%)	323 (61.3%)	88 (51.2%)	0.0074
Very small problem	2492 (19.4%)	2356 (19.4%)	86 (16.3%)	50 (29.1%)	.
Small problem	1609 (12.6%)	1538 (12.7%)	55 (10.4%)	16 (9.3%)	.
Moderate problem	889 (6.9%)	832 (6.9%)	44 (8.3%)	13 (7.6%)	.
Big problem	326 (2.5%)	302 (2.5%)	19 (3.6%)	5 (2.9%)	.
Missing	668	626	39	3	.
How big a problem during the last 4 weeks, if any, has change in body weight been for you?					.
No problem	10473 (82.0%)	9905 (82.0%)	426 (81.6%)	142 (83.0%)	0.5285
Very small problem	1160 (9.1%)	1103 (9.1%)	40 (7.7%)	17 (9.9%)	.
Small problem	649 (5.1%)	613 (5.1%)	28 (5.4%)	8 (4.7%)	.
Moderate problem	351 (2.7%)	330 (2.7%)	18 (3.4%)	3 (1.8%)	.
Big problem	136 (1.1%)	125 (1.0%)	10 (1.9%)	1 (0.6%)	.
Missing	714	666	44	4	.
